# Supplementary material for: Docosahexaenoic Acid Ameliorates Contextual Fear Memory Deficits in the Tg2576 Alzheimer’s Disease Mouse Model: Cellular and Molecular Correlates
Source: Pharmaceutics. 2022 Dec 27;15(1):82. doi: 10.3390/pharmaceutics15010082 (PMC9866126; doi:10.3390/pharmaceutics15010082)
Supplement: Supplementary file 1 [file pharmaceutics-15-00082-s001.zip › Figure S1.pdf]

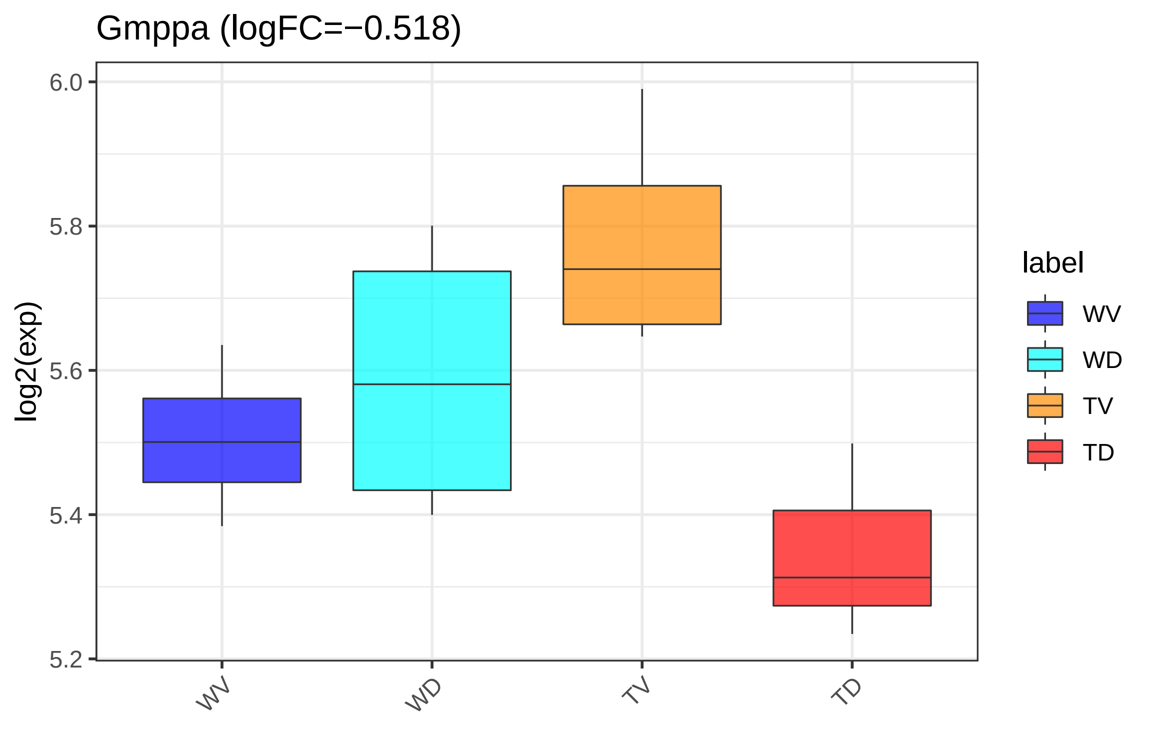
Gmppa (logFC=−0.518)
log2(exp)
6.0
5.8
5.6
5.4
5.2
WV
WD
TV
TD
label
WV
WD
TV
TD

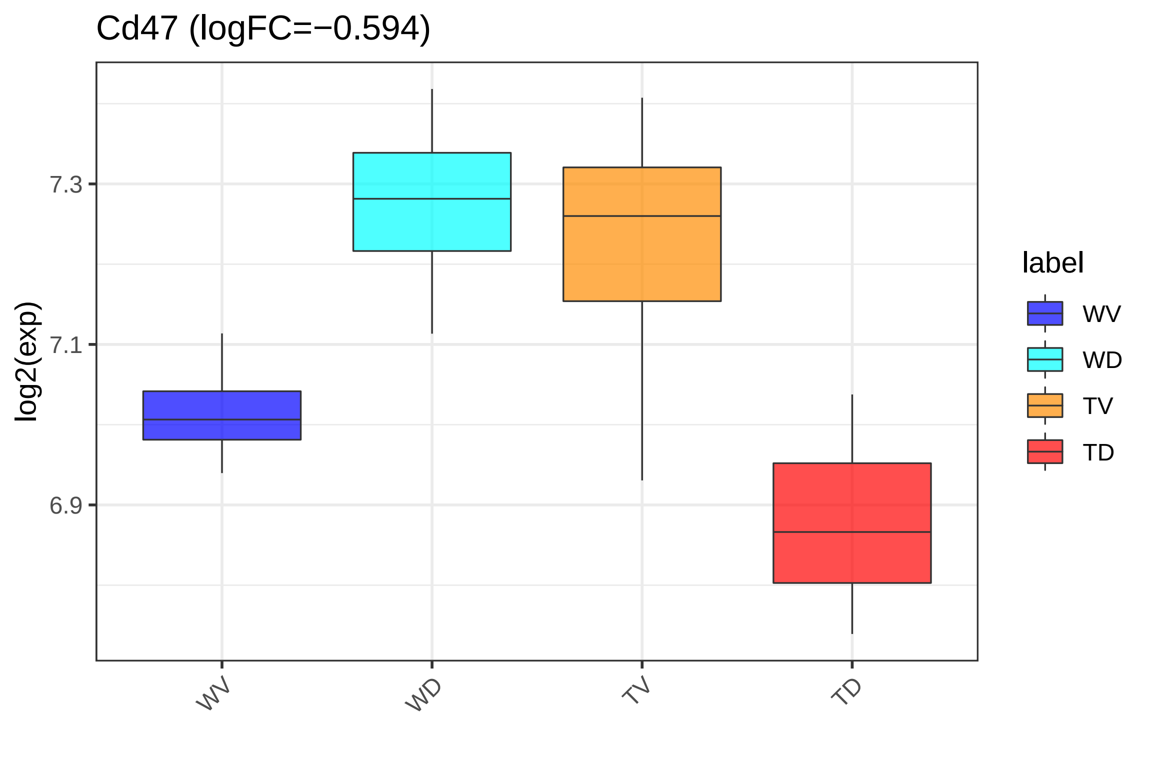
Cd47 (logFC=−0.594)
log2(exp)
7.3
7.1
6.9
WV
WD
TV
TD
label
WV
WD
TV
TD

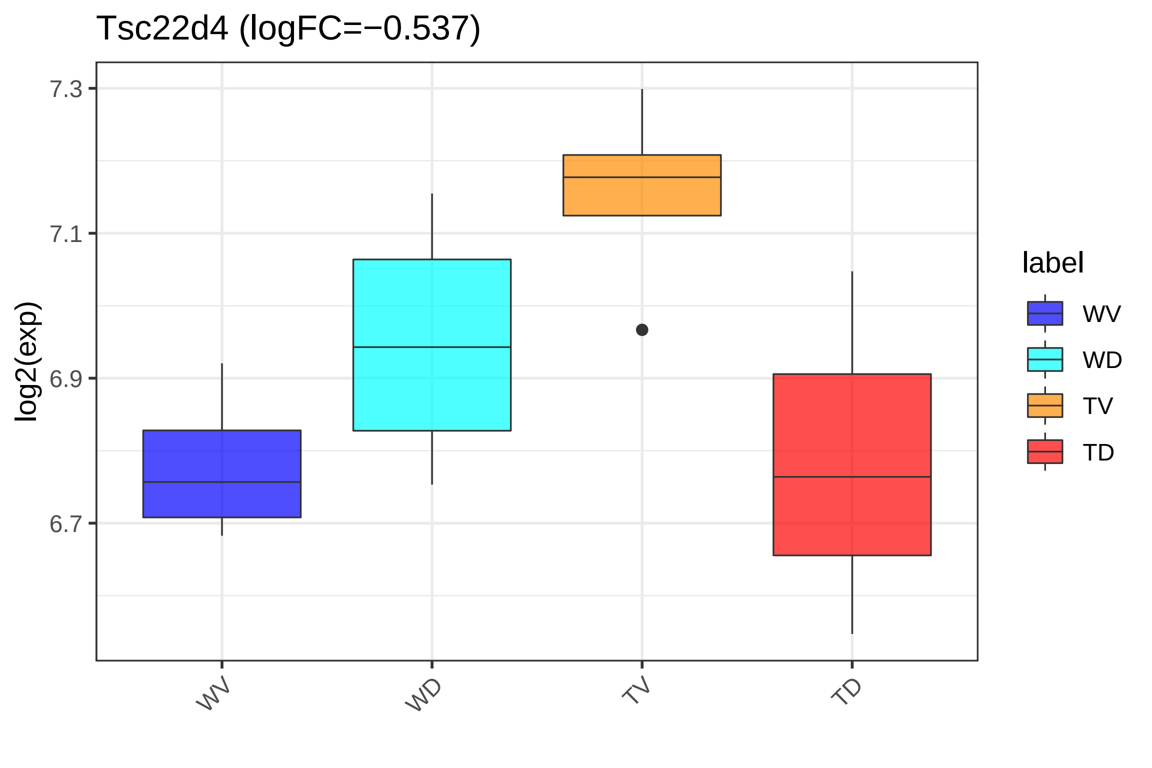

Tsc22d4 (logFC=−0.537)
log2(exp)
7.3
7.1
6.9
6.7
WV
WD
TV
TD
label
WV
WD
TV
TD

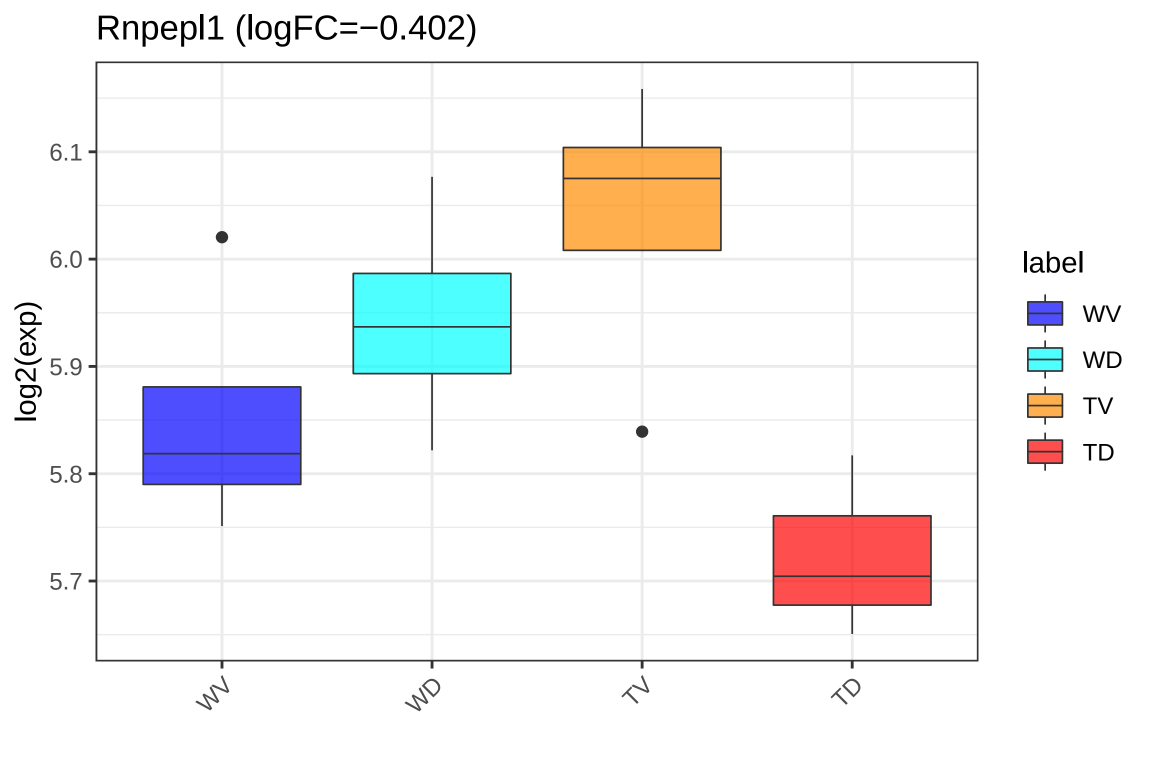

Rnpepl1 (logFC=−0.402)
log2(exp)
6.1
6.0
5.9
5.8
5.7
WV
WD
TV
TD
label
WV
WD
TV
TD

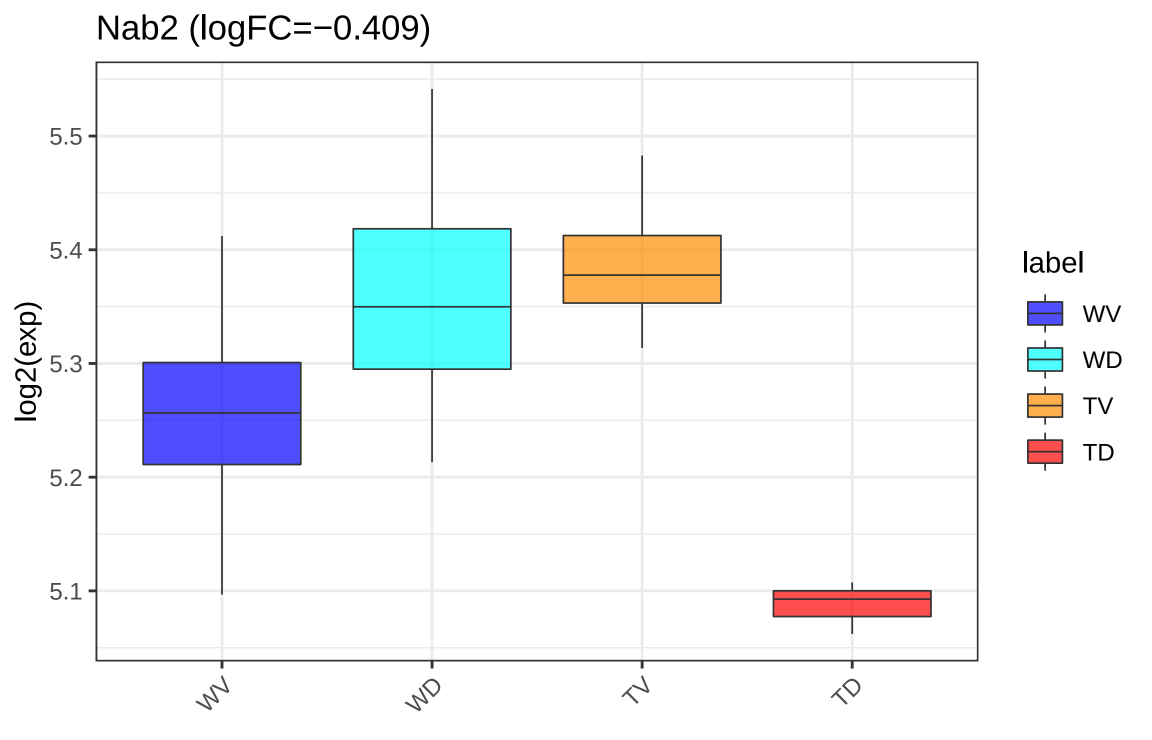

Nab2 (logFC=−0.409)
log2(exp)
5.5
5.4
5.3
5.2
5.1
WV
WD
TV
TD
label
WV
WD
TV
TD

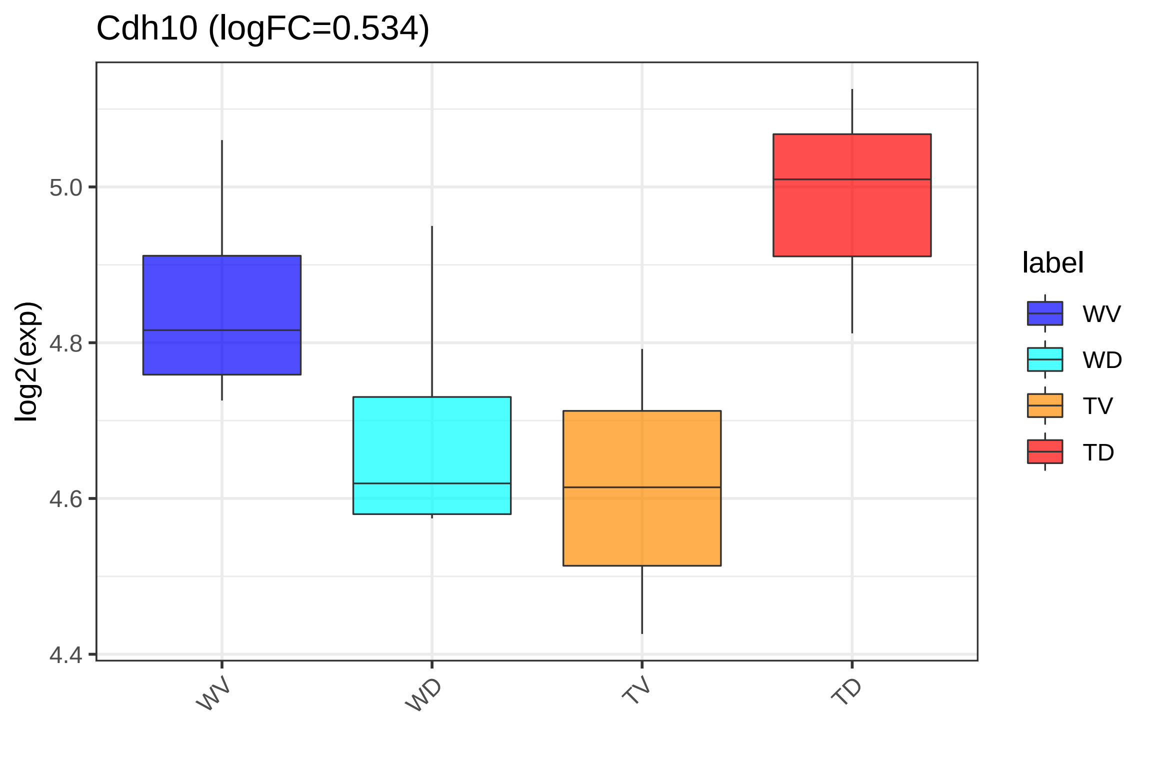

Cdh10 (logFC=0.534)
log2(exp)
5.0
4.8
4.6
4.4
WV
WD
TV
TD
label
WV
WD
TV
TD

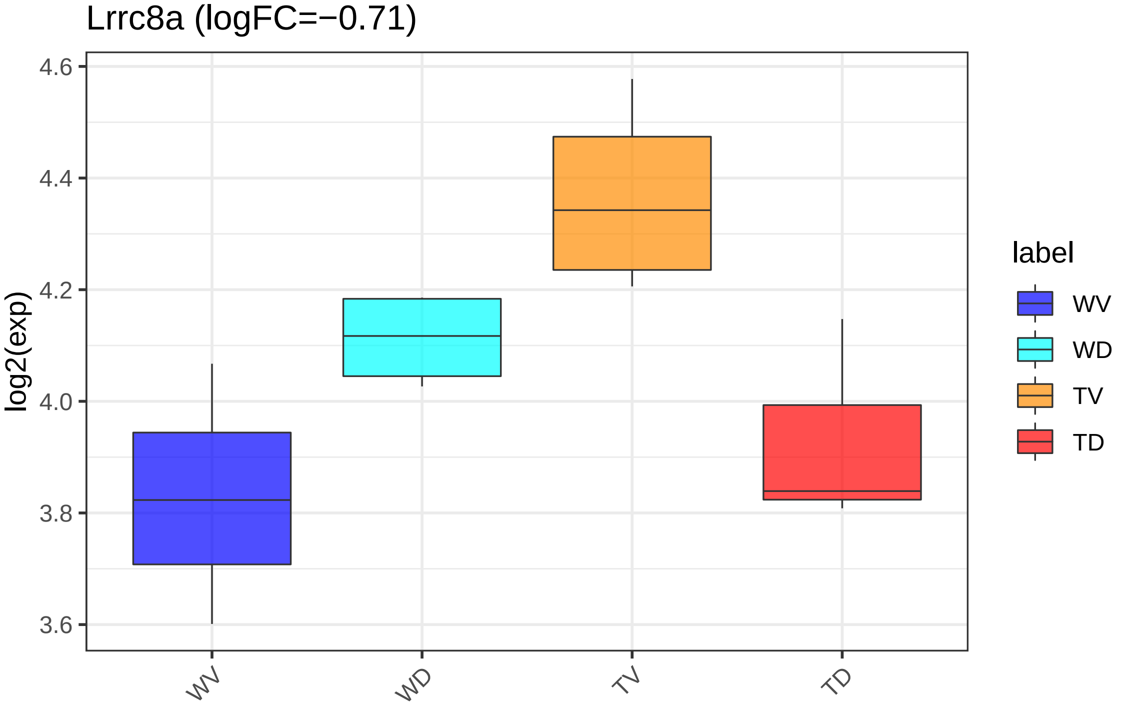

Lrrc8a (logFC=−0.71)
log2(exp)
4.6
4.4
4.2
4.0
3.8
3.6
WV
WD
TV
TD
label
WV
WD
TV
TD

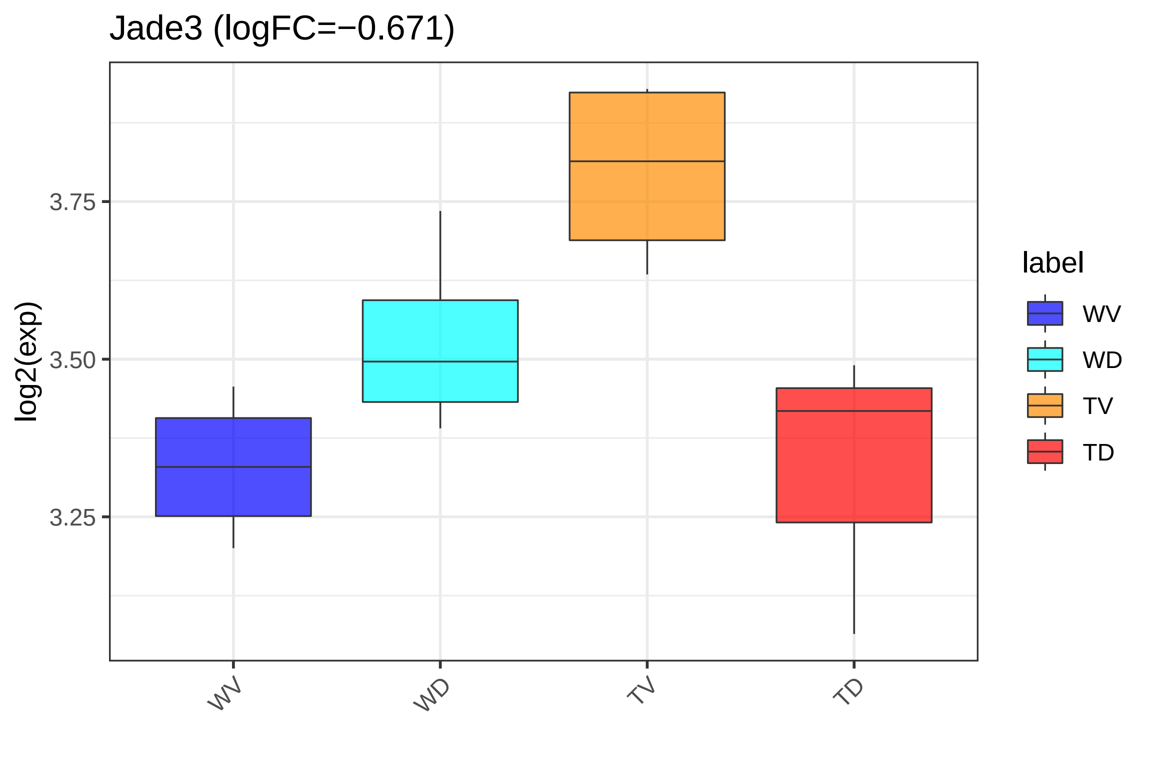
Jade3 (logFC=−0.671)
log2(exp)
3.75
3.50
3.25
WV
WD
TV
TD
label
WV
WD
TV
TD

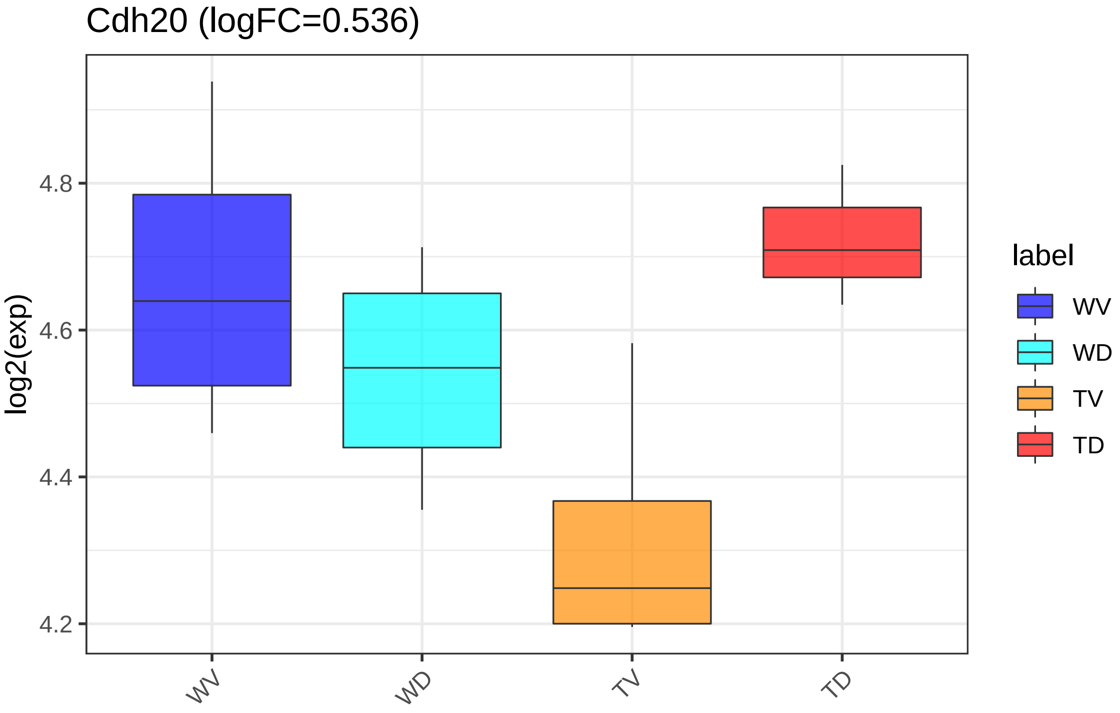

Cdh20 (logFC=0.536)
log2(exp)
4.8
4.6
4.4
4.2
WV
WD
TV
TD
label
WV
WD
TV
TD

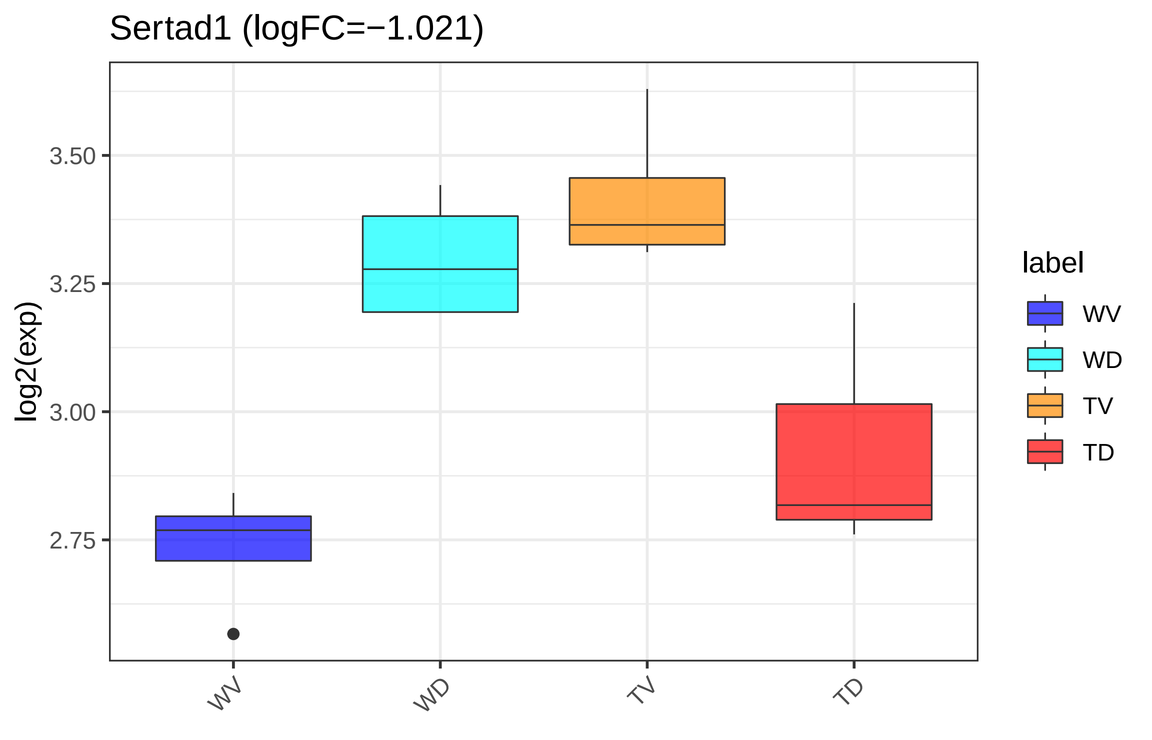
Sertad1 (logFC=−1.021)
log2(exp)
3.50
3.25
3.00
2.75
WV
WD
TV
TD
label
WV
WD
TV
TD

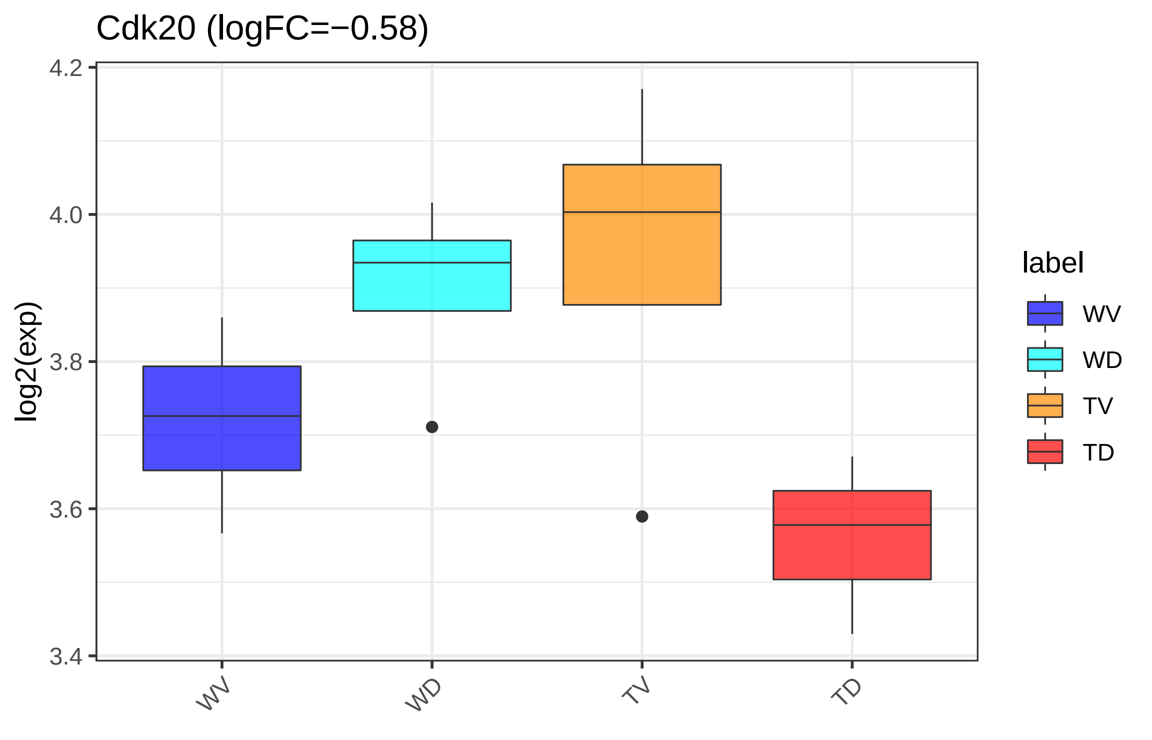
Cdk20 (logFC=−0.58)
log2(exp)
4.2
4.0
3.8
3.6
3.4
WV
WD
TV
TD
label
WV
WD
TV
TD

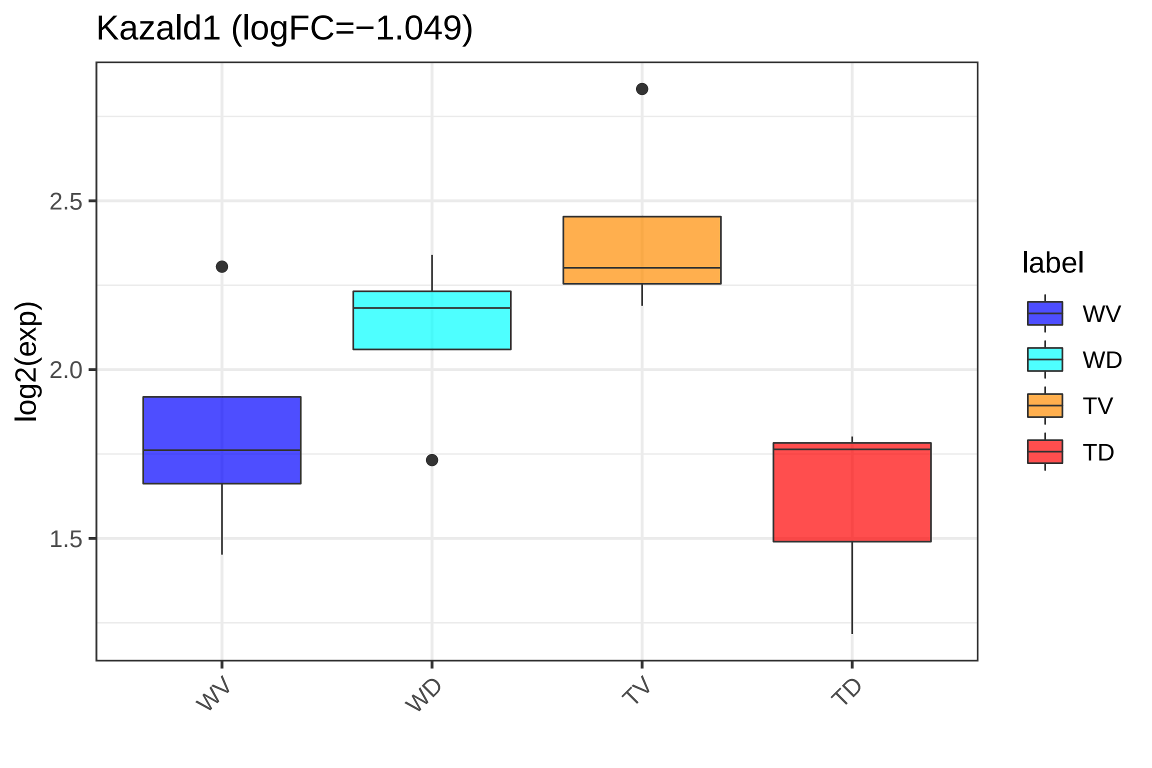

Kazald1 (logFC=−1.049)
log2(exp)
2.5
2.0
1.5
WV
WD
TV
TD
label
WV
WD
TV
TD

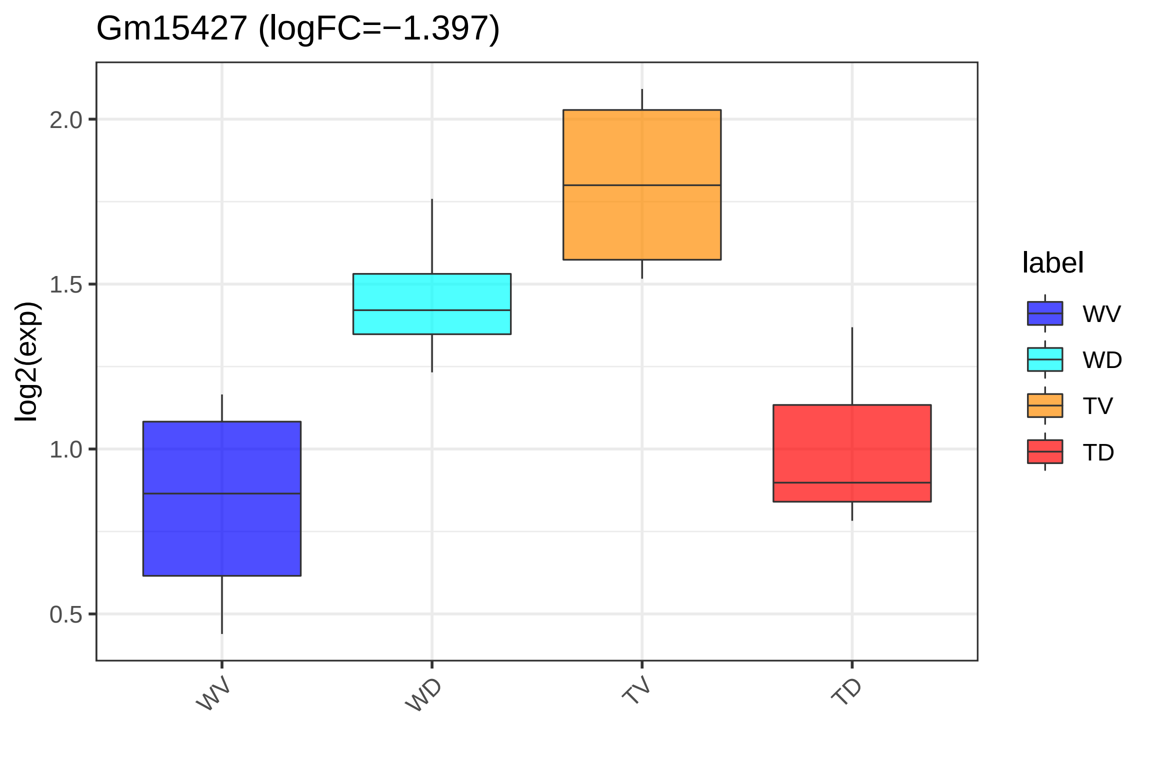
Gm15427 (logFC=−1.397)
log2(exp)
2.0
1.5
1.0
0.5
WV
WD
TV
TD
label
WV
WD
TV
TD

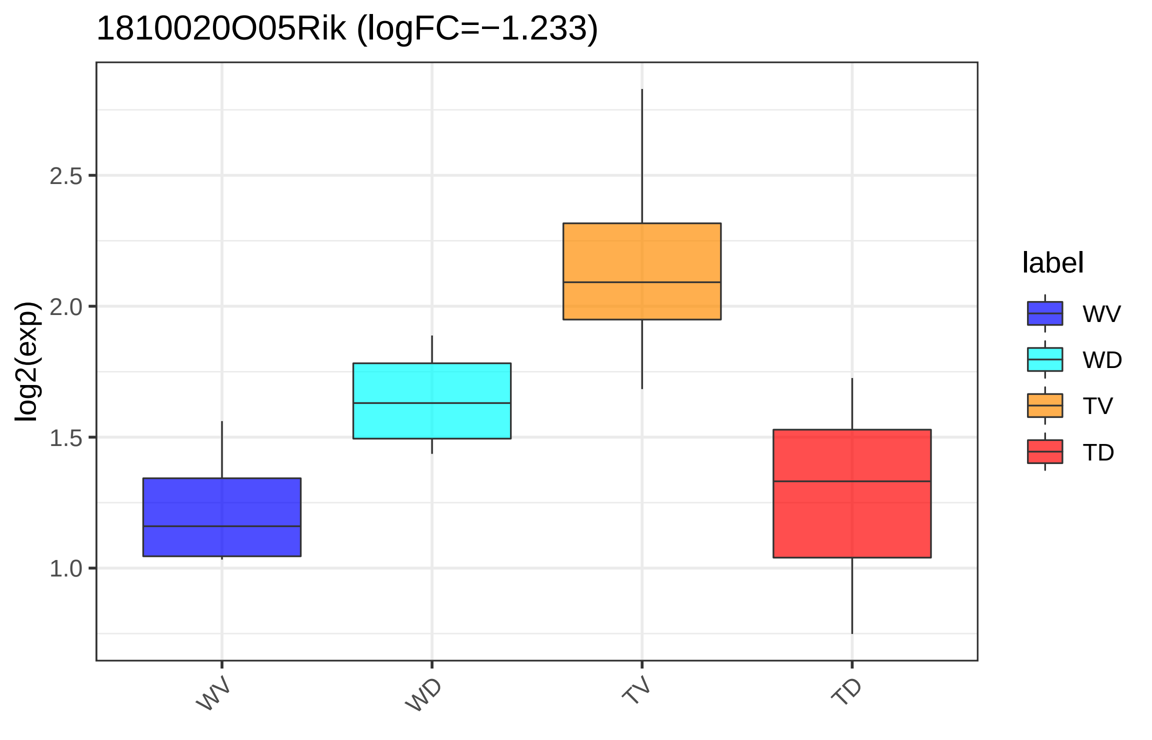
1810020O05Rik (logFC=−1.233)
log2(exp)
2.5
2.0
1.5
1.0
WV
WD
TV
TD
label
WV
WD
TV
TD

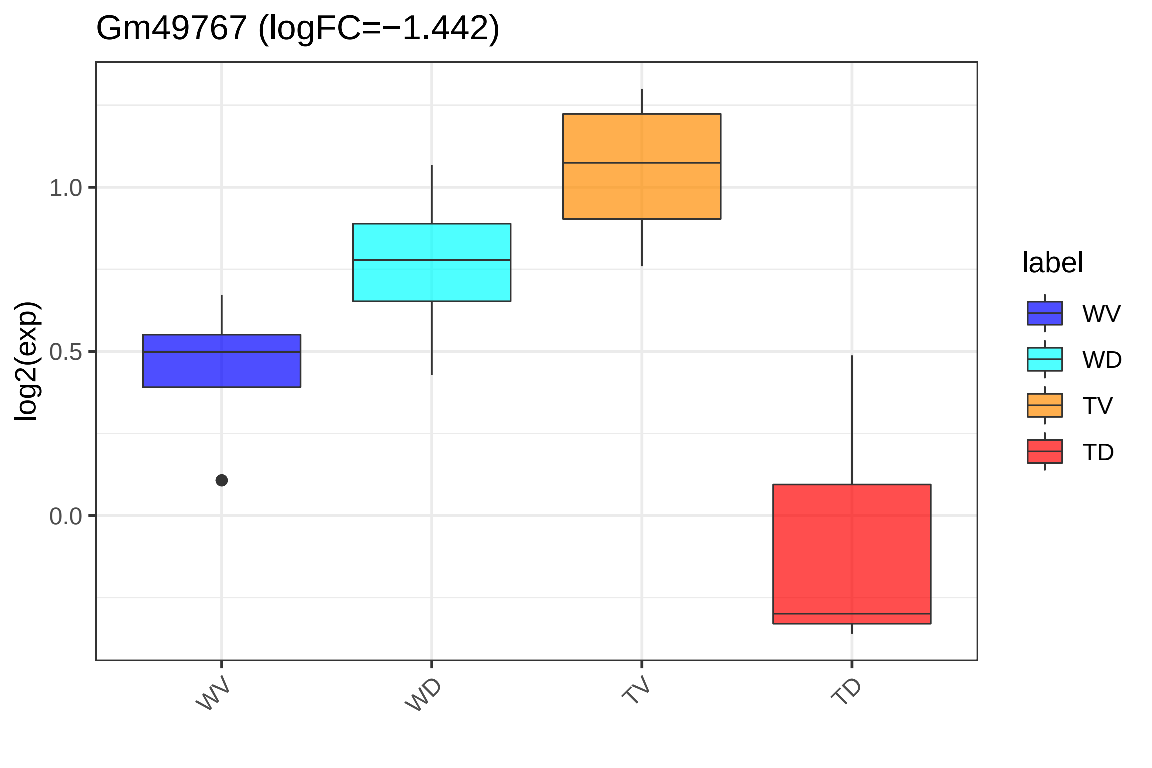

Gm49767 (logFC=−1.442)
log2(exp)
1.0
0.5
0.0
WV
WD
TV
TD
label
WV
WD
TV
TD

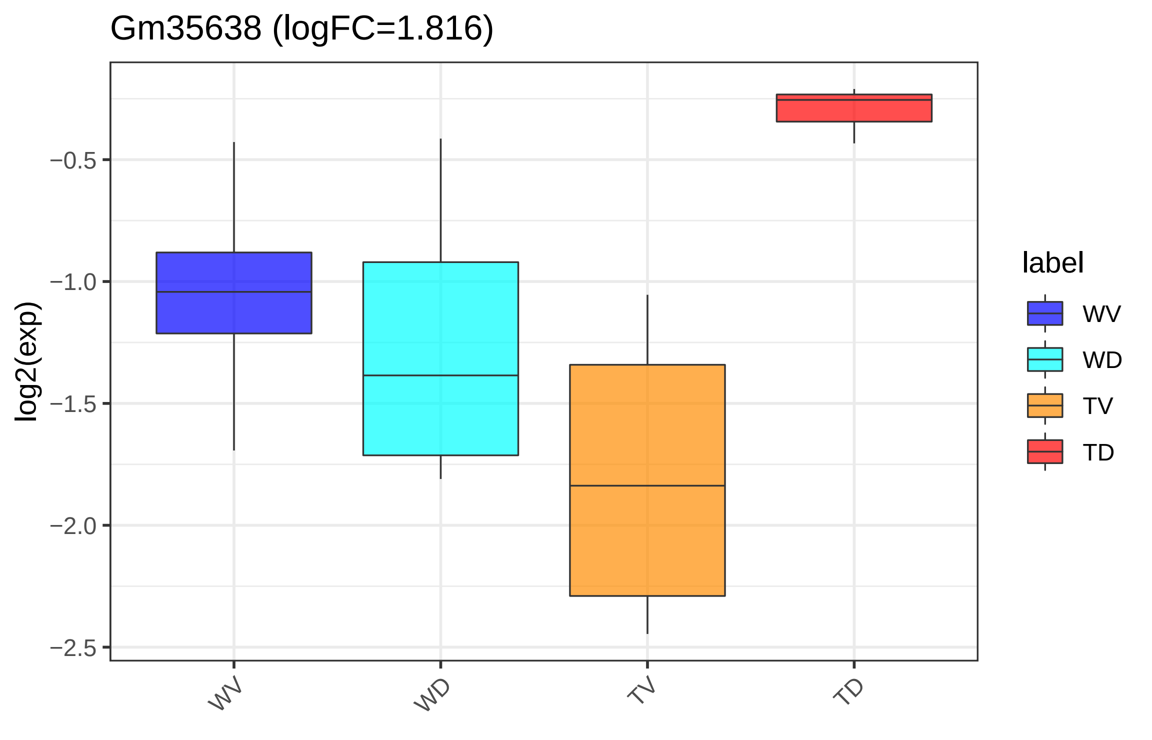

Gm35638 (logFC=1.816)
log2(exp)
−0.5
−1.0
−1.5
−2.0
−2.5
WV
WD
TV
TD
label
WV
WD
TV
TD

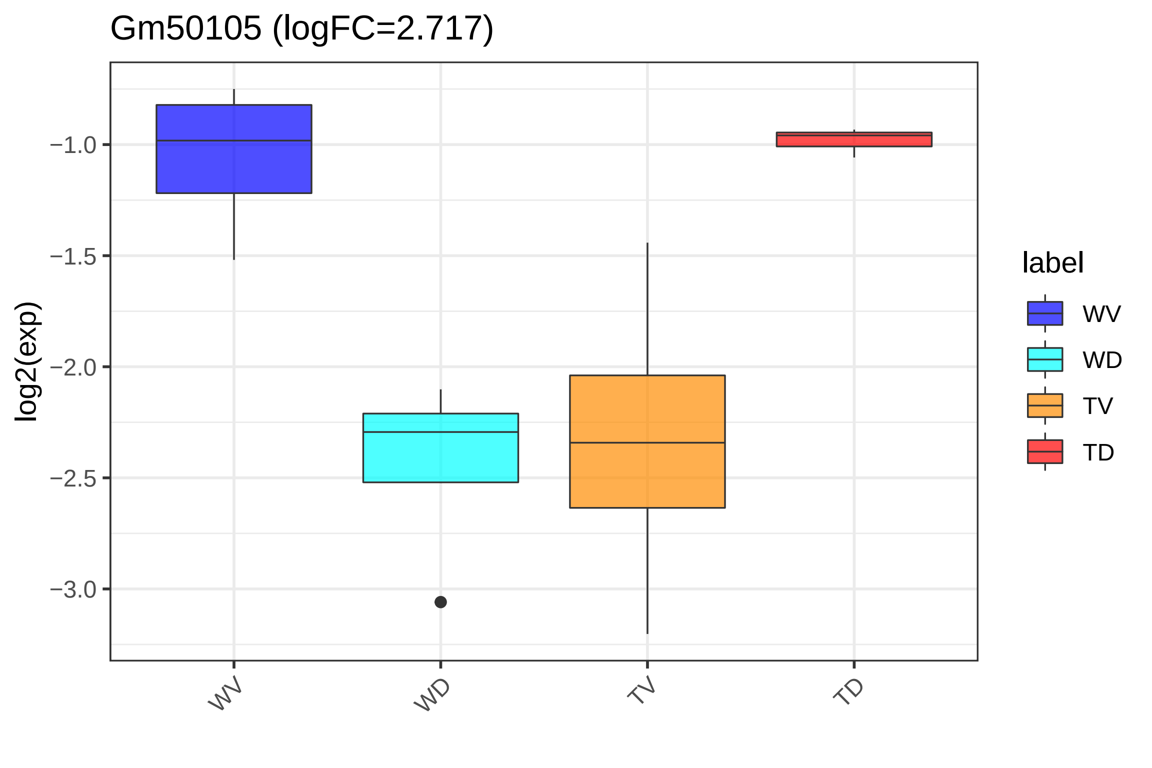

Gm50105 (logFC=2.717)
log2(exp)
−1.0
−1.5
−2.0
−2.5
−3.0
WV
WD
TV
TD
label
WV
WD
TV
TD

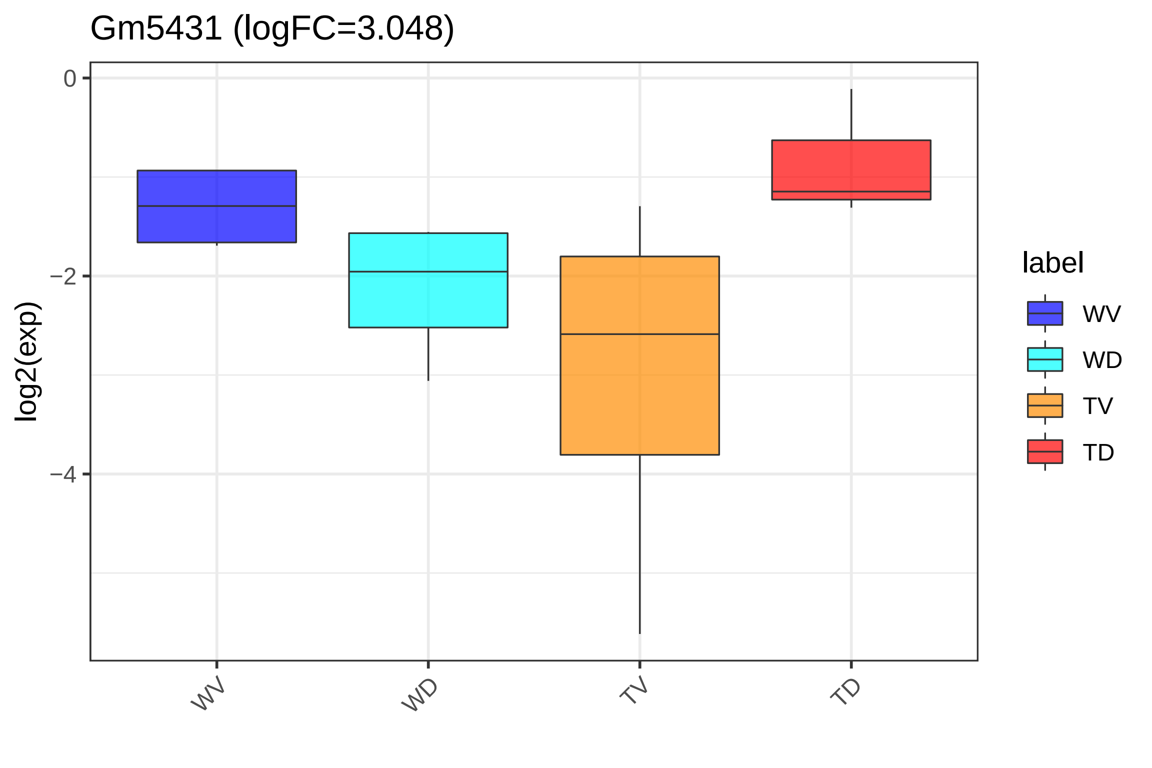

Gm5431 (logFC=3.048)
0
−2
−4
log2(exp)
WV
WD
TV
TD
label
WV
WD
TV
TD

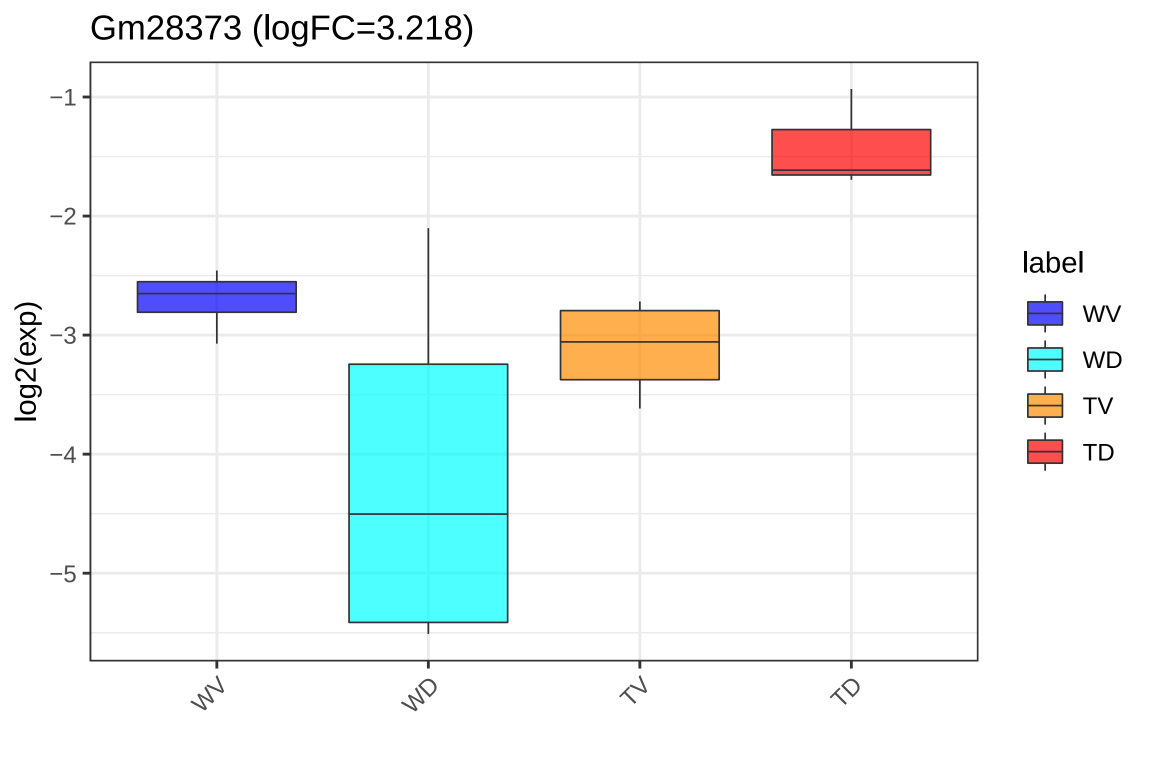

Gm28373 (logFC=3.218)
log2(exp)
−1
−2
−3
−4
−5
WV
WD
TV
TD
label
WV
WD
TV
TD

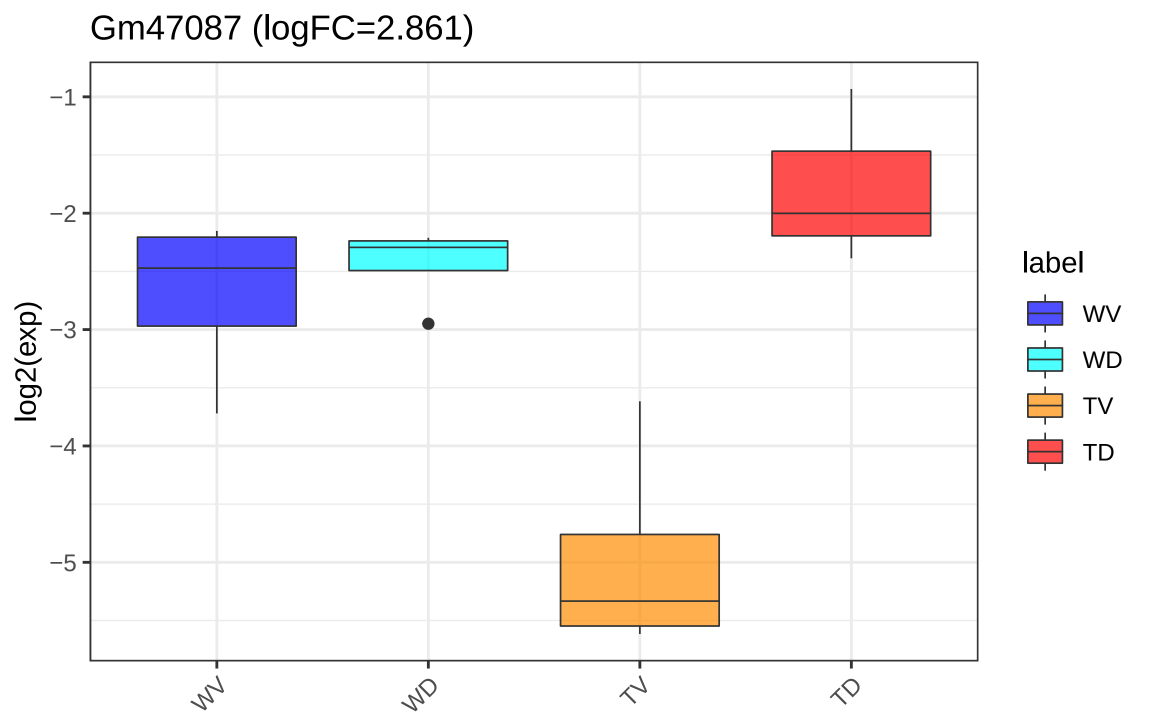

Gm47087 (logFC=2.861)
log2(exp)
−1
−2
−3
−4
−5
WV
WD
TV
TD
label
WV
WD
TV
TD

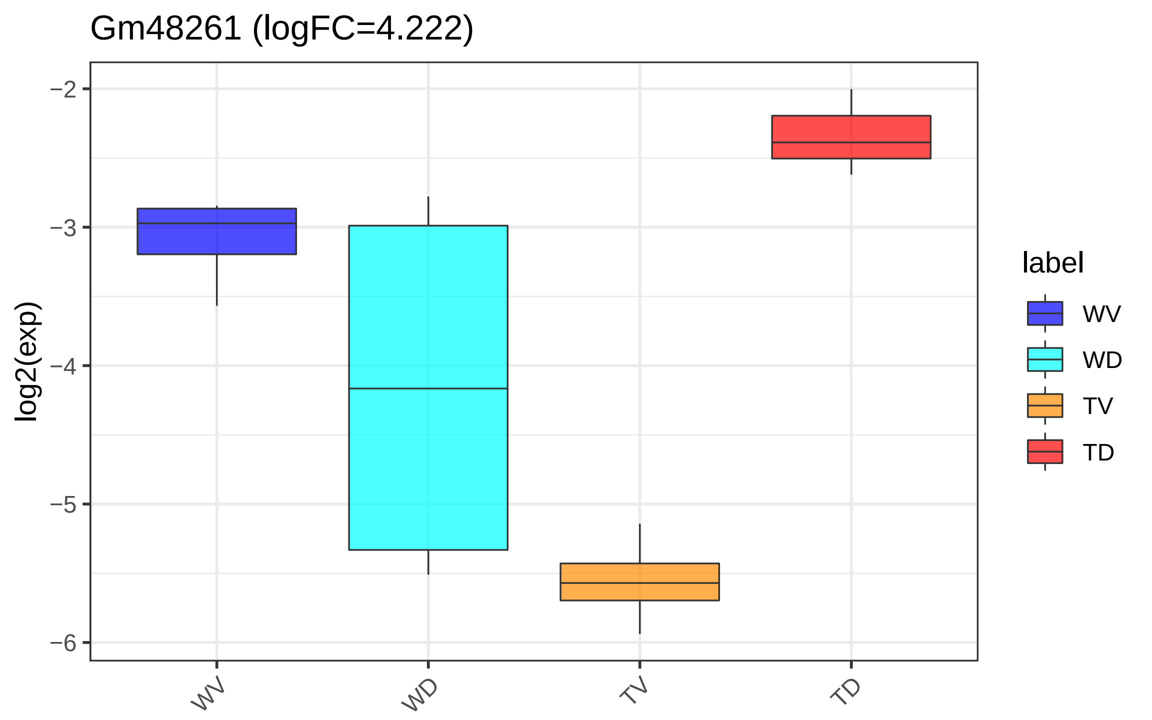

Gm48261 (logFC=4.222)
log2(exp)
−2
−3
−4
−5
−6
WV
WD
TV
TD
label
WV
WD
TV
TD

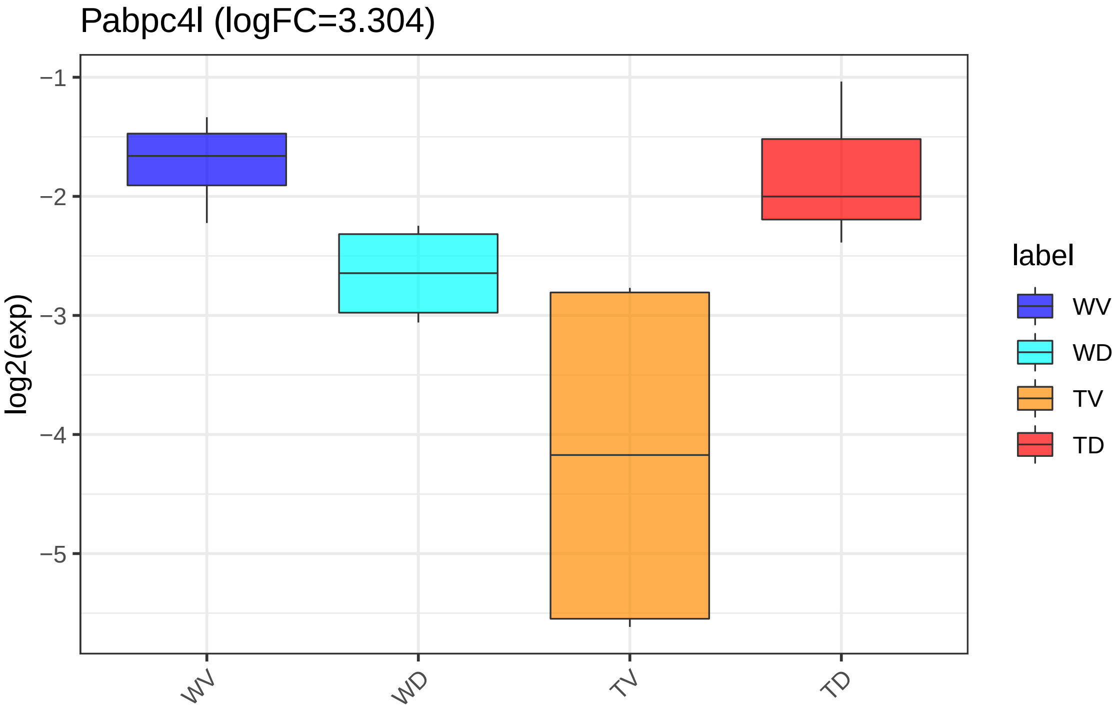

Pabpc4l (logFC=3.304)
log2(exp)
−1
−2
−3
−4
−5
WV
WD
TV
TD
label
WV
WD
TV
TD

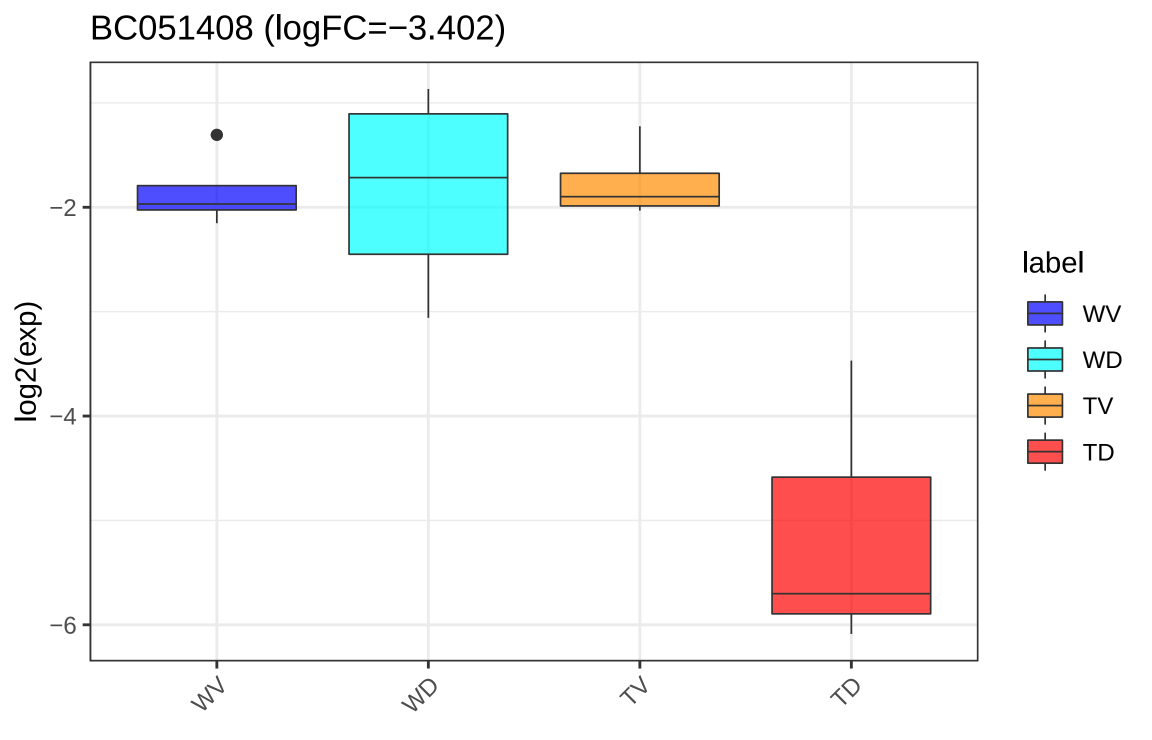
BC051408 (logFC=−3.402)
log2(exp)
−2
−4
−6
WV
WD
TV
TD
label
WV
WD
TV
TD

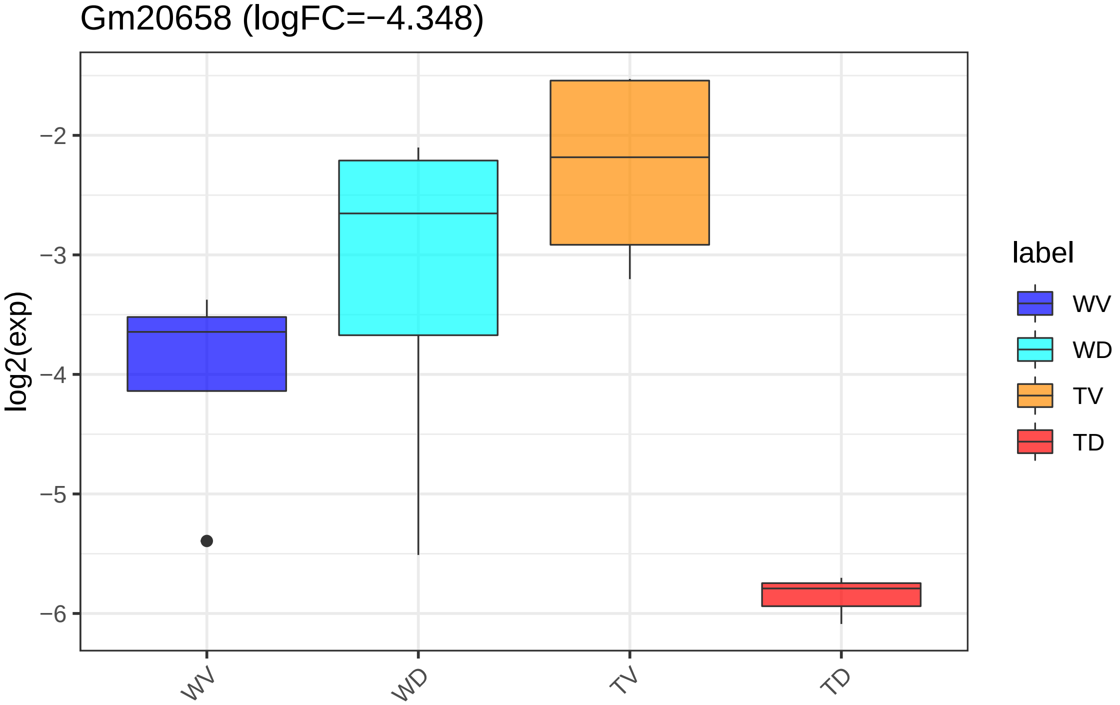
Gm20658 (logFC=−4.348)
log2(exp)
−2
−3
−4
−5
−6
WV
WD
TV
TD
label
WV
WD
TV
TD

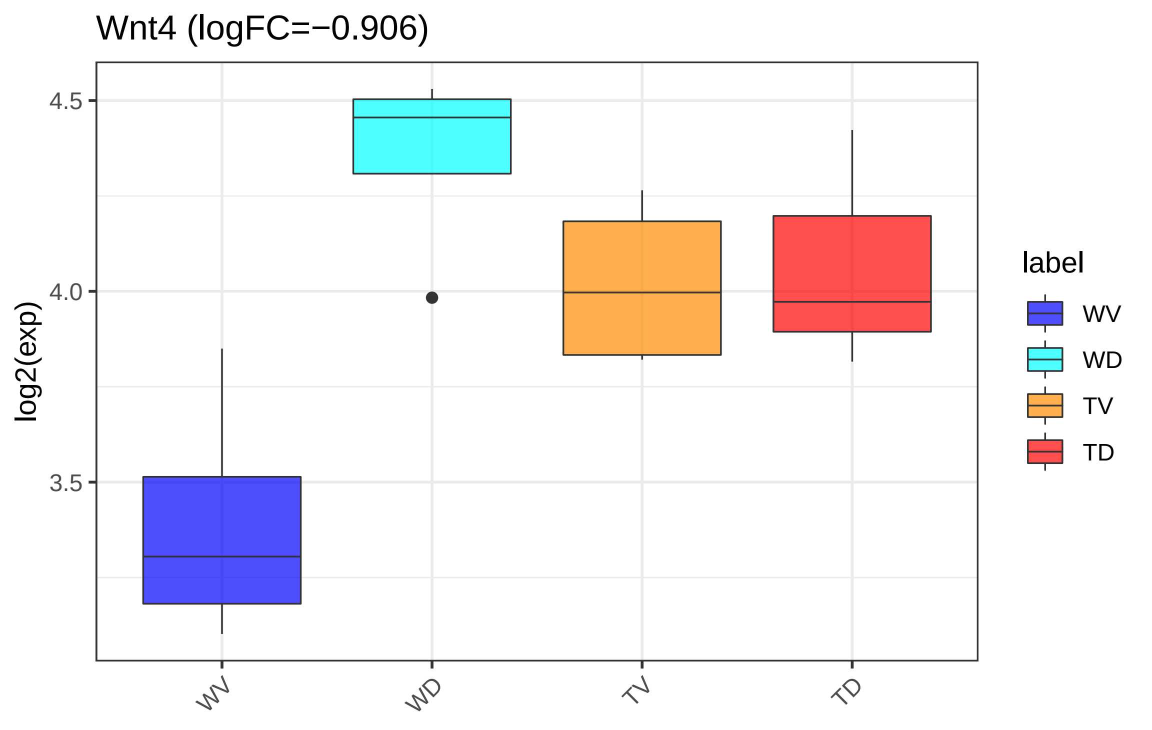
Wnt4 (logFC=−0.906)
log2(exp)
4.5
4.0
3.5
WV
WD
TV
TD
label
WV
WD
TV
TD

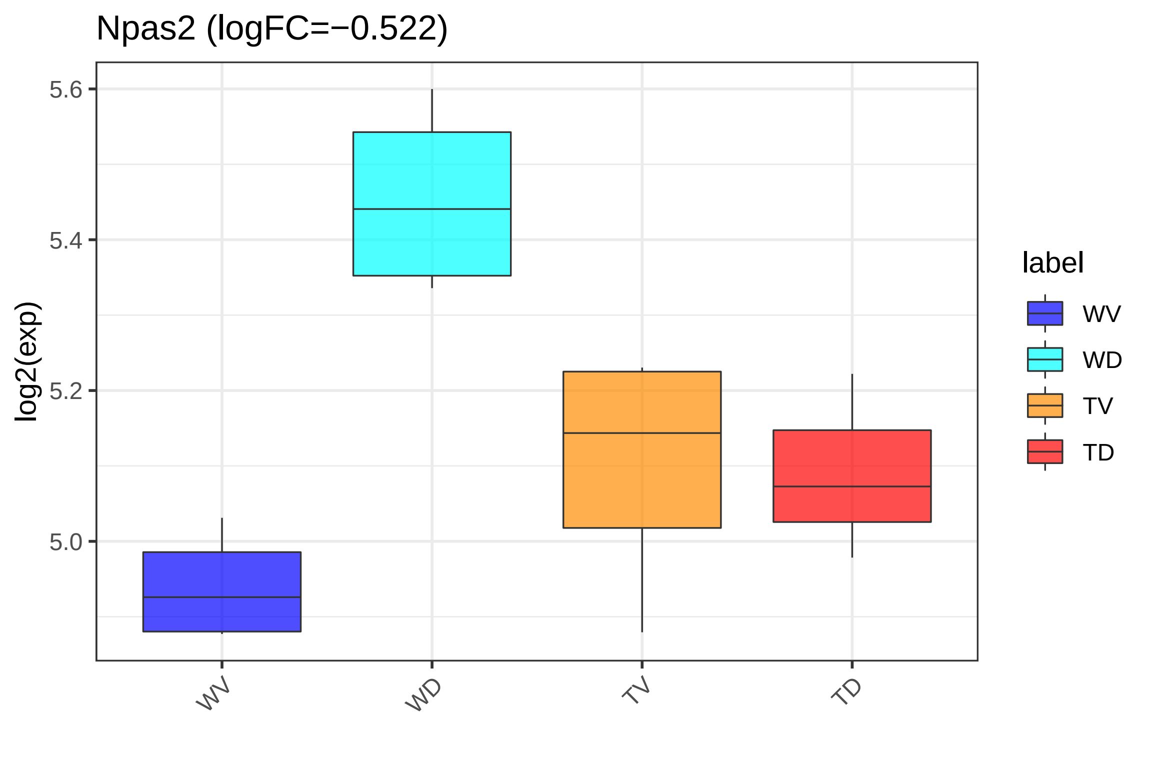

Npas2 (logFC=−0.522)
log2(exp)
5.6
5.4
5.2
5.0
WV
WD
TV
TD
label
WV
WD
TV
TD

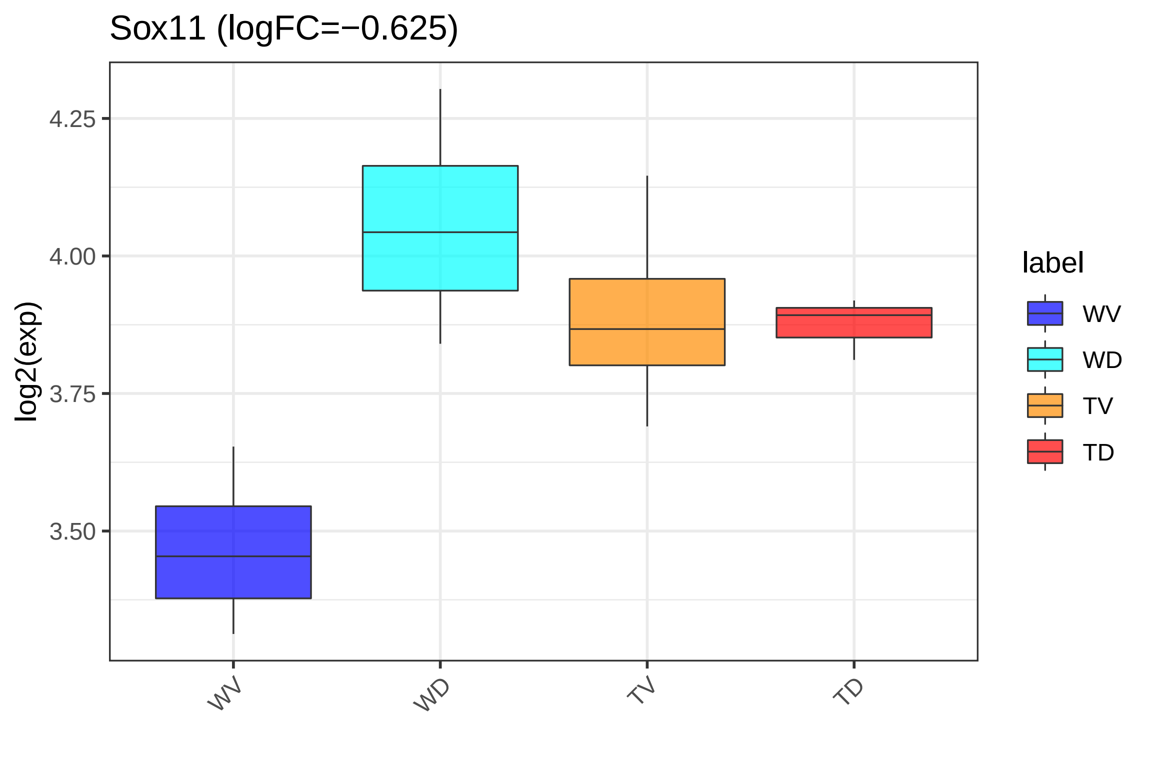

Sox11 (logFC=−0.625)
log2(exp)
4.25
4.00
3.75
3.50
WV
WD
TV
TD
label
WV
WD
TV
TD

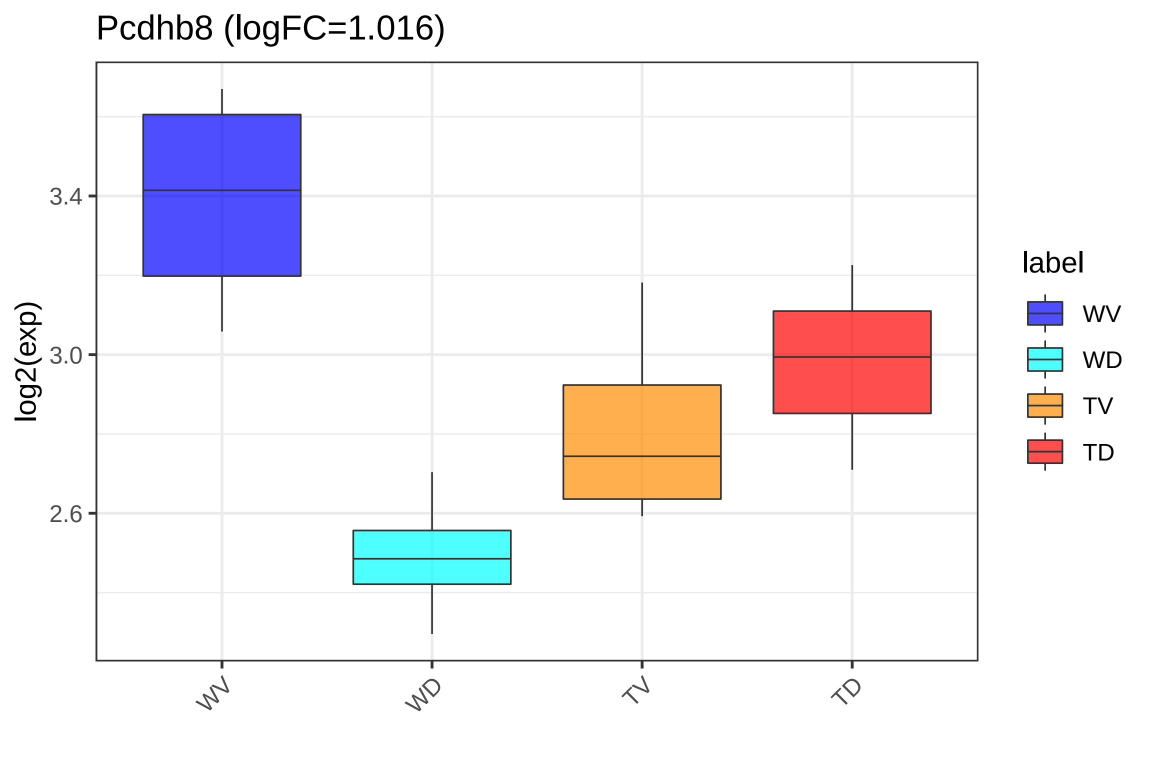
Pcdhb8 (logFC=1.016)
log2(exp)
3.4
3.0
2.6
WV
WD
TV
TD
label
WV
WD
TV
TD

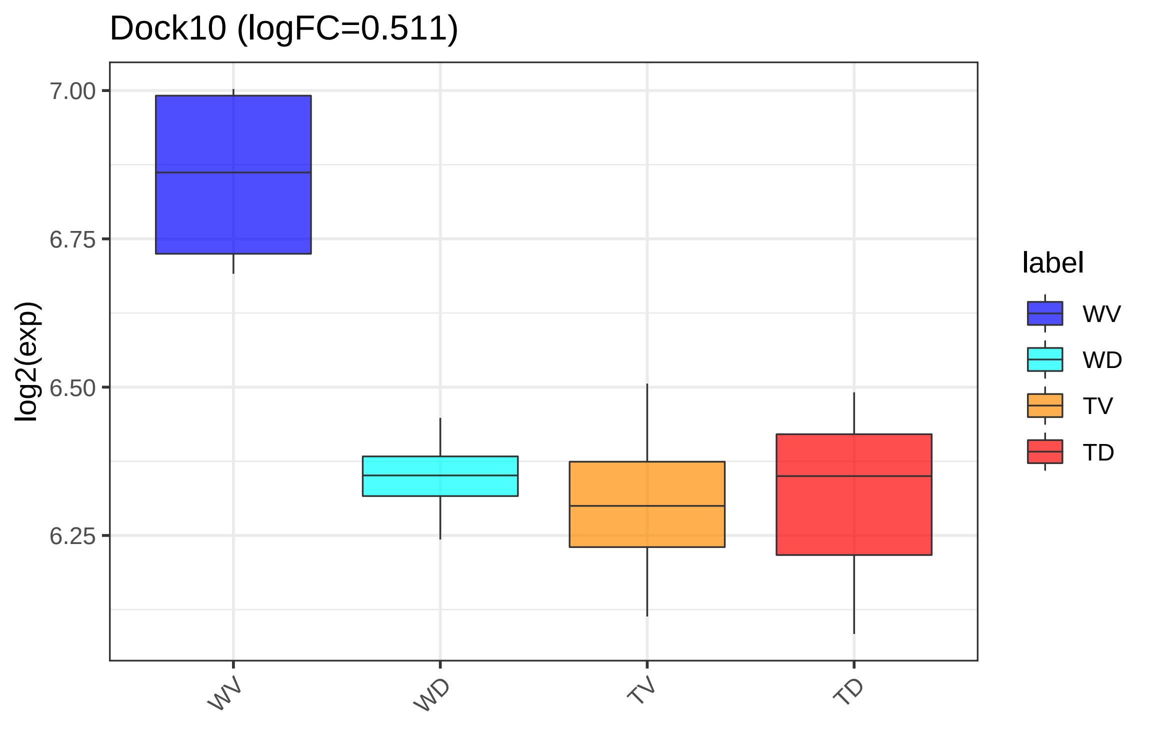

Dock10 (logFC=0.511)
log2(exp)
7.00
6.75
6.50
6.25
WV
WD
TV
TD
label
WV
WD
TV
TD

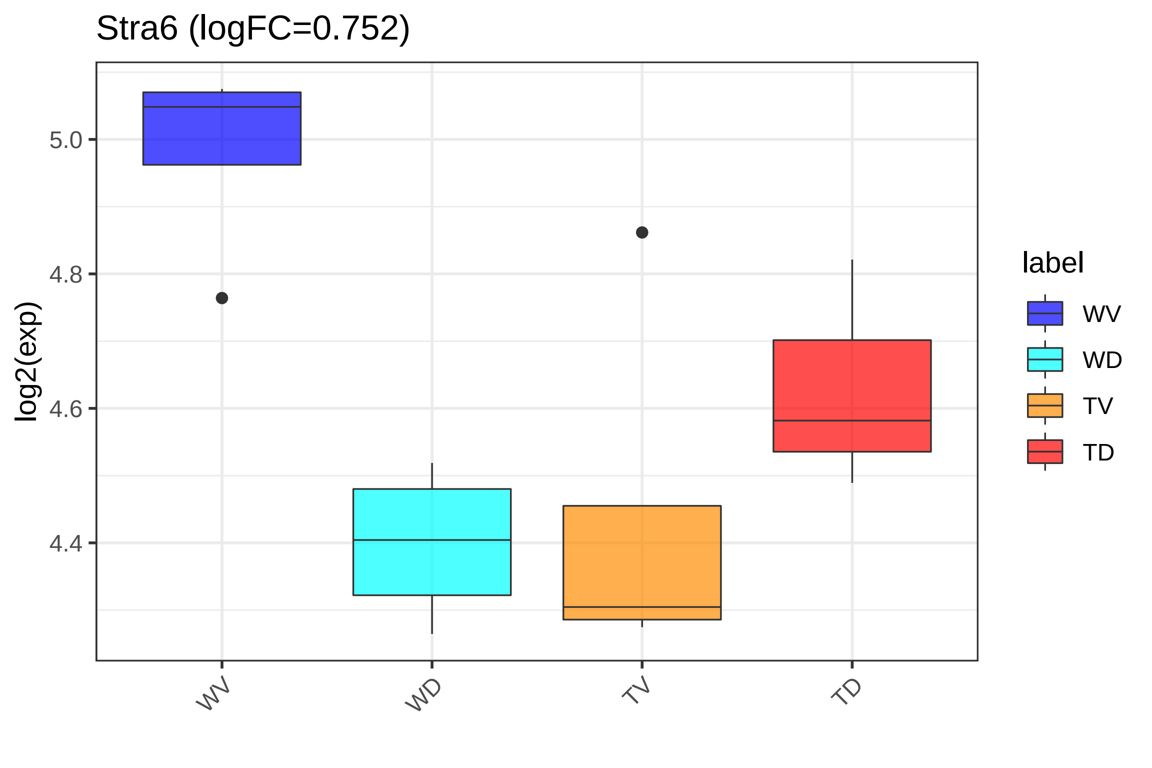

Stra6 (logFC=0.752)
log2(exp)
5.0
4.8
4.6
4.4
WV
WD
TV
TD
label
WV
WD
TV
TD

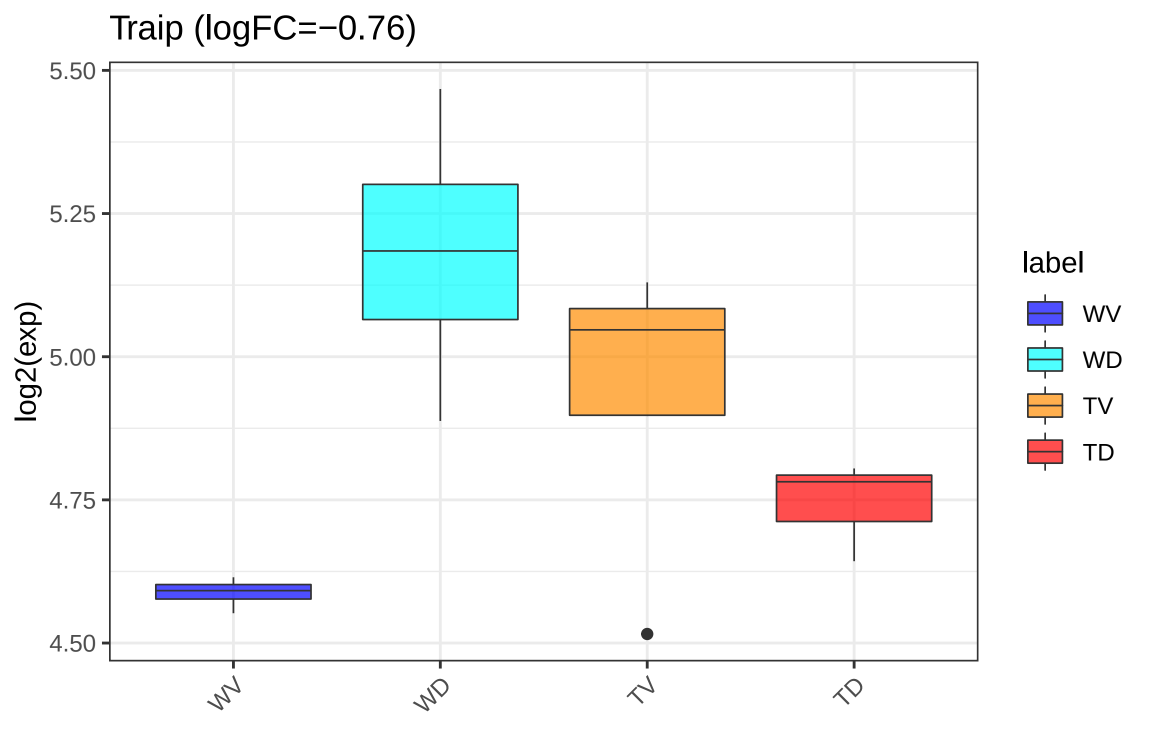
Traip (logFC=−0.76)
log2(exp)
5.50
5.25
5.00
4.75
4.50
WV
WD
TV
TD
label
WV
WD
TV
TD

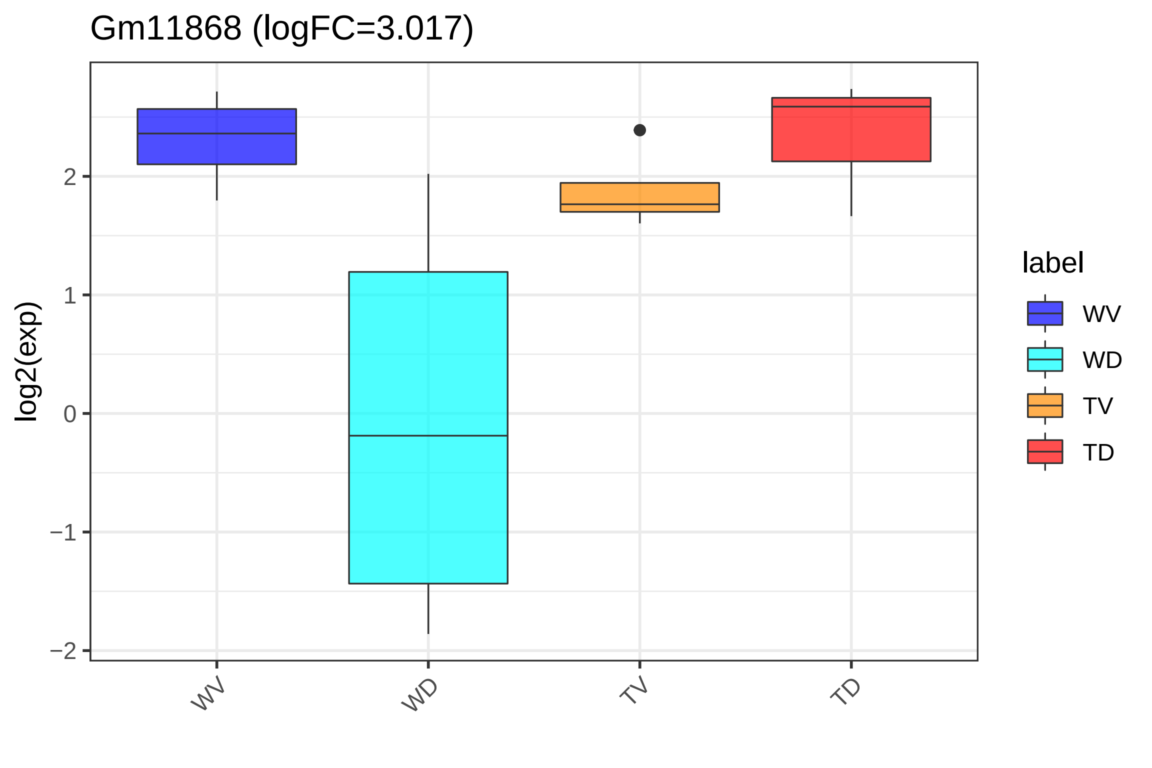

Gm11868 (logFC=3.017)
log2(exp)
2
1
0
−1
−2
WV
WD
TV
TD
label
WV
WD
TV
TD

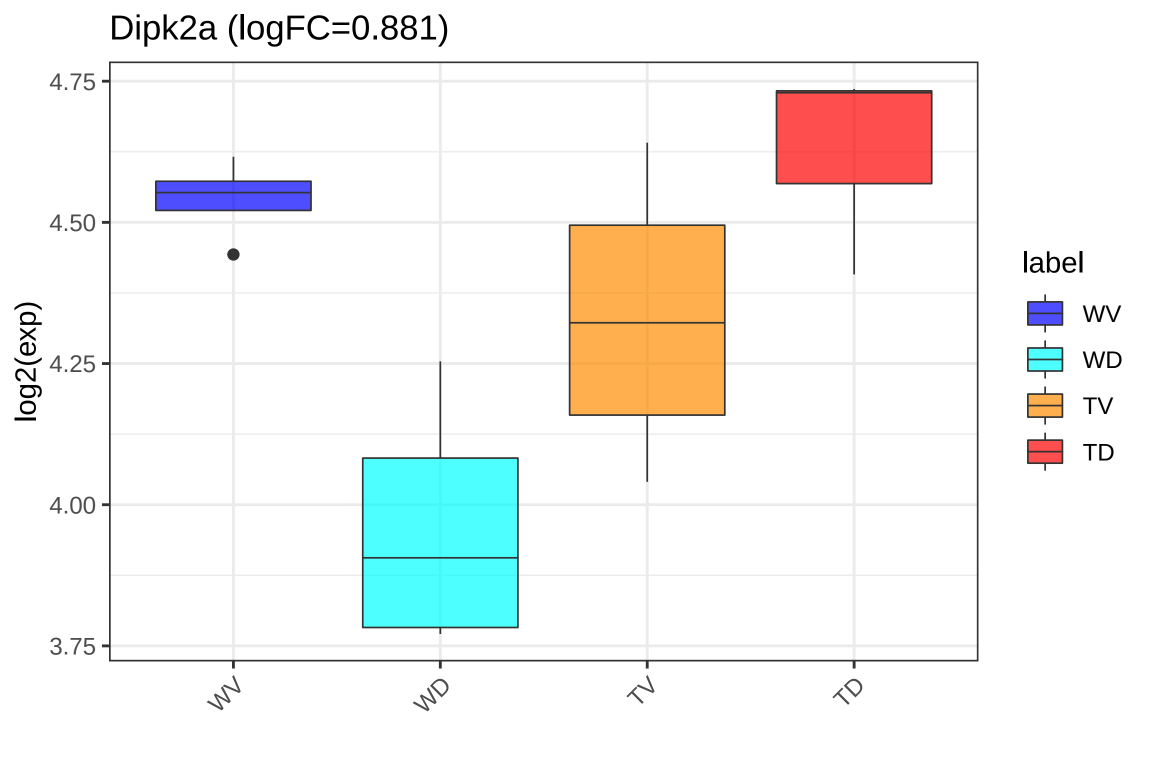

Dipk2a (logFC=0.881)
log2(exp)
4.75
4.50
4.25
4.00
3.75
WV
WD
TV
TD
label
WV
WD
TV
TD

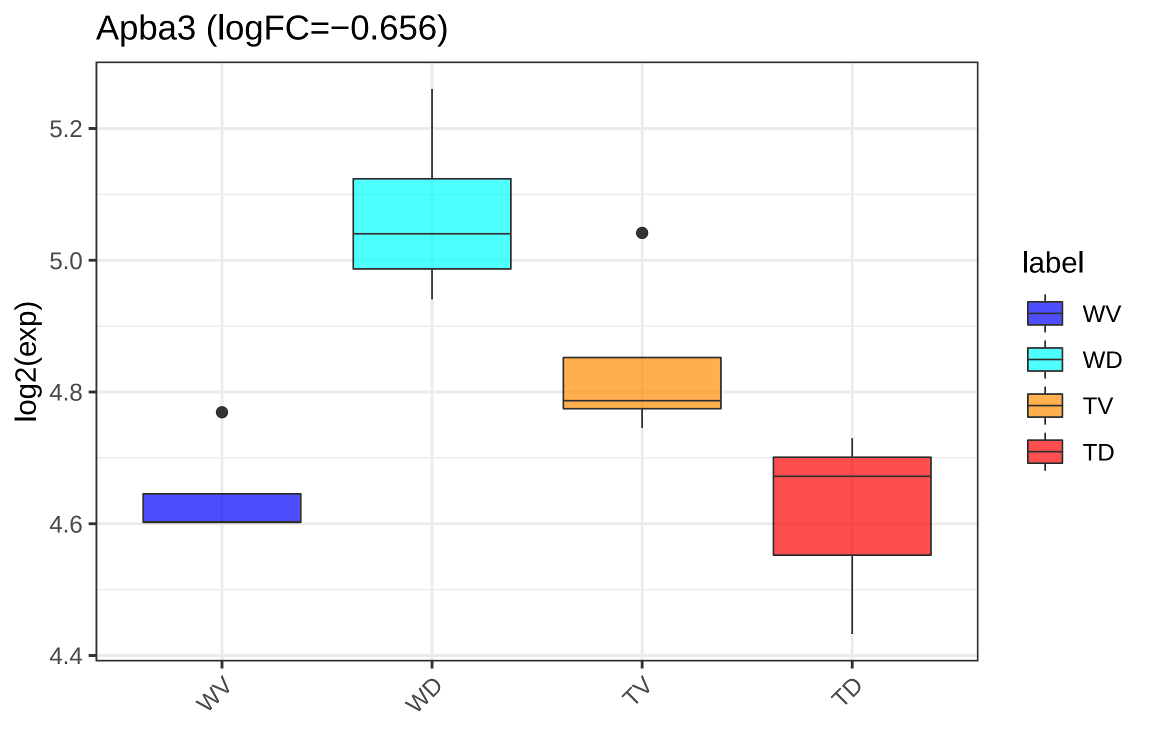
Apba3 (logFC=−0.656)
log2(exp)
5.2
5.0
4.8
4.6
4.4
WV
WD
TV
TD
label
WV
WD
TV
TD

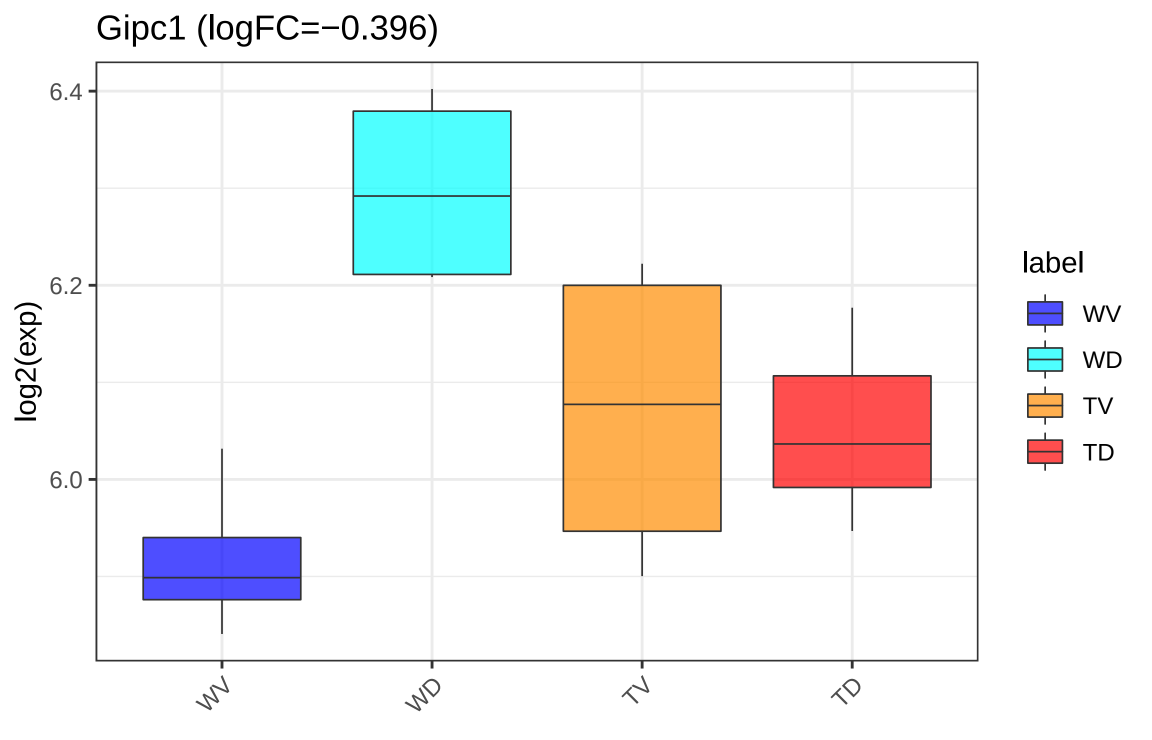

Gipc1 (logFC=−0.396)
6.4
6.2
6.0
log2(exp)
WV
WD
TV
TD
label
WV
WD
TV
TD

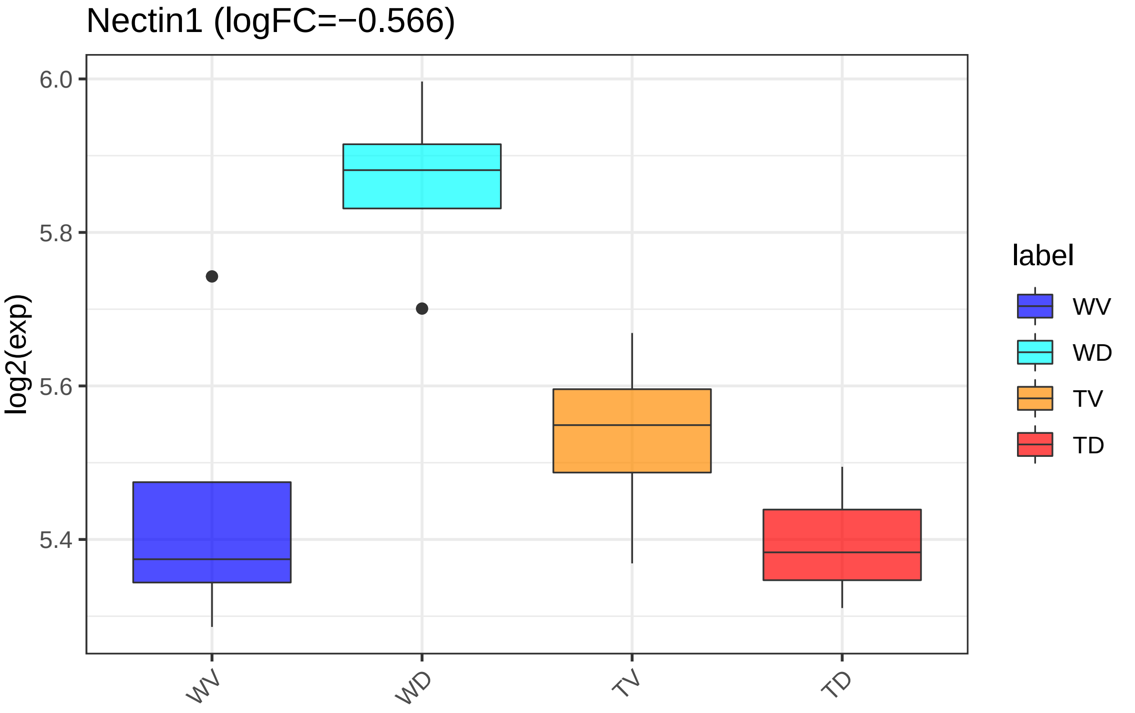

Nectin1 (logFC=−0.566)
log2(exp)
6.0
5.8
5.6
5.4
WV
WD
TV
TD
label
WV
WD
TV
TD

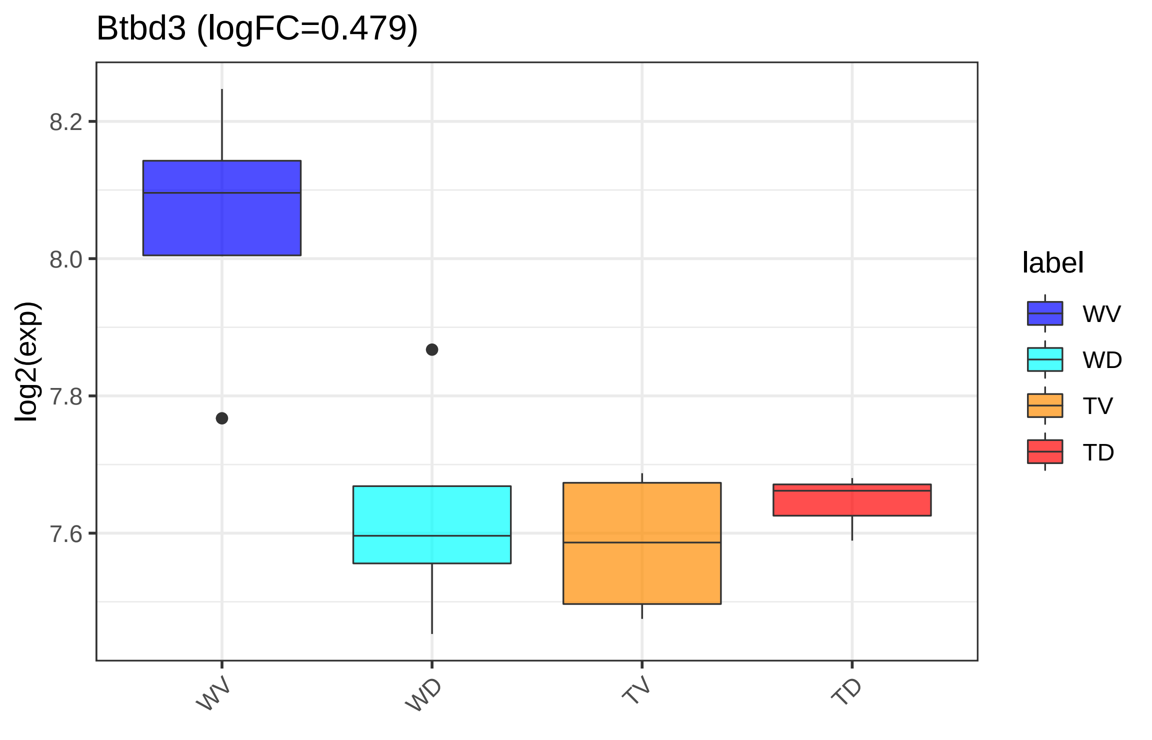

Btbd3 (logFC=0.479)
log2(exp)
8.2
8.0
7.8
7.6
WV
WD
TV
TD
label
WV
WD
TV
TD

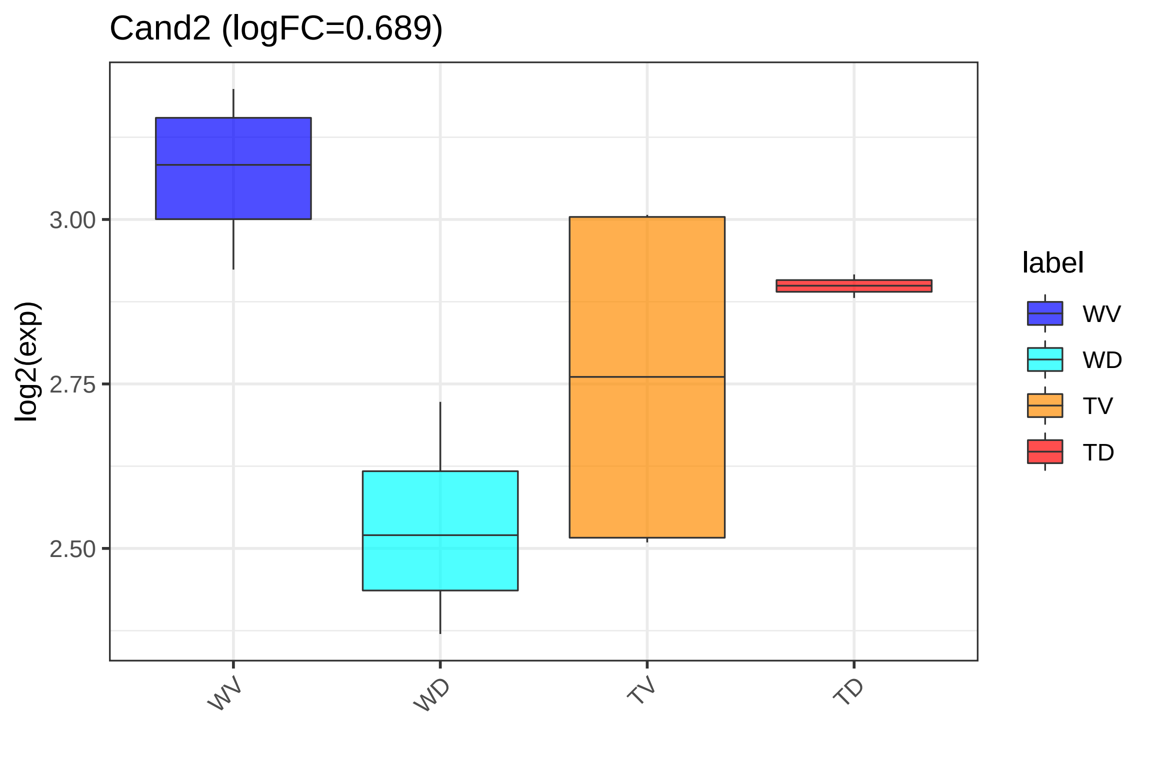

Cand2 (logFC=0.689)
log2(exp)
3.00
2.75
2.50
WV
WD
TV
TD
label
WV
WD
TV
TD

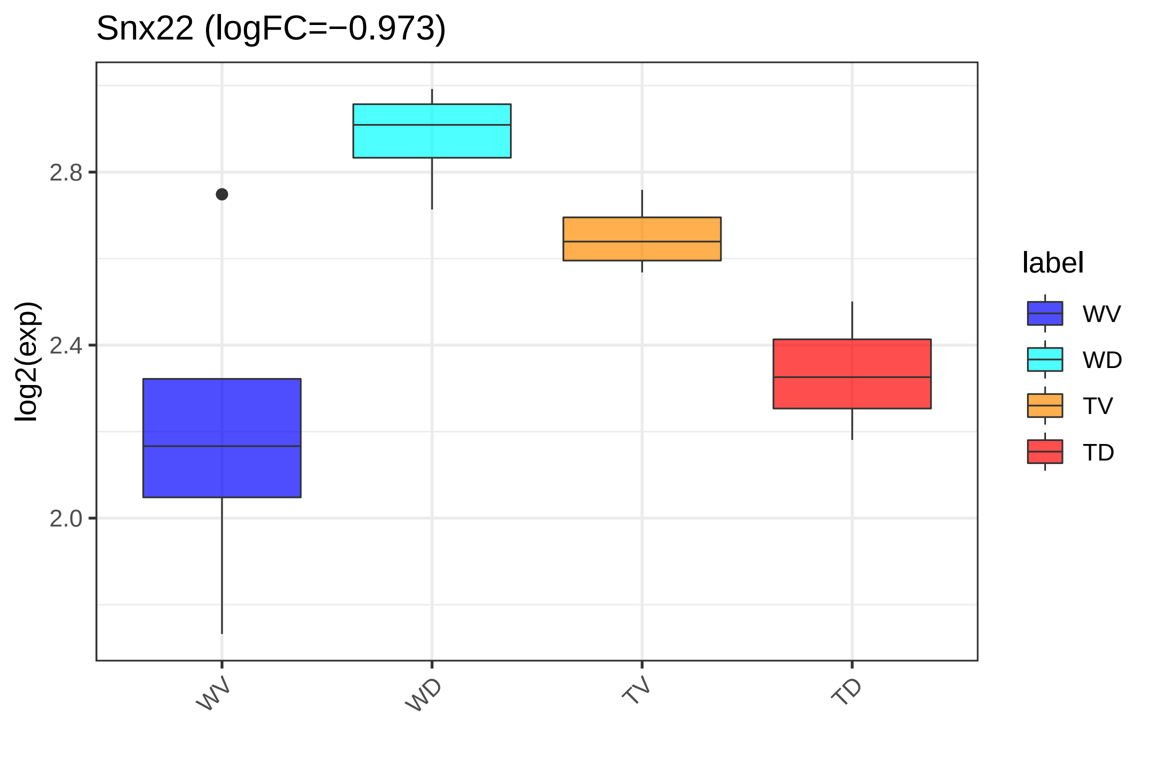

Snx22 (logFC=−0.973)
log2(exp)
2.8
2.4
2.0
WV
WD
TV
TD
label
WV
WD
TV
TD

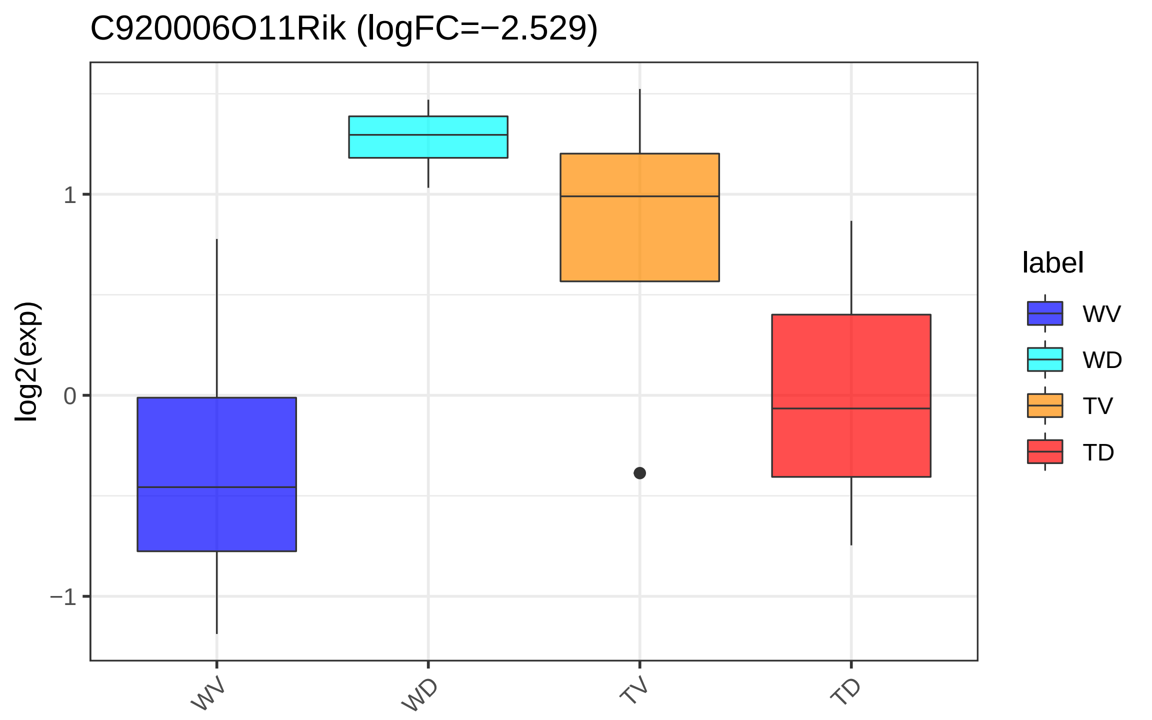
C920006O11Rik (logFC=−2.529)
log2(exp)
1
0
−1
WV
WD
TV
TD
label
WV
WD
TV
TD

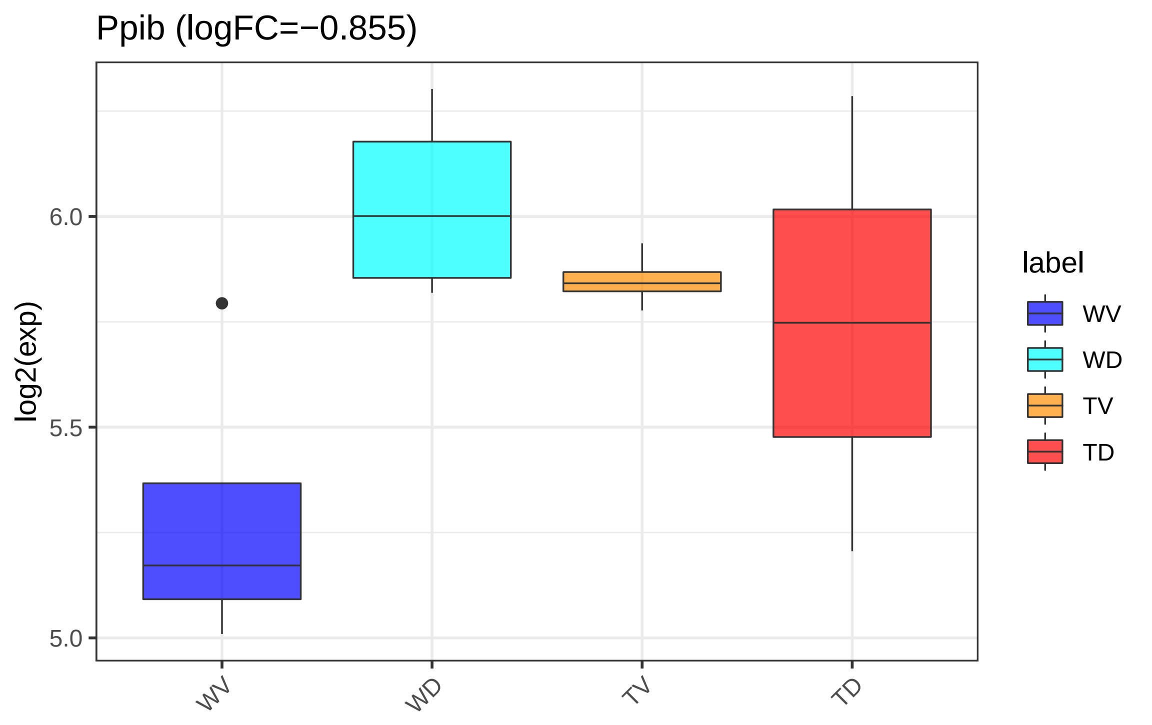

Ppib (logFC=−0.855)
log2(exp)
6.0
5.5
5.0
WV
WD
TV
TD
label
WV
WD
TV
TD

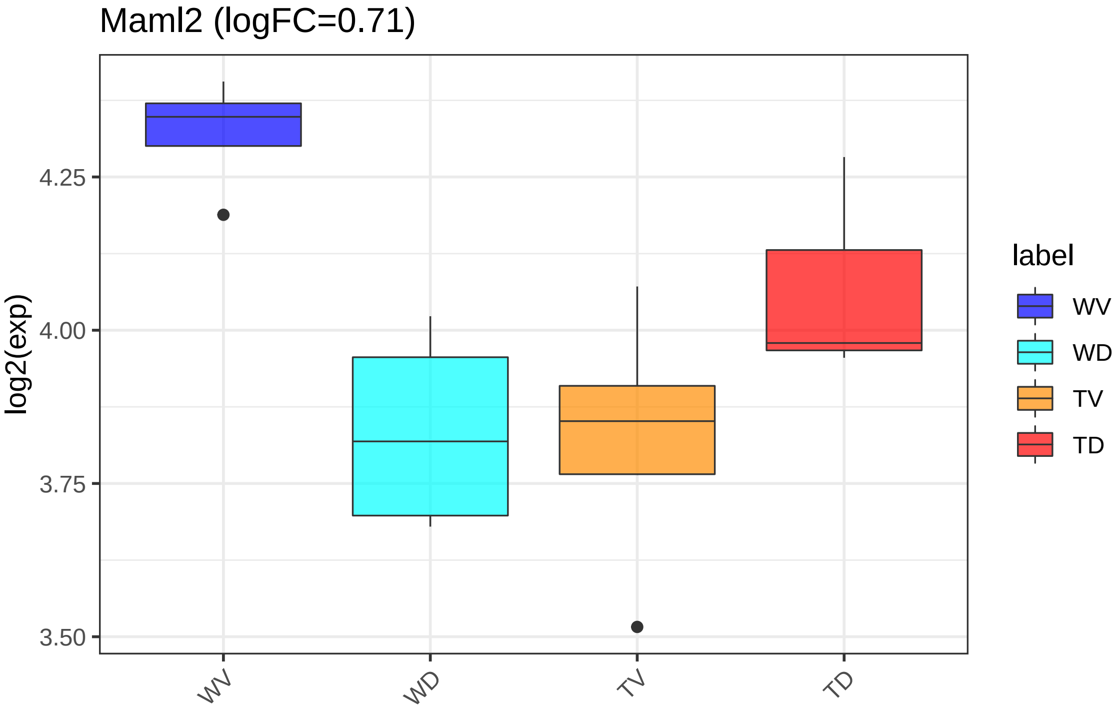

Maml2 (logFC=0.71)
log2(exp)
4.25
4.00
3.75
3.50
WV
WD
TV
TD
label
WV
WD
TV
TD

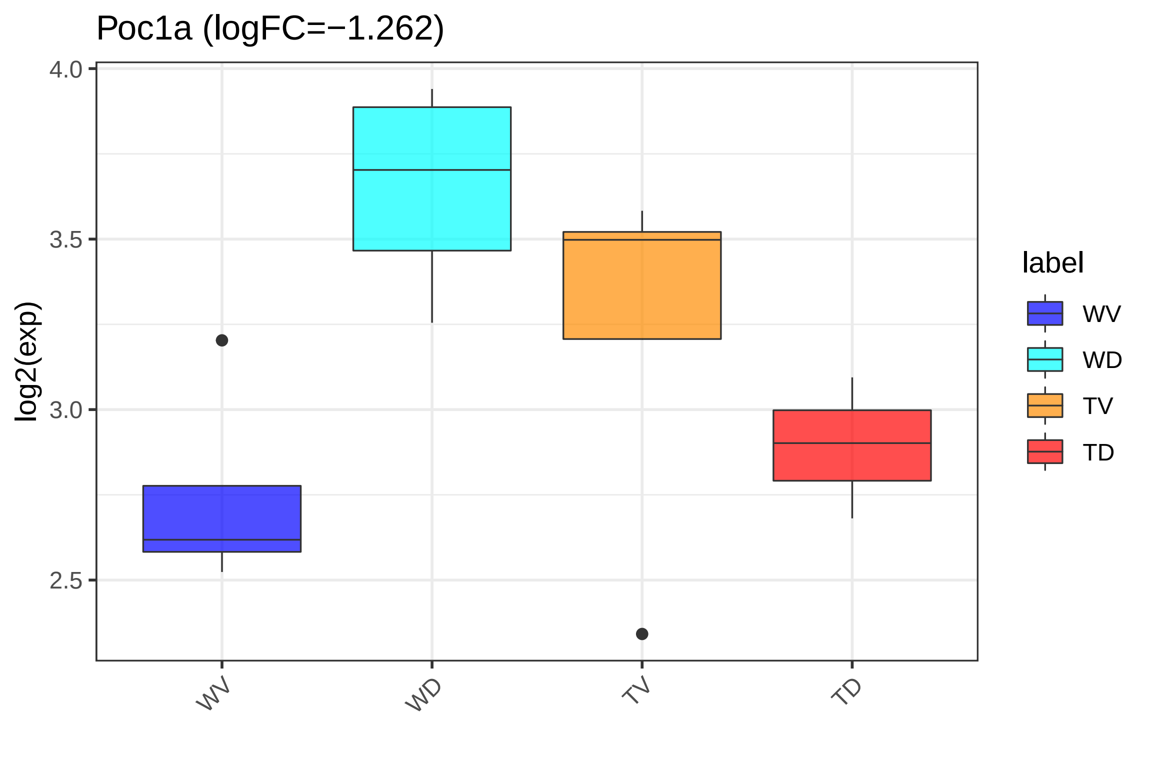

Poc1a (logFC=−1.262)
4.0
3.5
3.0
2.5
log2(exp)
WV
WD
TV
TD
label
WV
WD
TV
TD

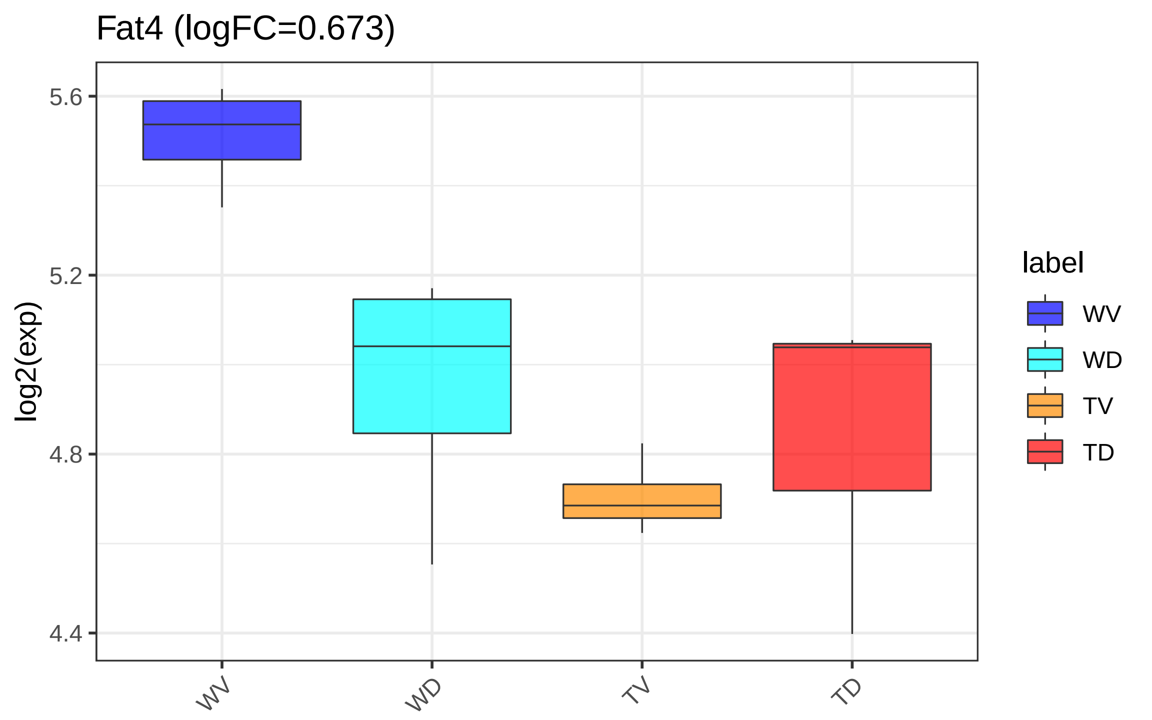
Fat4 (logFC=0.673)
log2(exp)
5.6
5.2
4.8
4.4
WV
WD
TV
TD
label
WV
WD
TV
TD

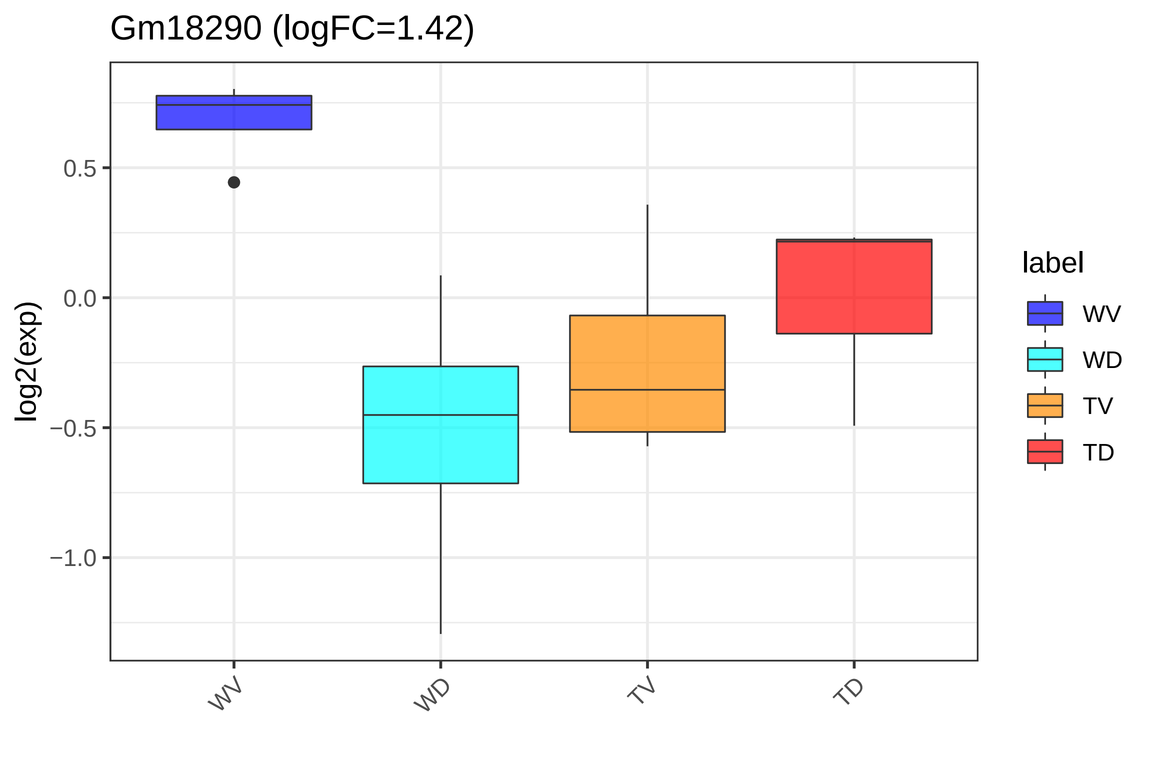

Gm18290 (logFC=1.42)
log2(exp)
0.5
0.0
−0.5
−1.0
WV
WD
TV
TD
label
WV
WD
TV
TD

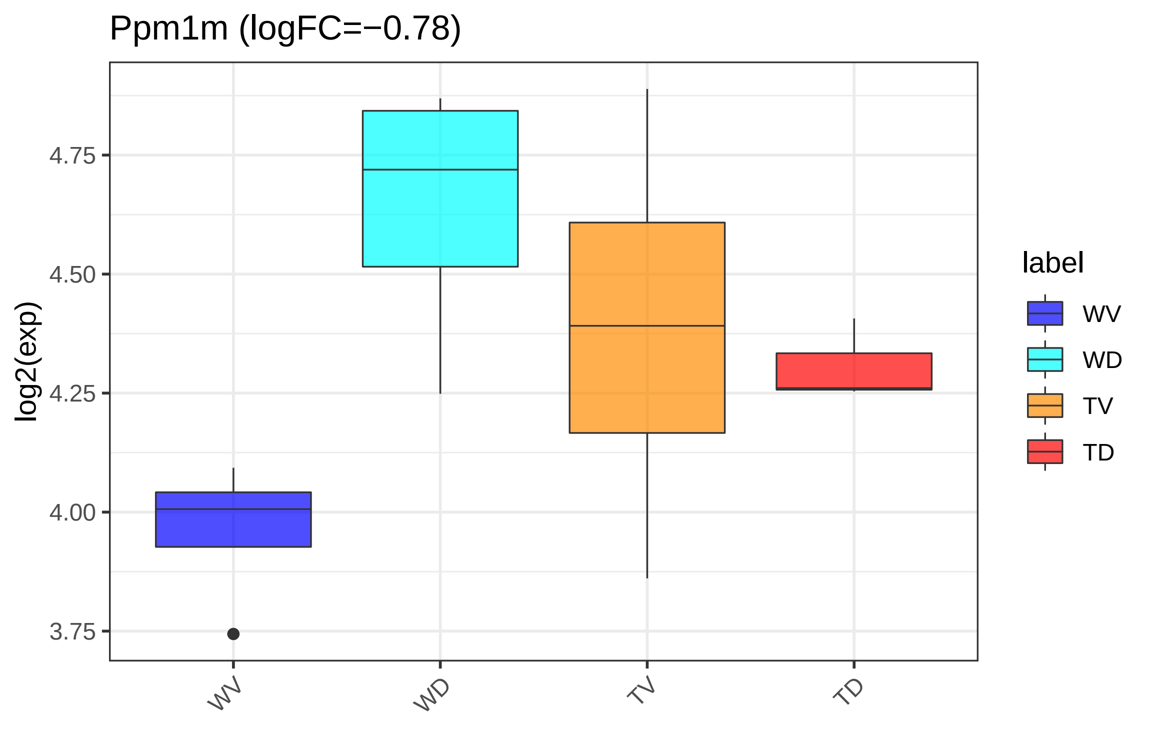
Ppm1m (logFC=−0.78)
log2(exp)
4.75
4.50
4.25
4.00
3.75
WV
WD
TV
TD
label
WV
WD
TV
TD

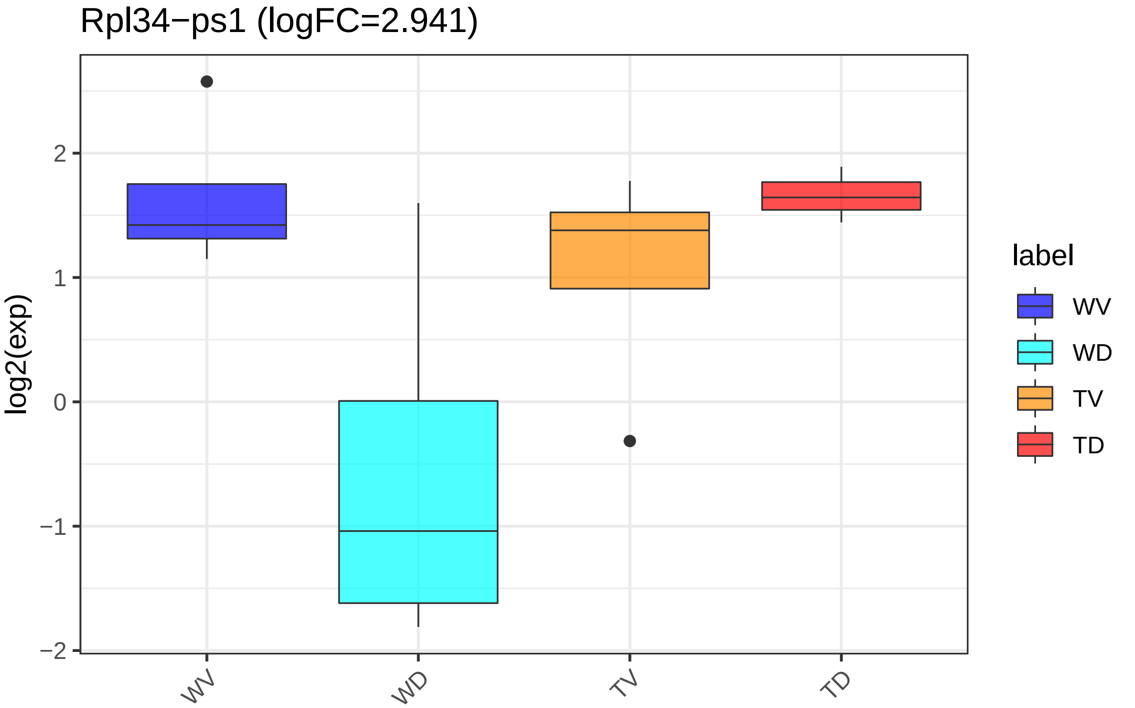

Rpl34−ps1 (logFC=2.941)
log2(exp)
2
1
0
−1
−2
WV
WD
TV
TD
label
WV
WD
TV
TD

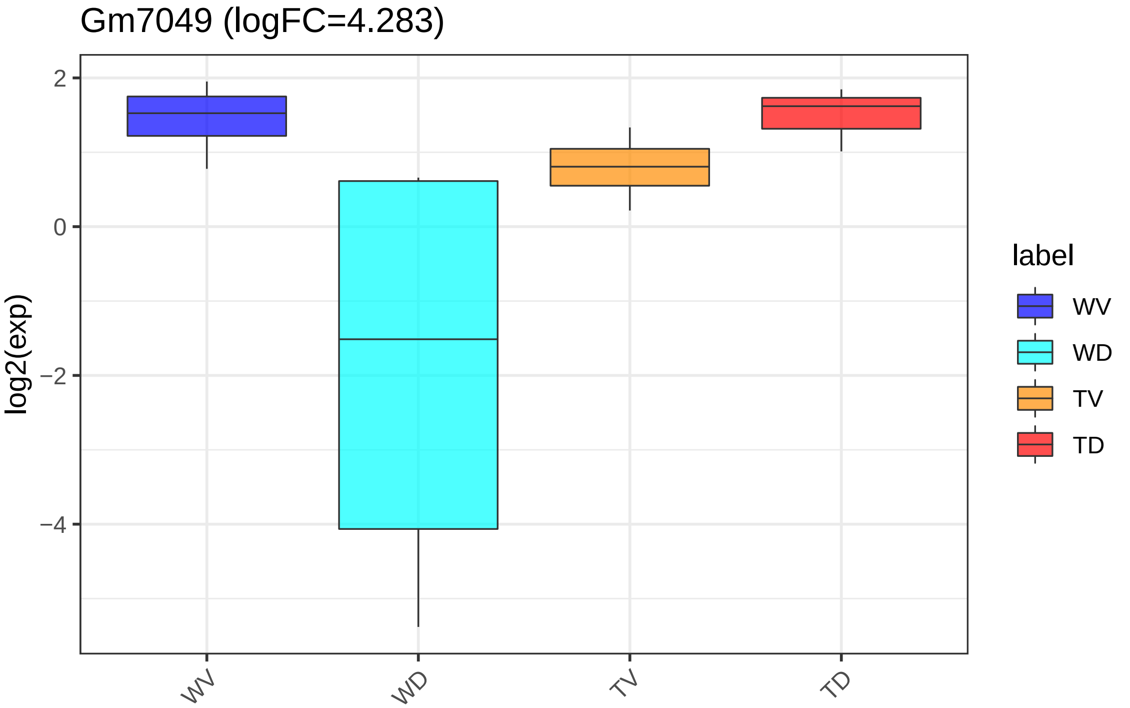

Gm7049 (logFC=4.283)
log2(exp)
2
0
−2
−4
WV
WD
TV
TD
label
WV
WD
TV
TD

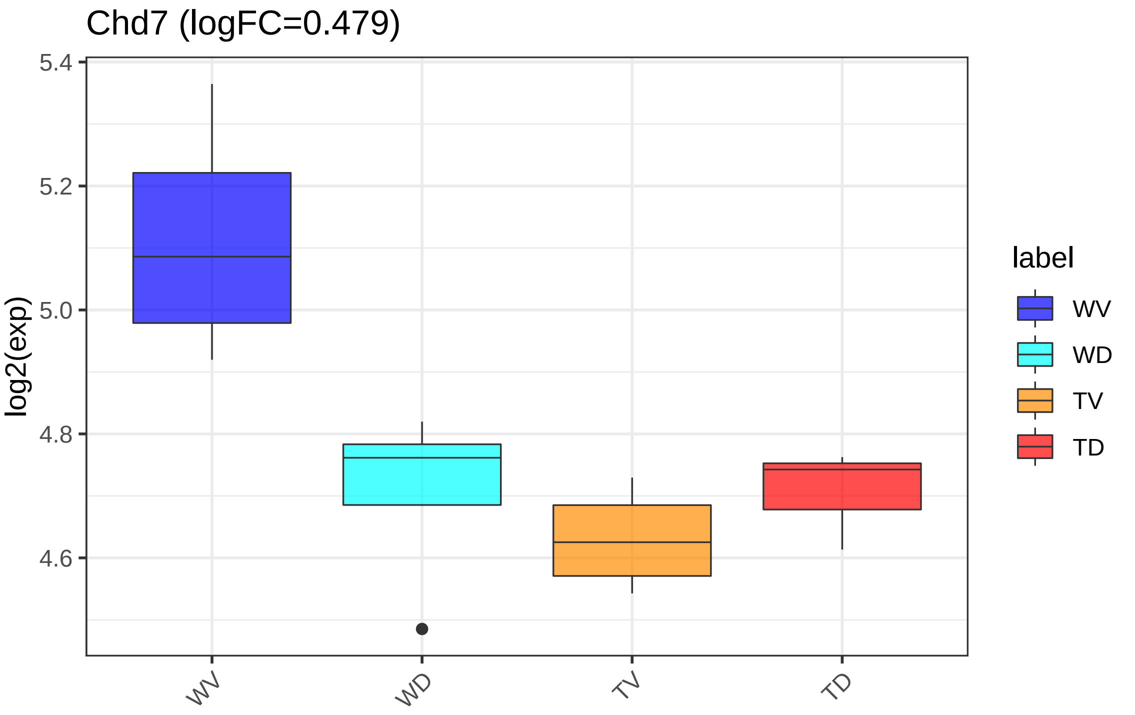

Chd7 (logFC=0.479)
log2(exp)
5.4
5.2
5.0
4.8
4.6
WV
WD
TV
TD
label
WV
WD
TV
TD

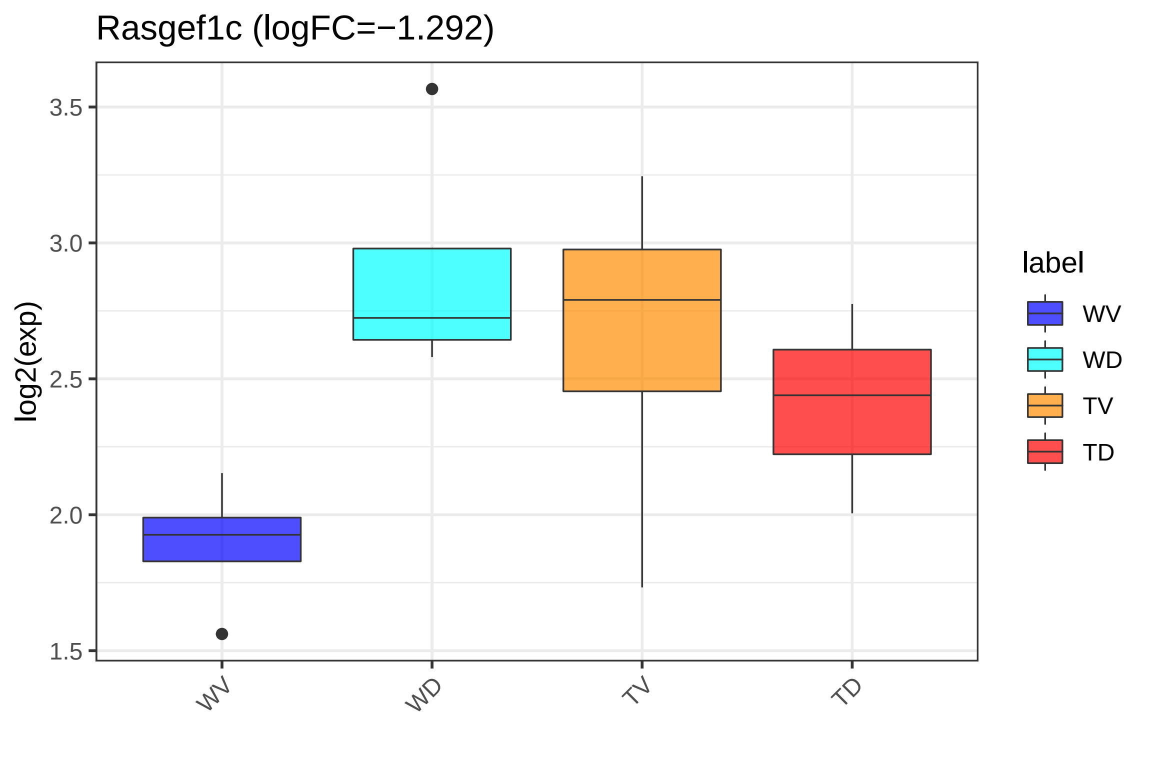
Rasgef1c (logFC=−1.292)
log2(exp)
3.5
3.0
2.5
2.0
1.5
WV
WD
TV
TD
label
WV
WD
TV
TD

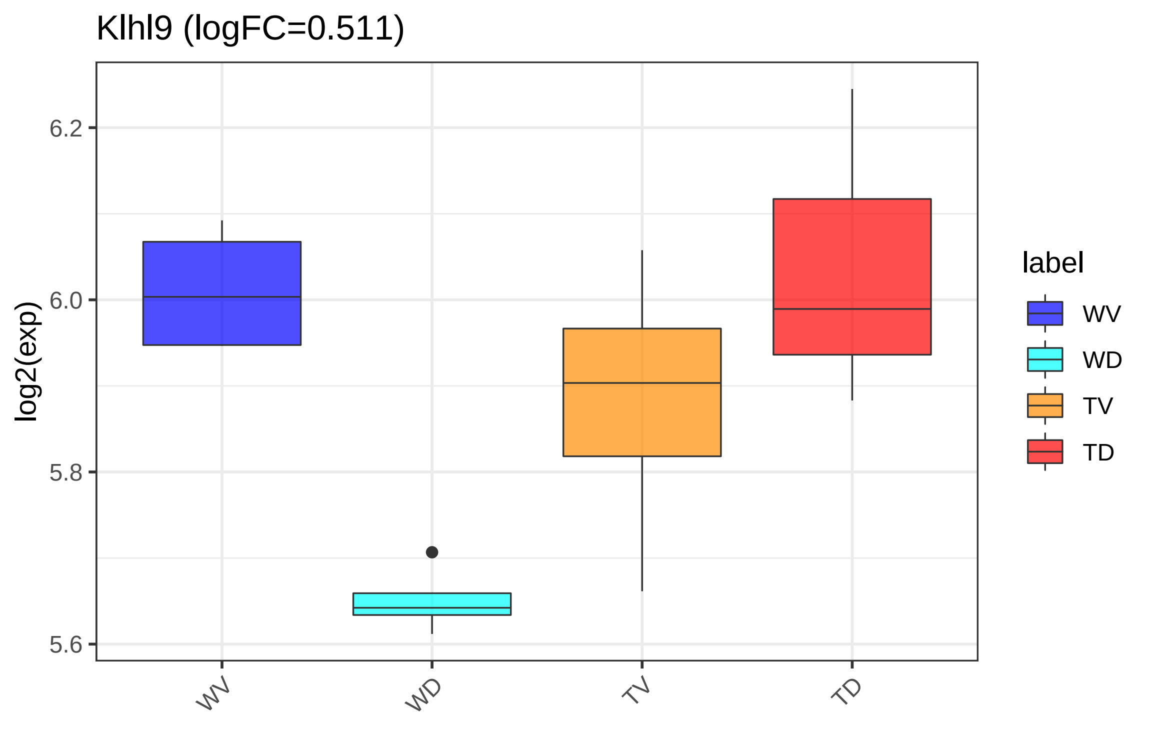
Klhl9 (logFC=0.511)
log2(exp)
6.2
6.0
5.8
5.6
WV
WD
TV
TD
label
WV
WD
TV
TD

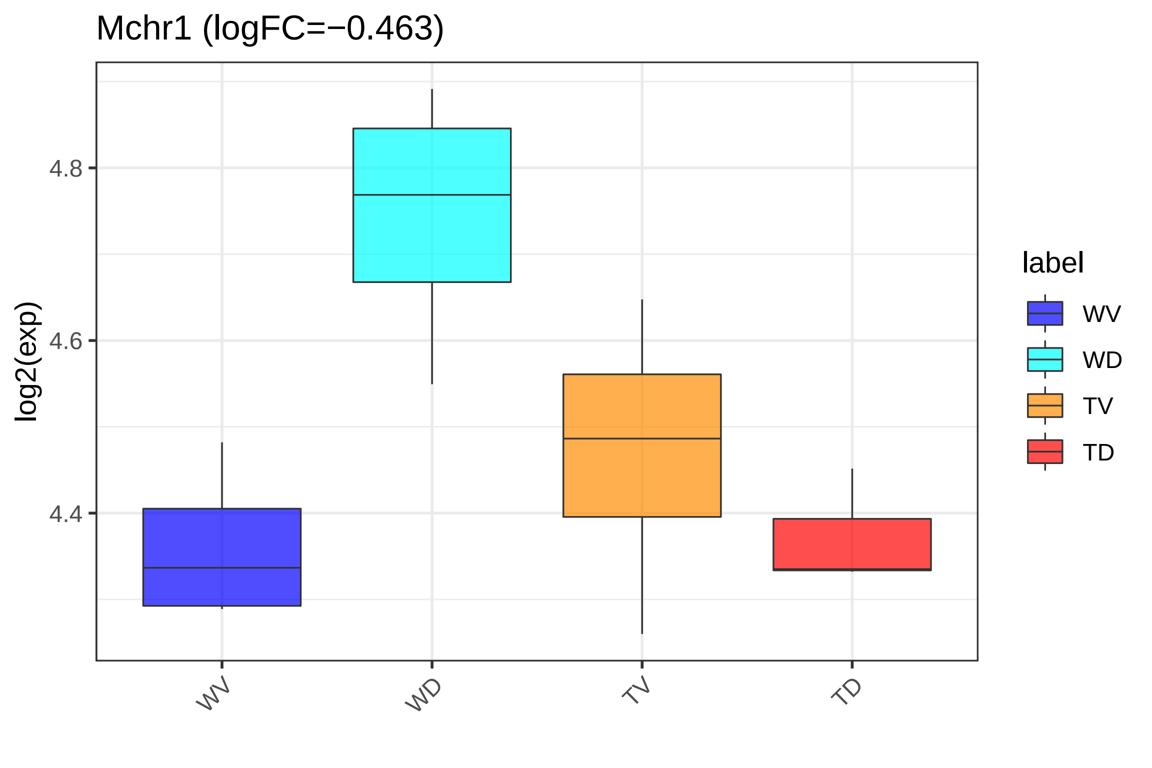
Mchr1 (logFC=−0.463)
log2(exp)
4.8
4.6
4.4
WV
WD
TV
TD
label
WV
WD
TV
TD

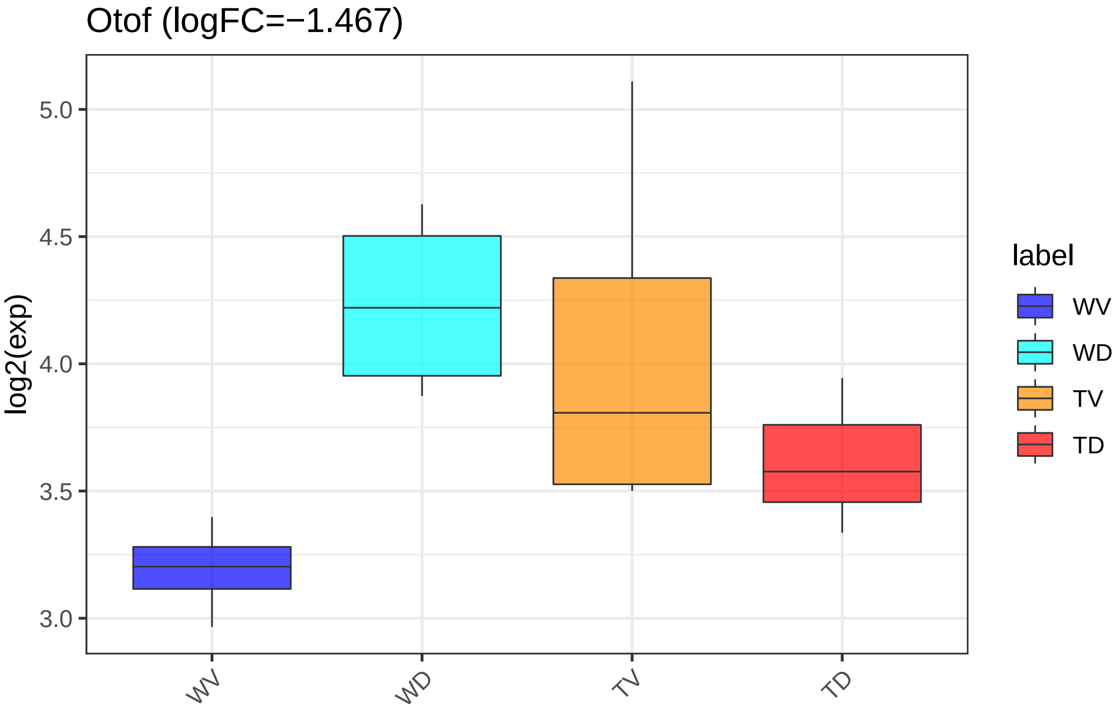
Otof (logFC=−1.467)
log2(exp)
5.0
4.5
4.0
3.5
3.0
WV
WD
TV
TD
label
WV
WD
TV
TD

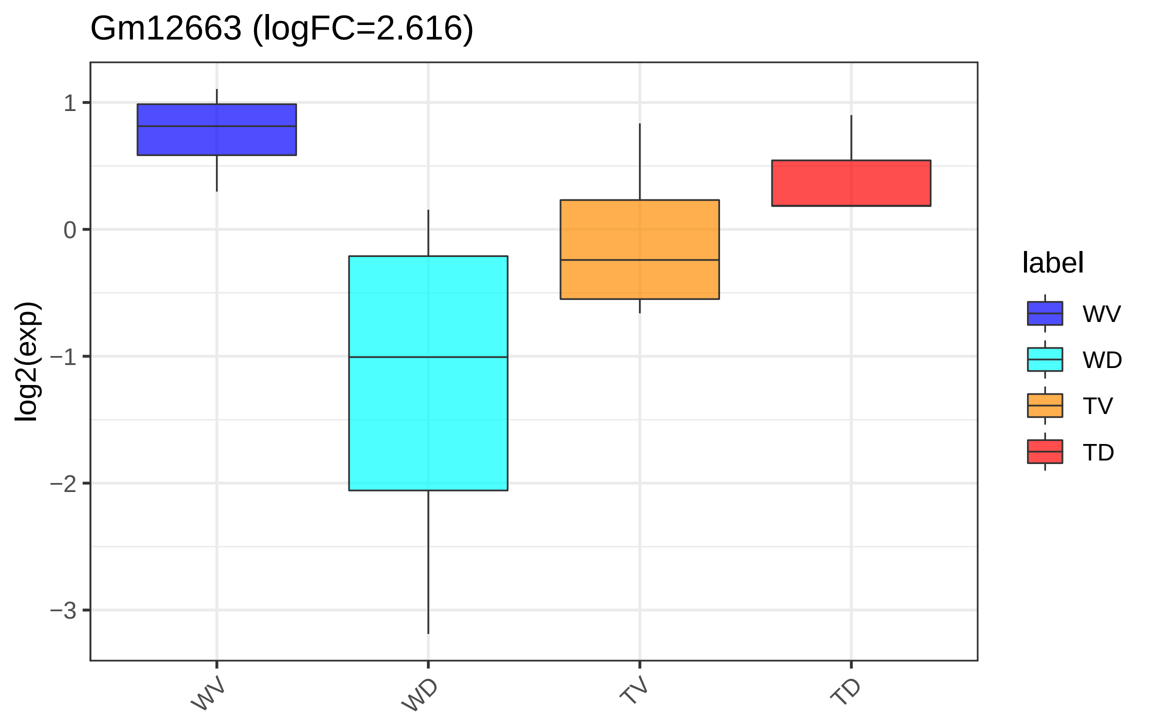

Gm12663 (logFC=2.616)
log2(exp)
1
0
−1
−2
−3
WV
WD
TV
TD
label
WV
WD
TV
TD

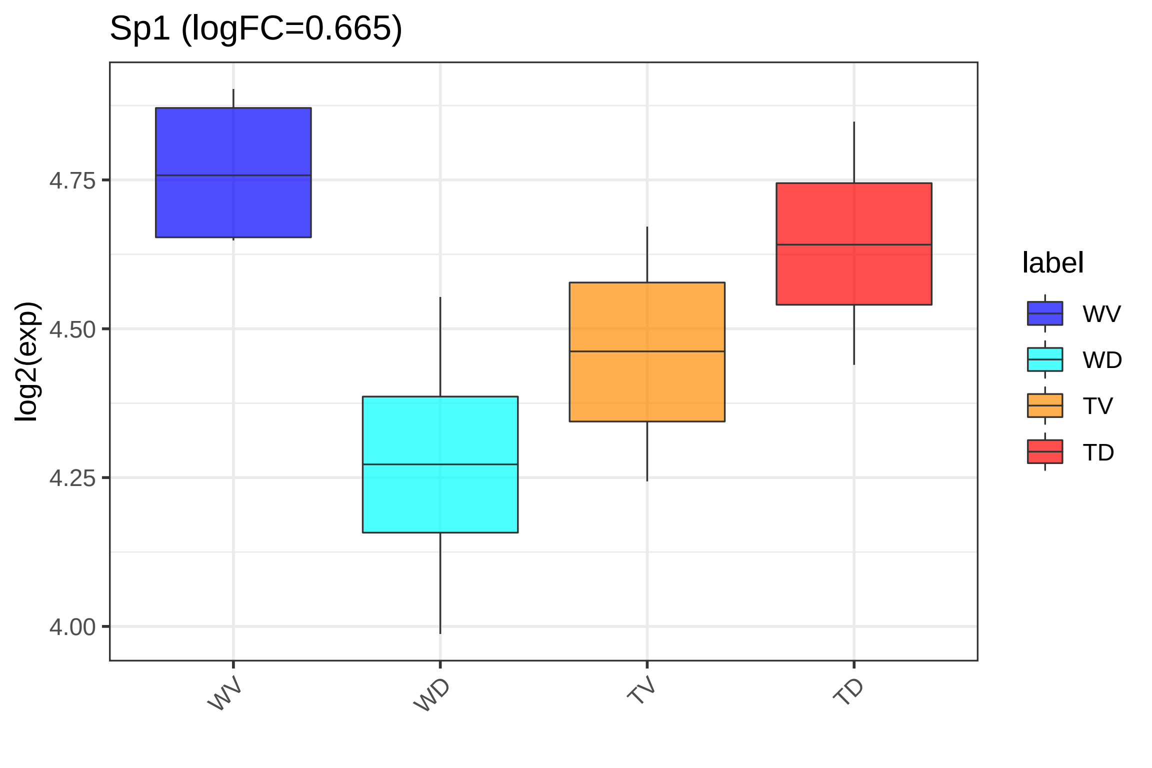

Sp1 (logFC=0.665)
log2(exp)
4.75
4.50
4.25
4.00
WV
WD
TV
TD
label
WV
WD
TV
TD

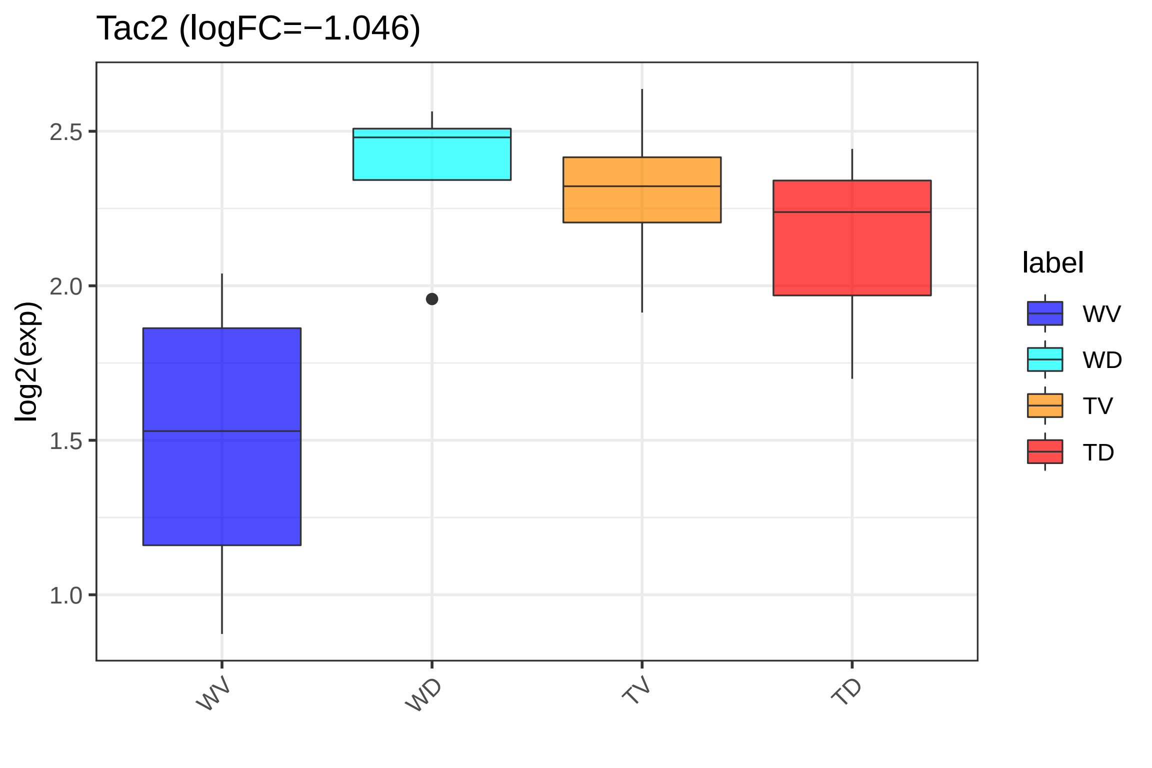
Tac2 (logFC=−1.046)
log2(exp)
2.5
2.0
1.5
1.0
WV
WD
TV
TD
label
WV
WD
TV
TD

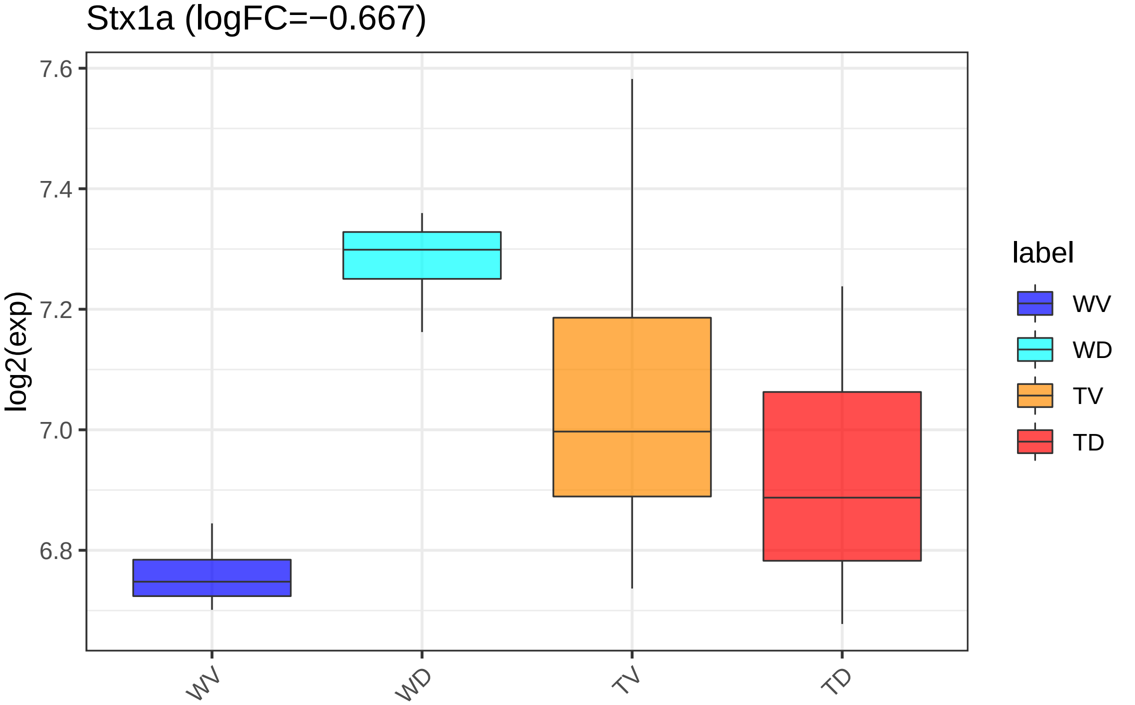
Stx1a (logFC=−0.667)
log2(exp)
7.6
7.4
7.2
7.0
6.8
WV
WD
TV
TD
label
WV
WD
TV
TD

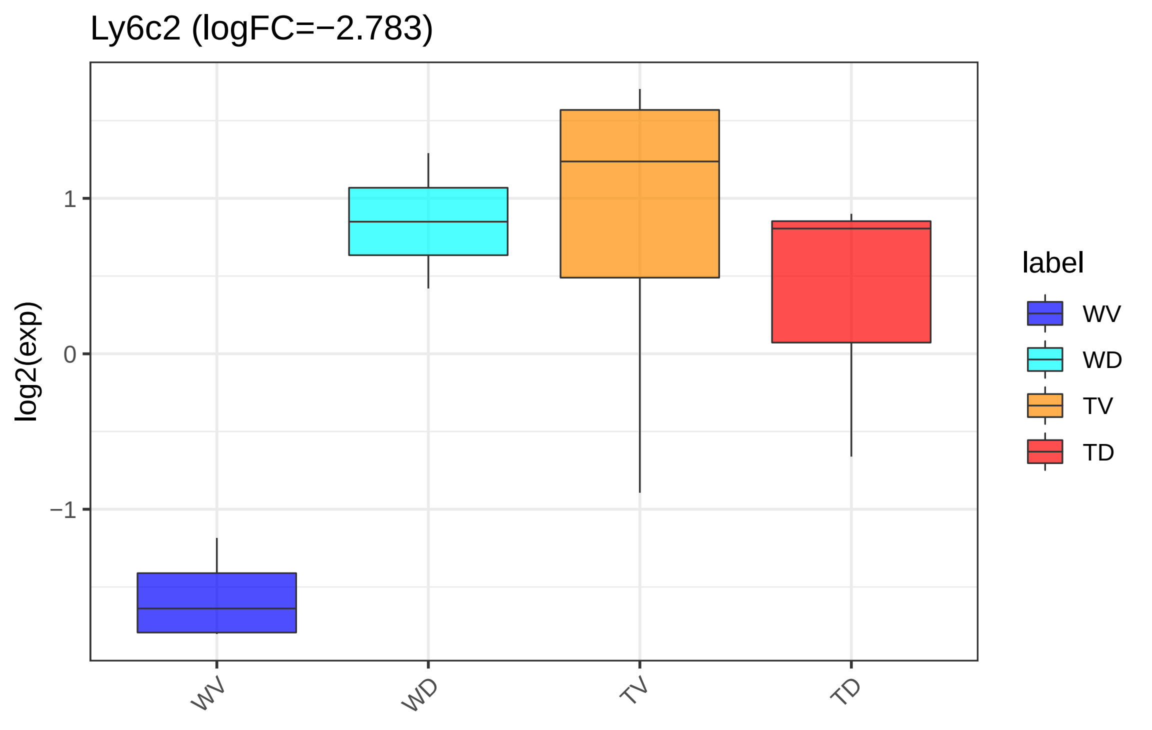

Ly6c2 (logFC=−2.783)
log2(exp)
1
0
−1
WV
WD
TV
TD
label
WV
WD
TV
TD

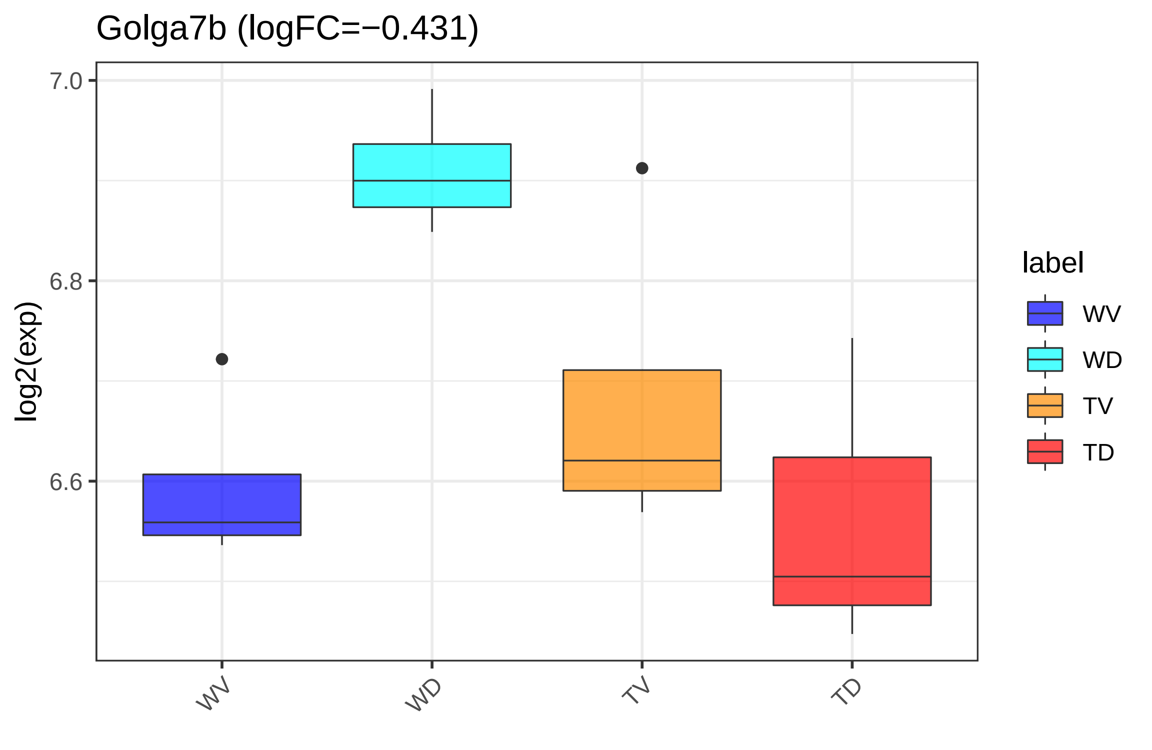

Golga7b (logFC=−0.431)
7.0
6.8
6.6
log2(exp)
WV
WD
TV
TD
label
WV
WD
TV
TD

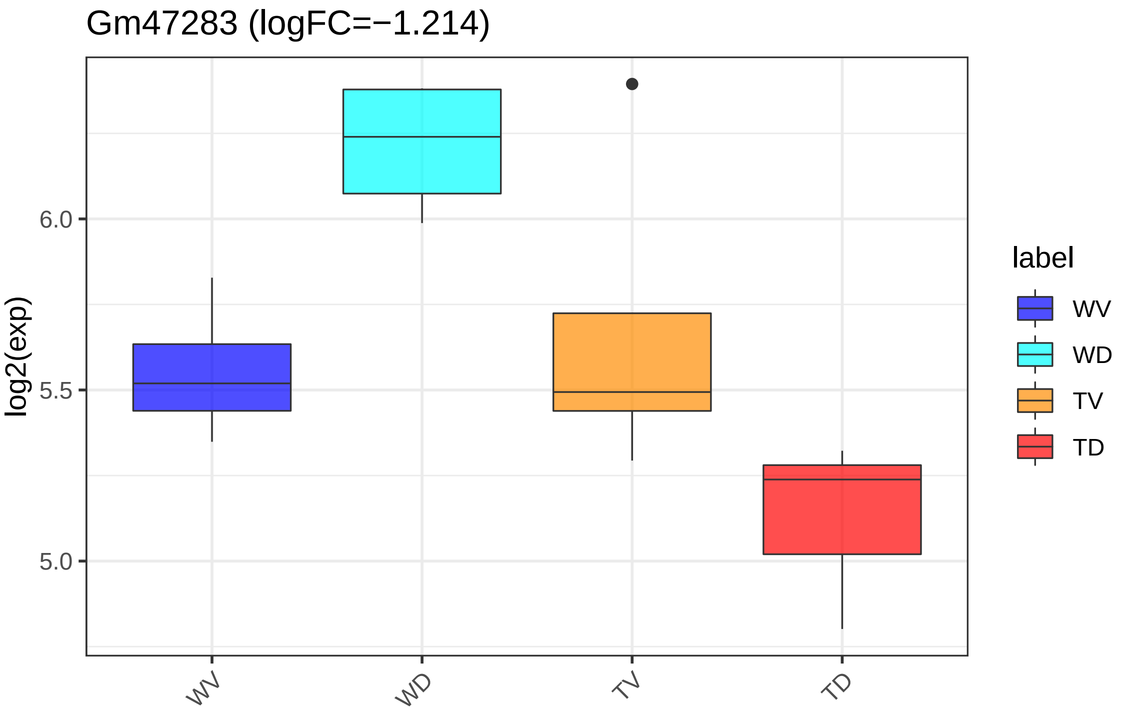

Gm47283 (logFC=−1.214)
log2(exp)
6.0
5.5
5.0
WV
WD
TV
TD
label
WV
WD
TV
TD

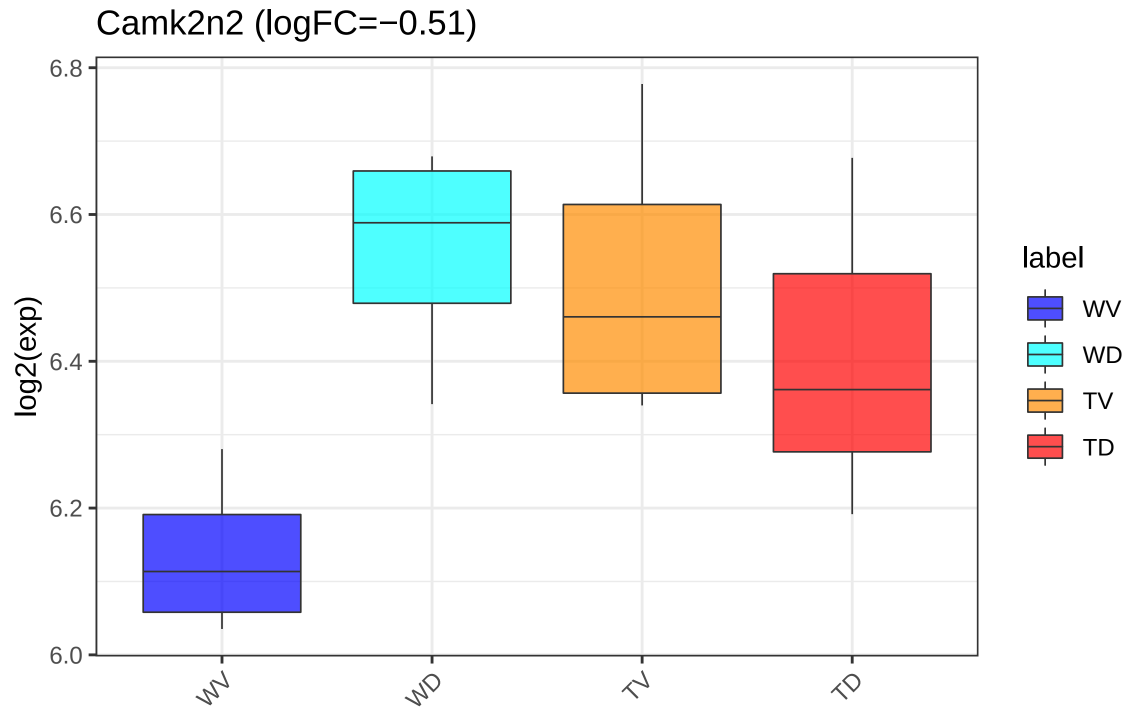
Camk2n2 (logFC=−0.51)
log2(exp)
6.8
6.6
6.4
6.2
6.0
WV
WD
TV
TD
label
WV
WD
TV
TD

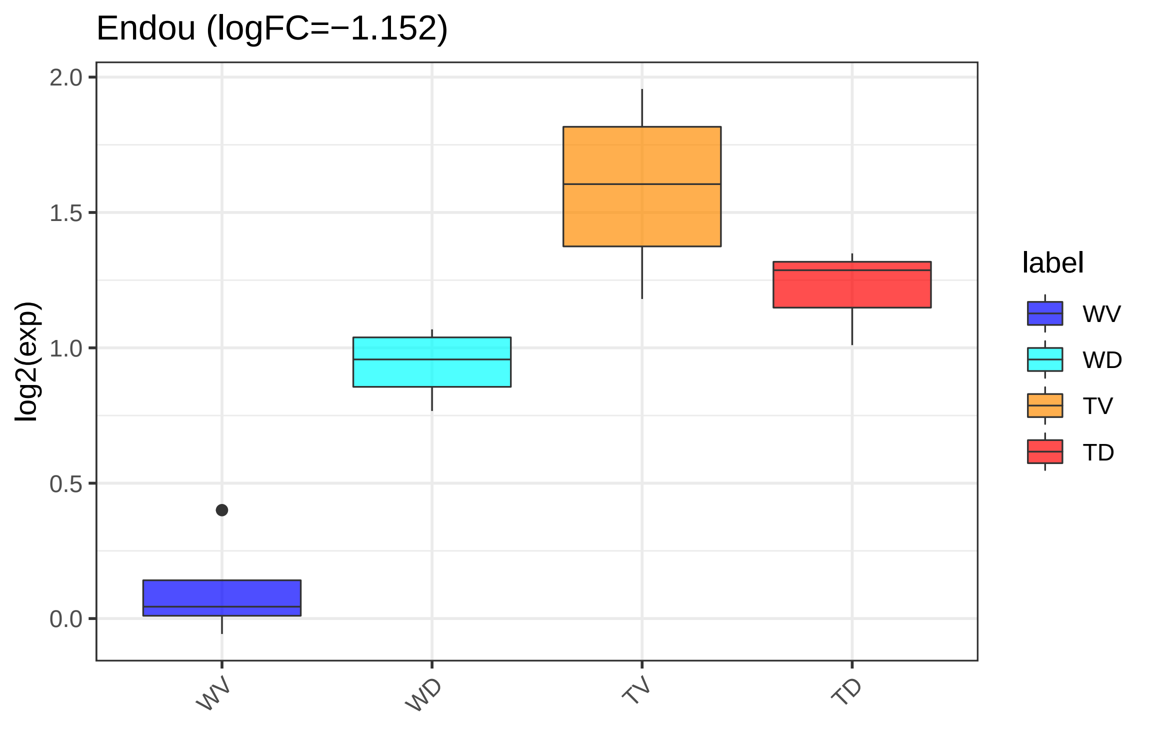

Endou (logFC=−1.152)
log2(exp)
2.0
1.5
1.0
0.5
0.0
WV
WD
TV
TD
label
WV
WD
TV
TD

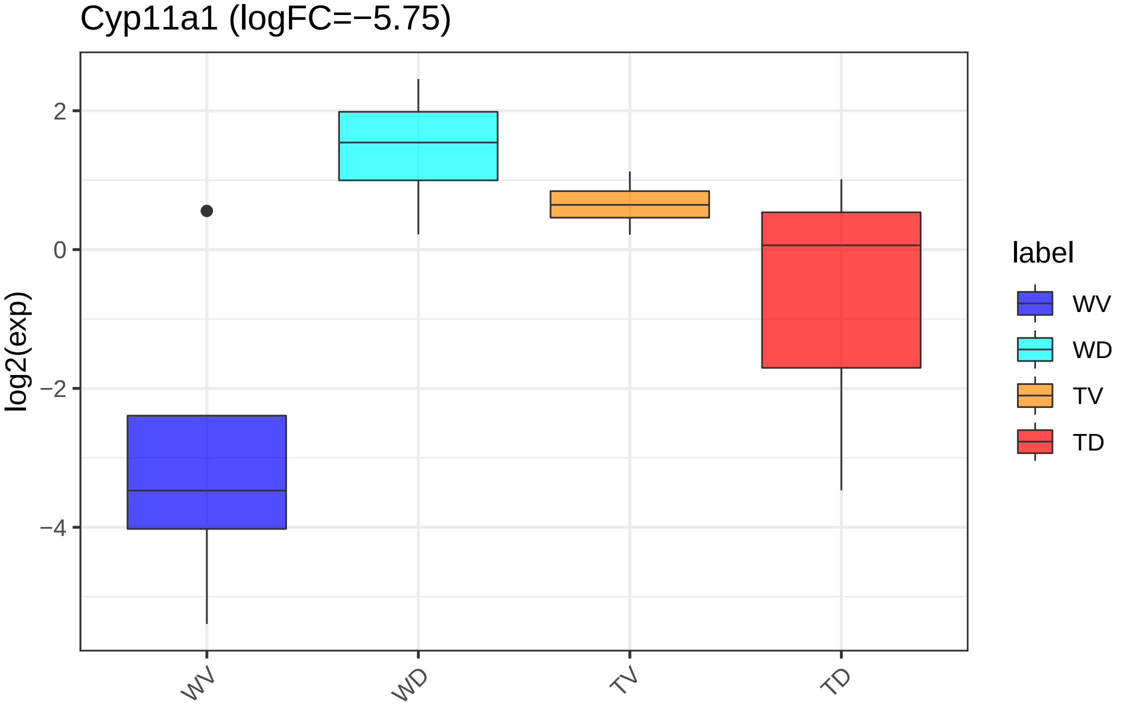
Cyp11a1 (logFC=−5.75)
log2(exp)
2
0
−2
−4
WV
WD
TV
TD
label
WV
WD
TV
TD

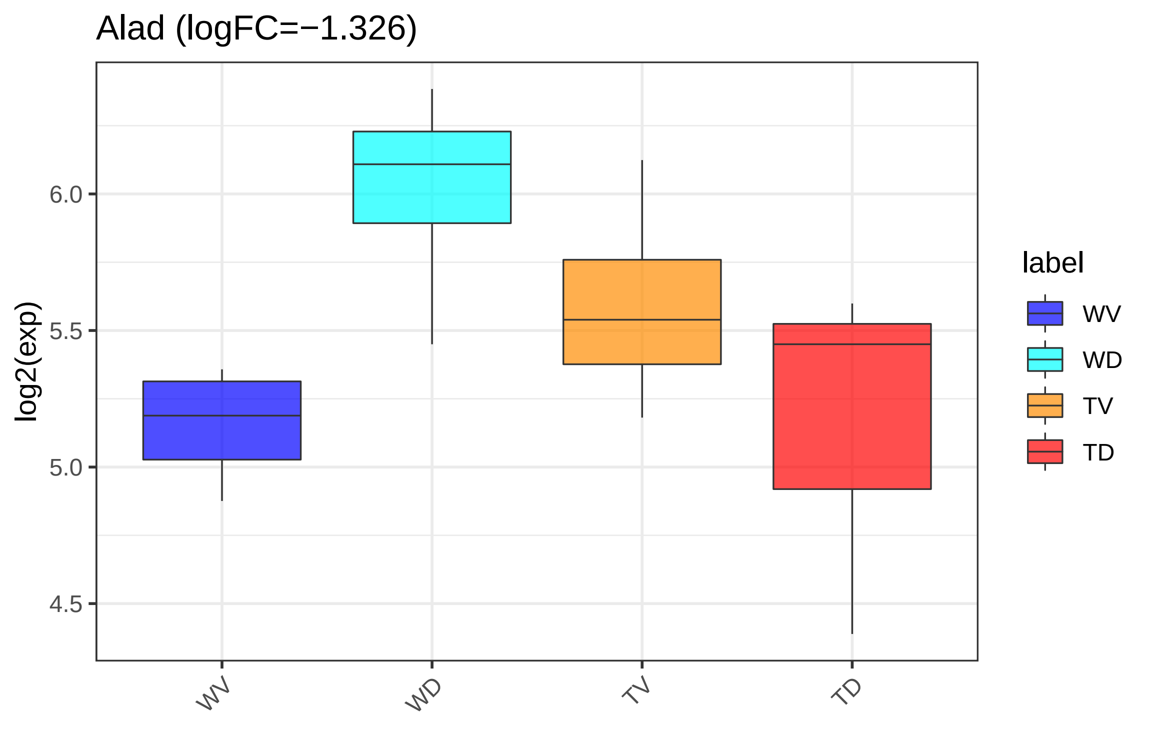
Alad (logFC=−1.326)
log2(exp)
6.0
5.5
5.0
4.5
WV
WD
TV
TD
label
WV
WD
TV
TD

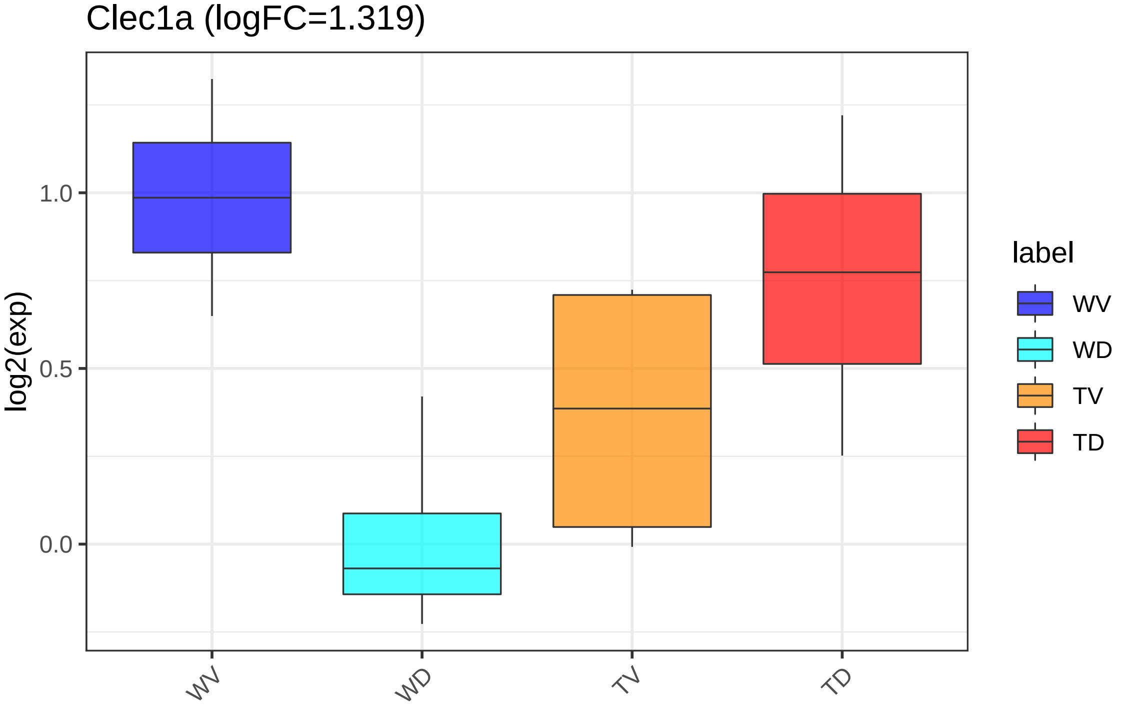
Clec1a (logFC=1.319)
log2(exp)
1.0
0.5
0.0
WV
WD
TV
TD
label
WV
WD
TV
TD

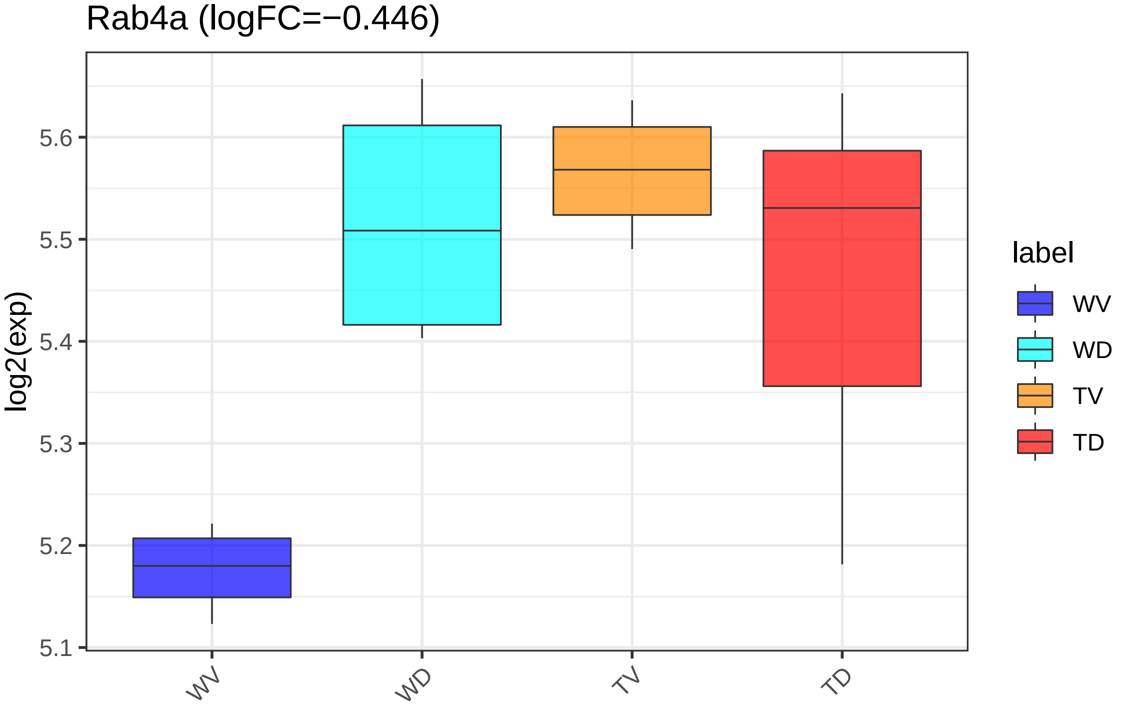

Rab4a (logFC=−0.446)
log2(exp)
5.1
5.2
5.3
5.4
5.5
5.6
WV
WD
TV
TD
label
WV
WD
TV
TD

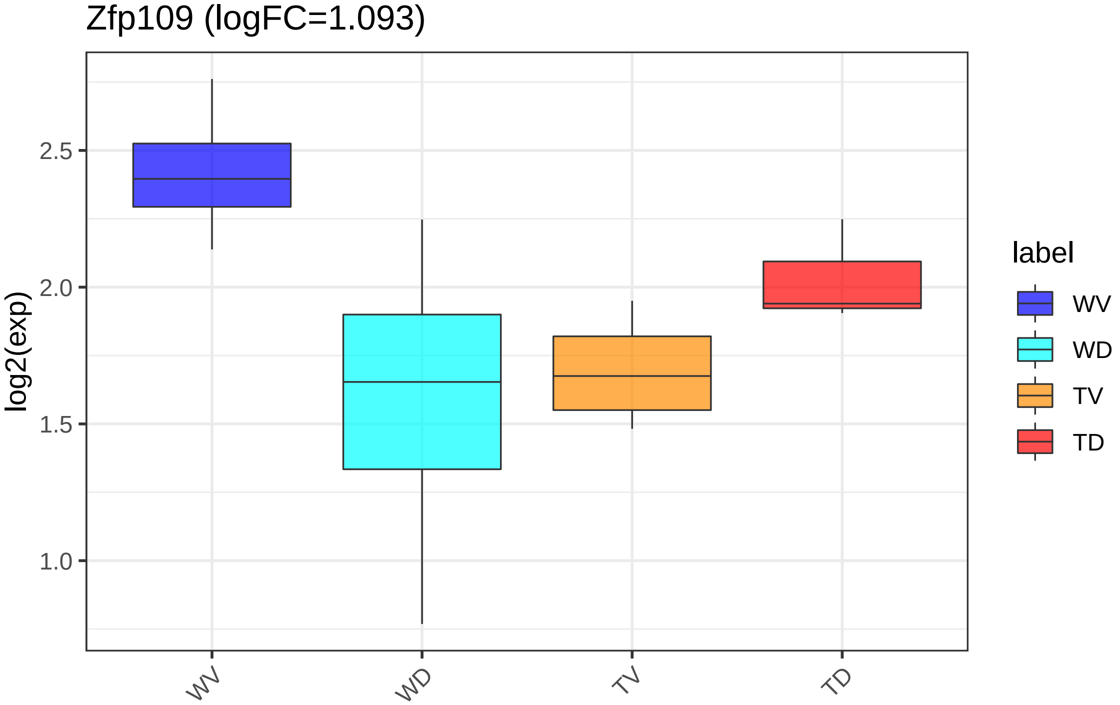
Zfp109 (logFC=1.093)
log2(exp)
2.5
2.0
1.5
1.0
WV
WD
TV
TD
label
WV
WD
TV
TD

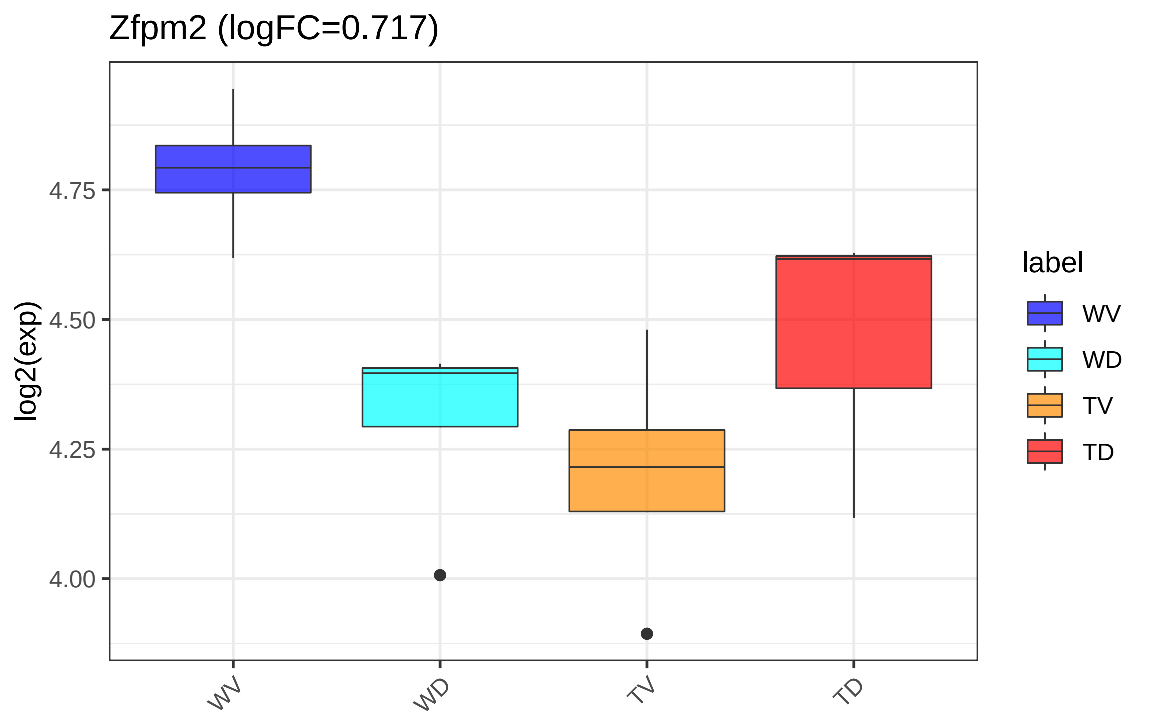
Zfpm2 (logFC=0.717)
log2(exp)
4.75
4.50
4.25
4.00
WV
WD
TV
TD
label
WV
WD
TV
TD

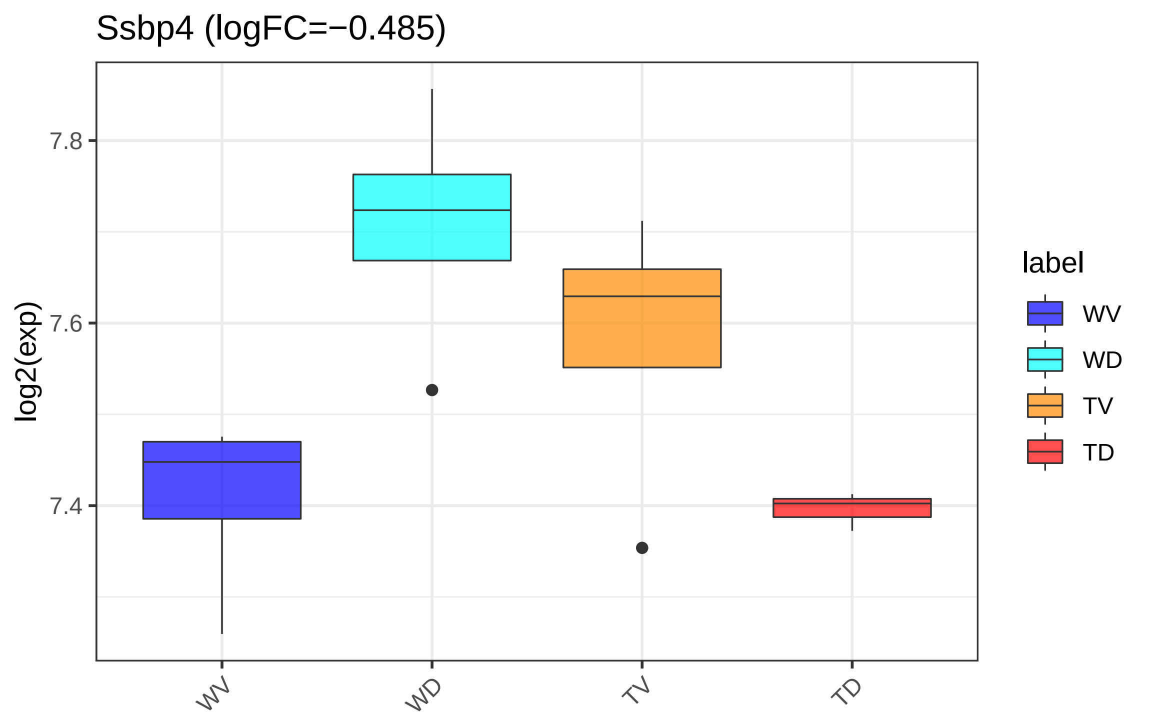
Ssbp4 (logFC=−0.485)
log2(exp)
7.8
7.6
7.4
WV
WD
TV
TD
label
WV
WD
TV
TD

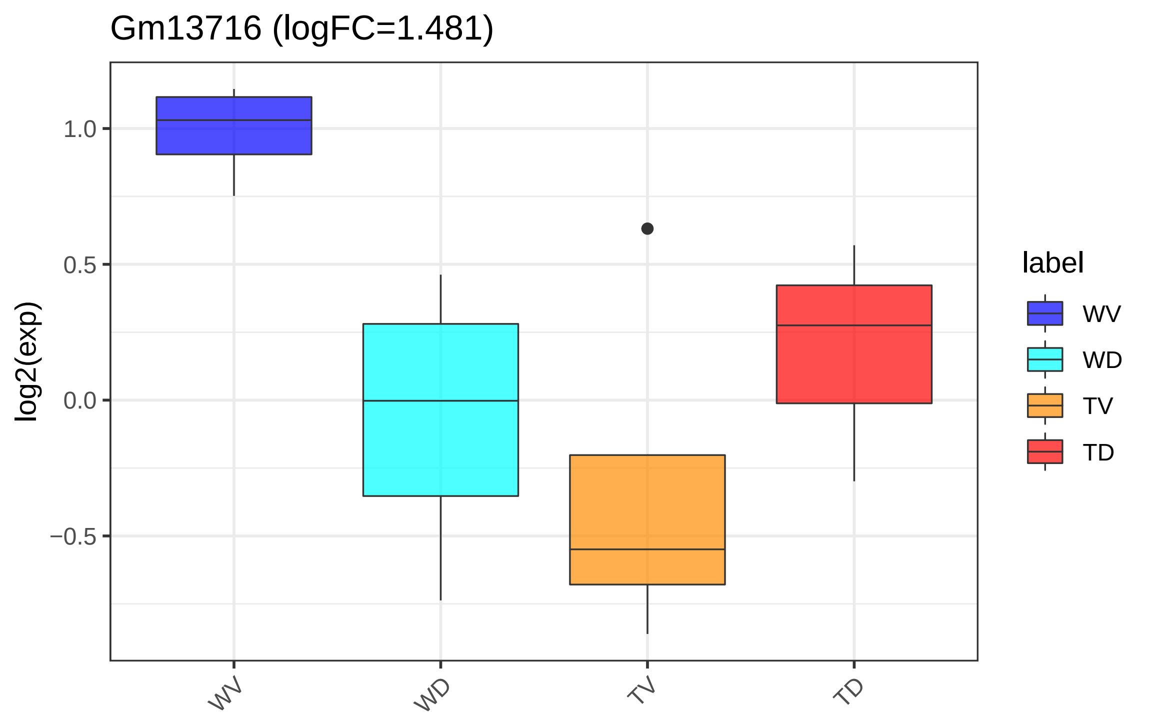

Gm13716 (logFC=1.481)
log2(exp)
1.0
0.5
0.0
−0.5
WV
WD
TV
TD
label
WV
WD
TV
TD

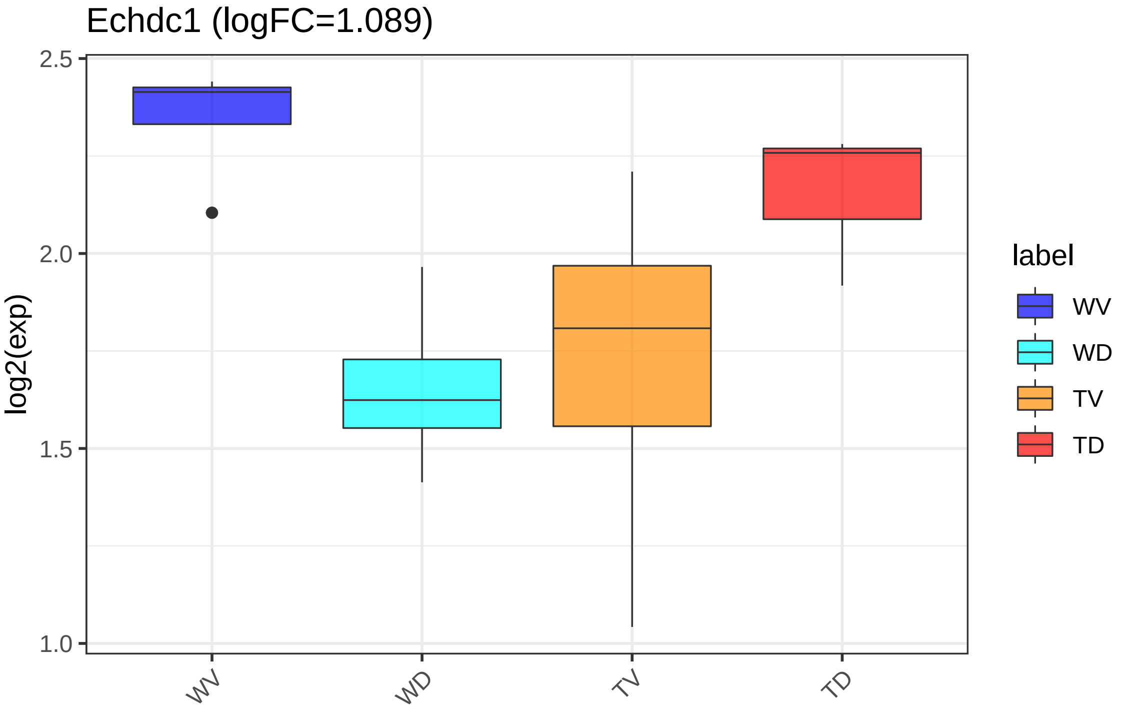
Echdc1 (logFC=1.089)
2.5
2.0
1.5
1.0
log2(exp)
WV
WD
TV
TD
label
WV
WD
TV
TD

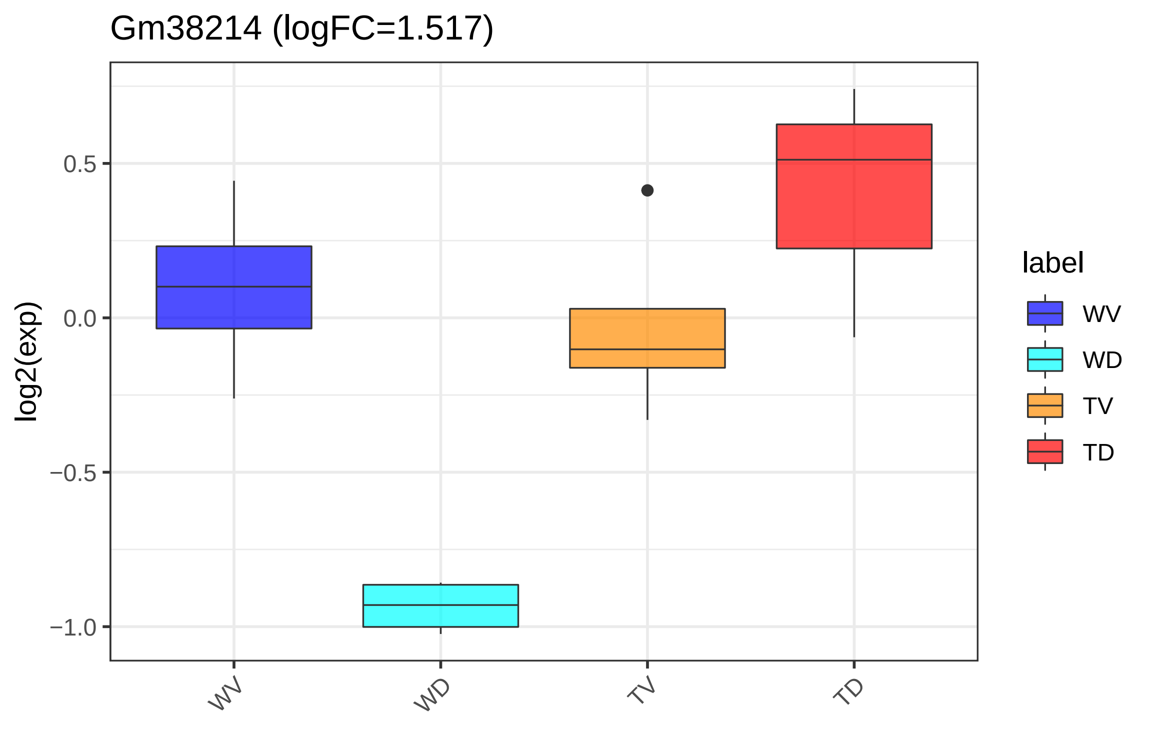

Gm38214 (logFC=1.517)
log2(exp)
0.5
0.0
−0.5
−1.0
WV
WD
TV
TD
label
WV
WD
TV
TD

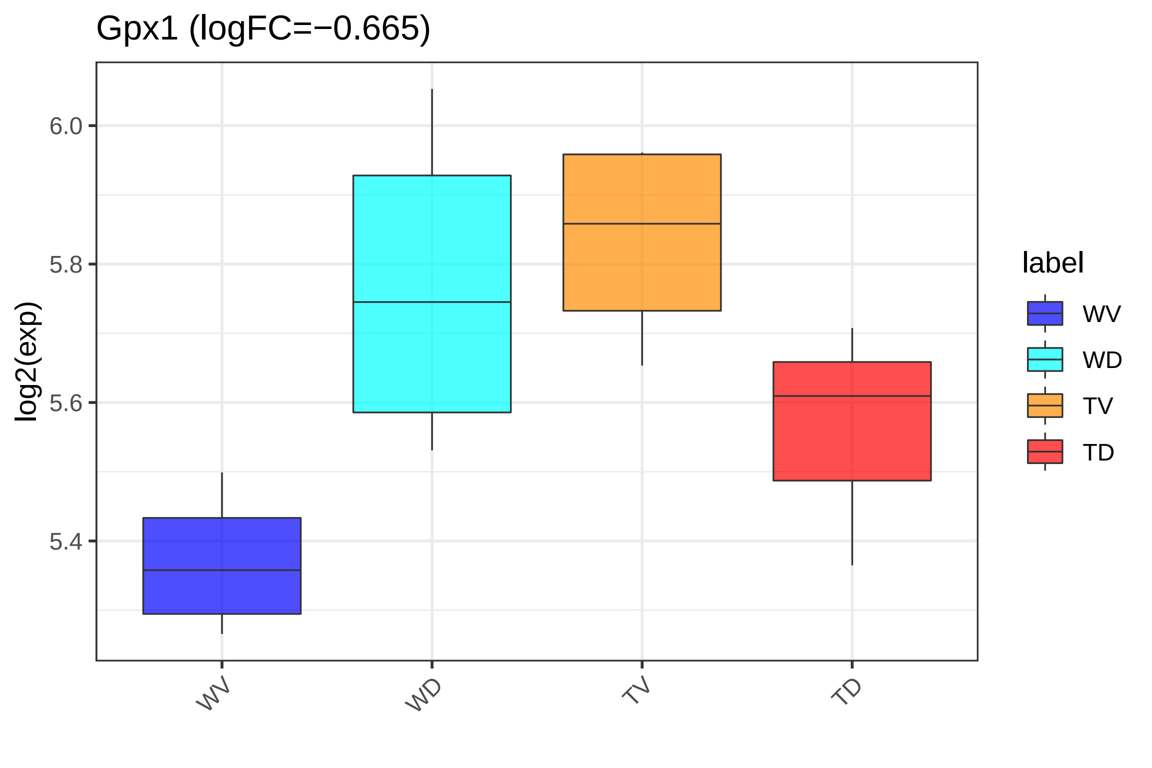

Gpx1 (logFC=−0.665)
log2(exp)
6.0
5.8
5.6
5.4
WV
WD
TV
TD
label
WV
WD
TV
TD

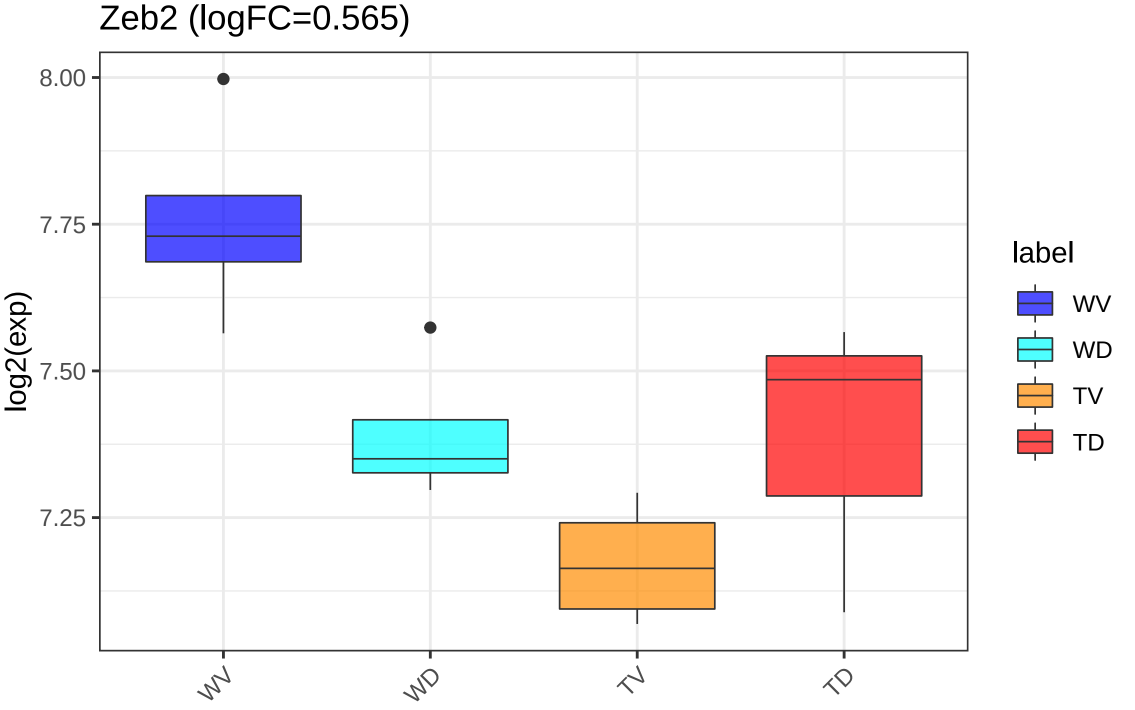

Zeb2 (logFC=0.565)
log2(exp)
8.00
7.75
7.50
7.25
WV
WD
TV
TD
label
WV
WD
TV
TD

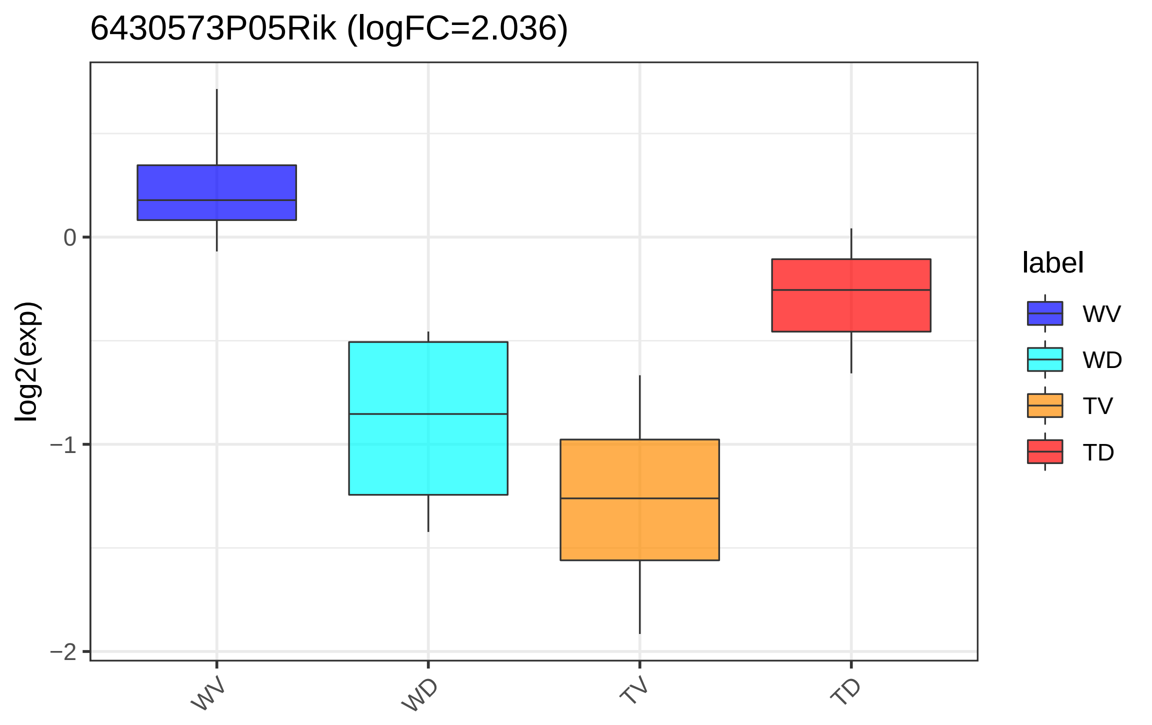
6430573P05Rik (logFC=2.036)
log2(exp)
0
−1
−2
WV
WD
TV
TD
label
WV
WD
TV
TD

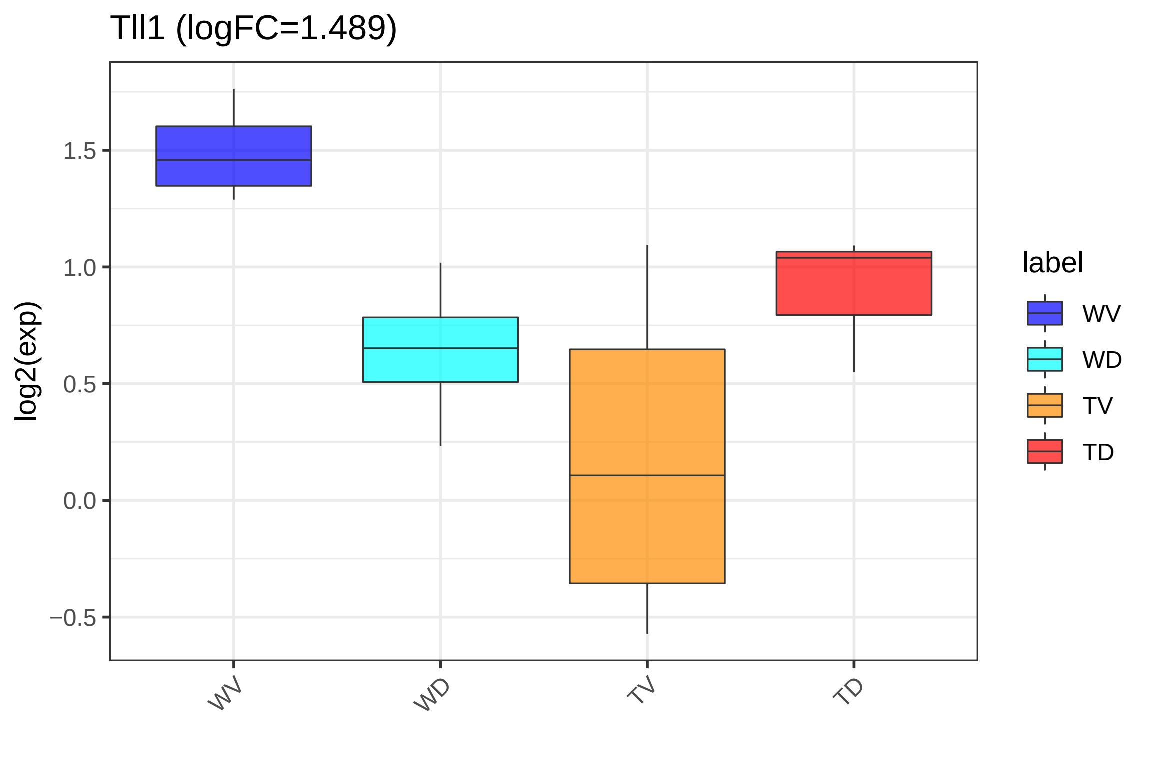

Tll1 (logFC=1.489)
log2(exp)
1.5
1.0
0.5
0.0
−0.5
WV
WD
TV
TD
label
WV
WD
TV
TD

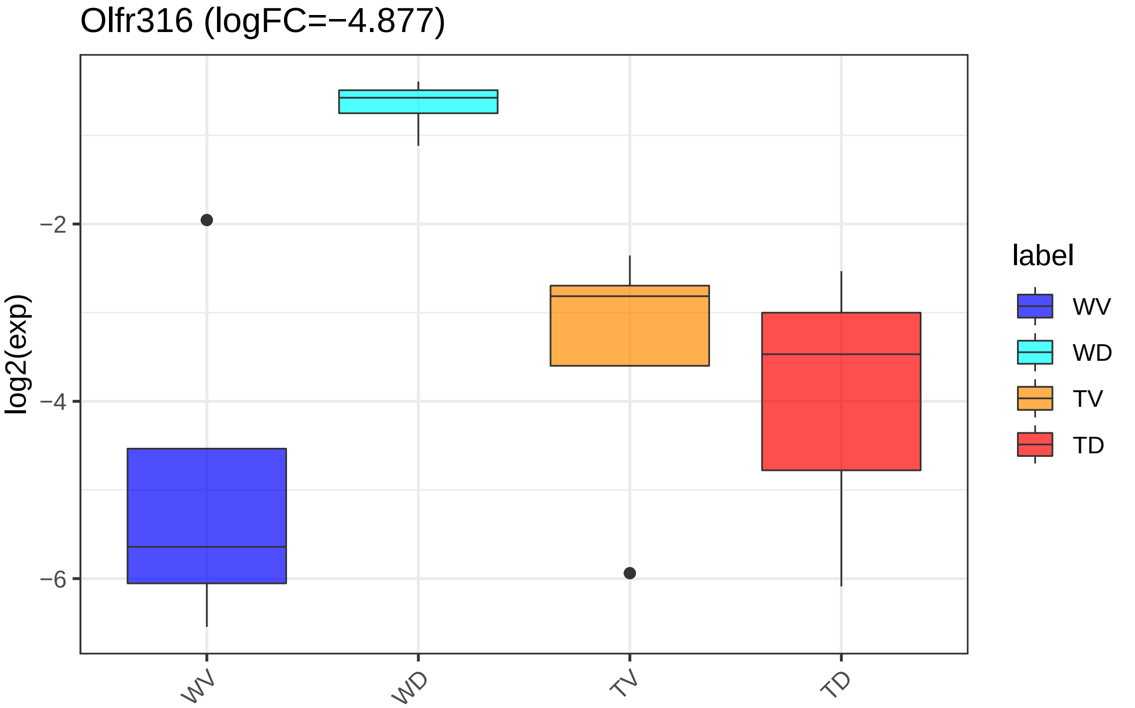

Olfr316 (logFC=−4.877)
log2(exp)
−2
−4
−6
WV
WD
TV
TD
label
WV
WD
TV
TD

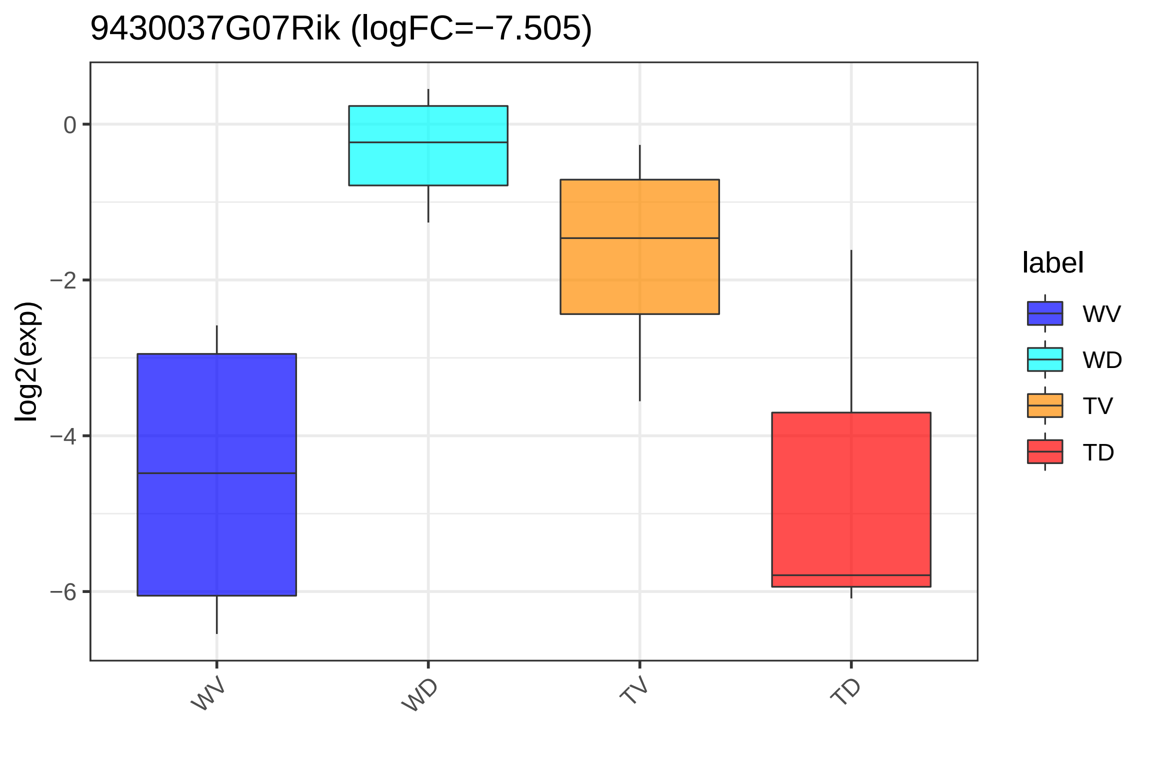

9430037G07Rik (logFC=−7.505)
log2(exp)
0
−2
−4
−6
WV
WD
TV
TD
label
WV
WD
TV
TD

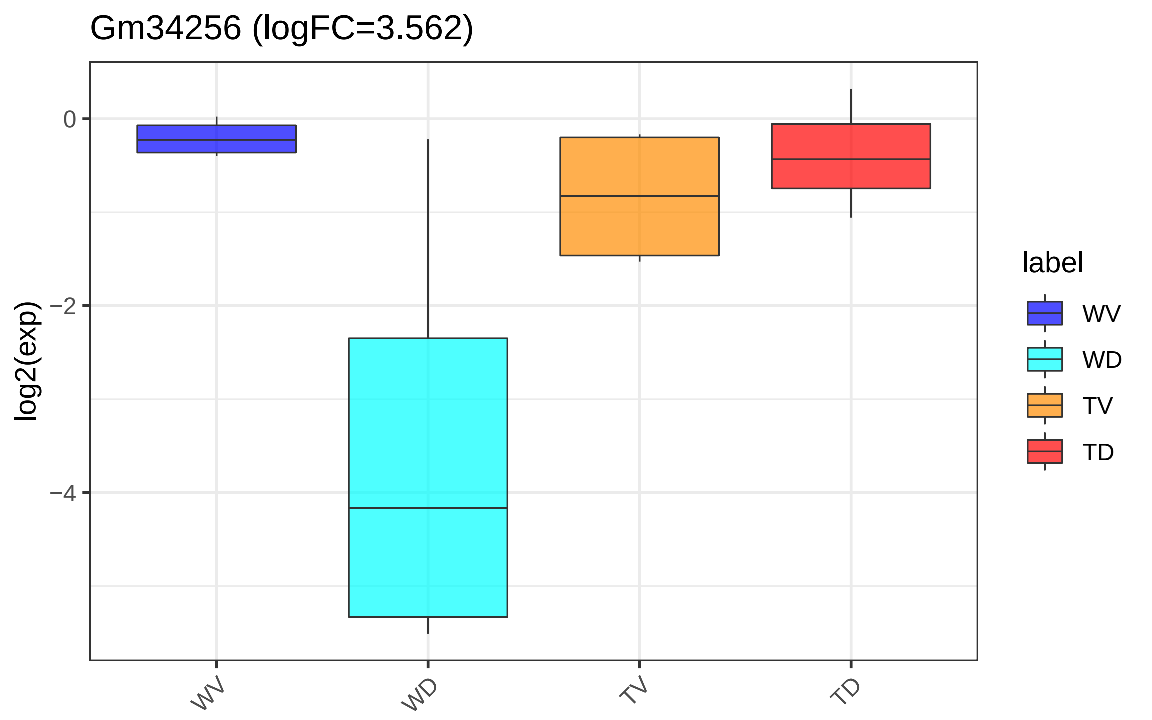
Gm34256 (logFC=3.562)
log2(exp)
0
−2
−4
WV
WD
TV
TD
label
WV
WD
TV
TD

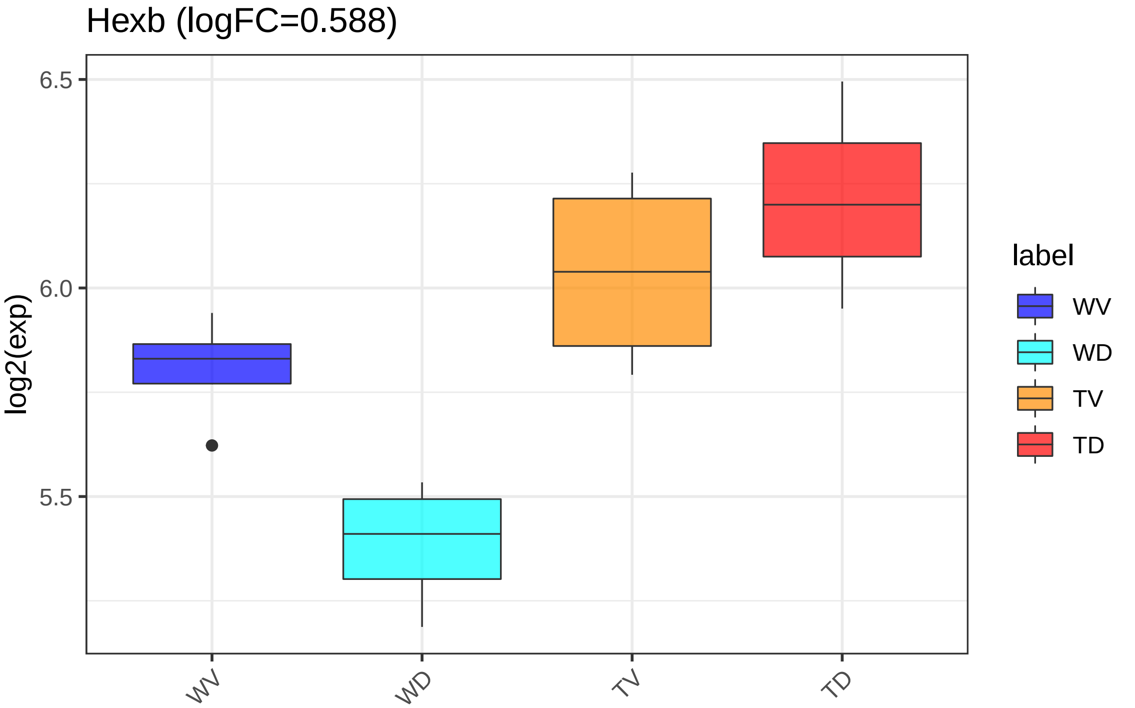
Hexb (logFC=0.588)
6.5
6.0
5.5
log2(exp)
WV
WD
TV
TD
label
WV
WD
TV
TD

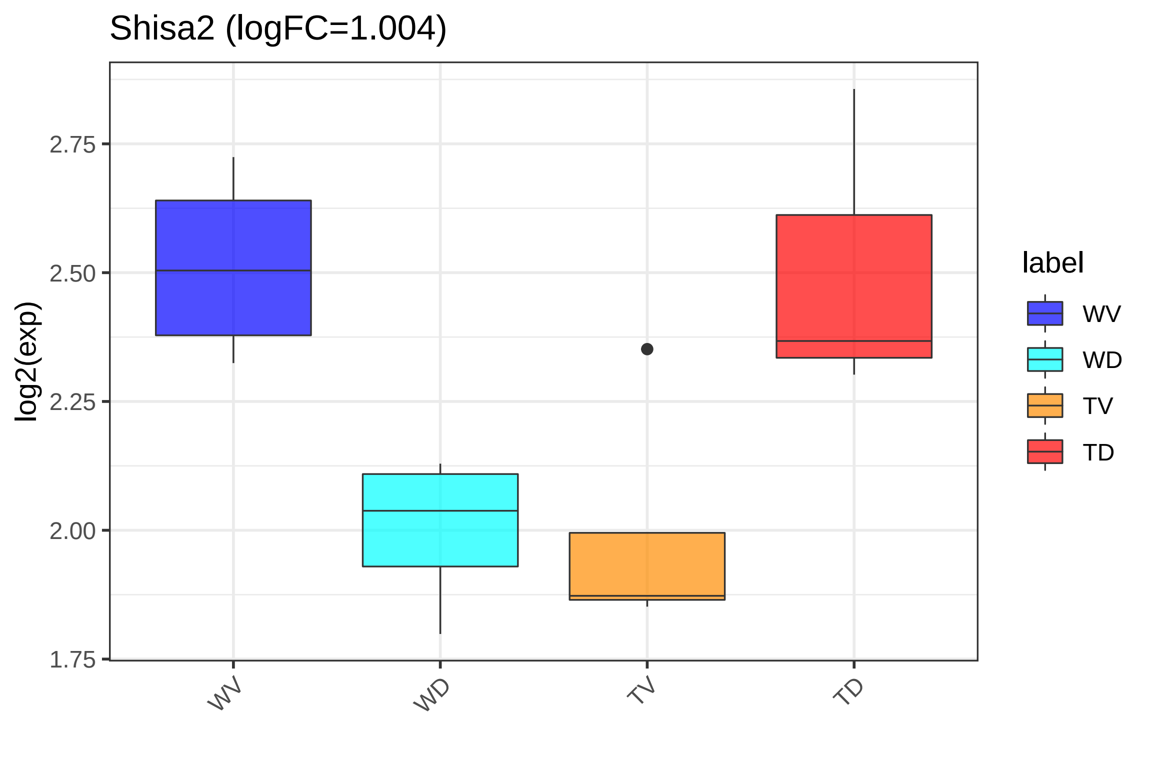
Shisa2 (logFC=1.004)
log2(exp)
2.75
2.50
2.25
2.00
1.75
WV
WD
TV
TD
label
WV
WD
TV
TD

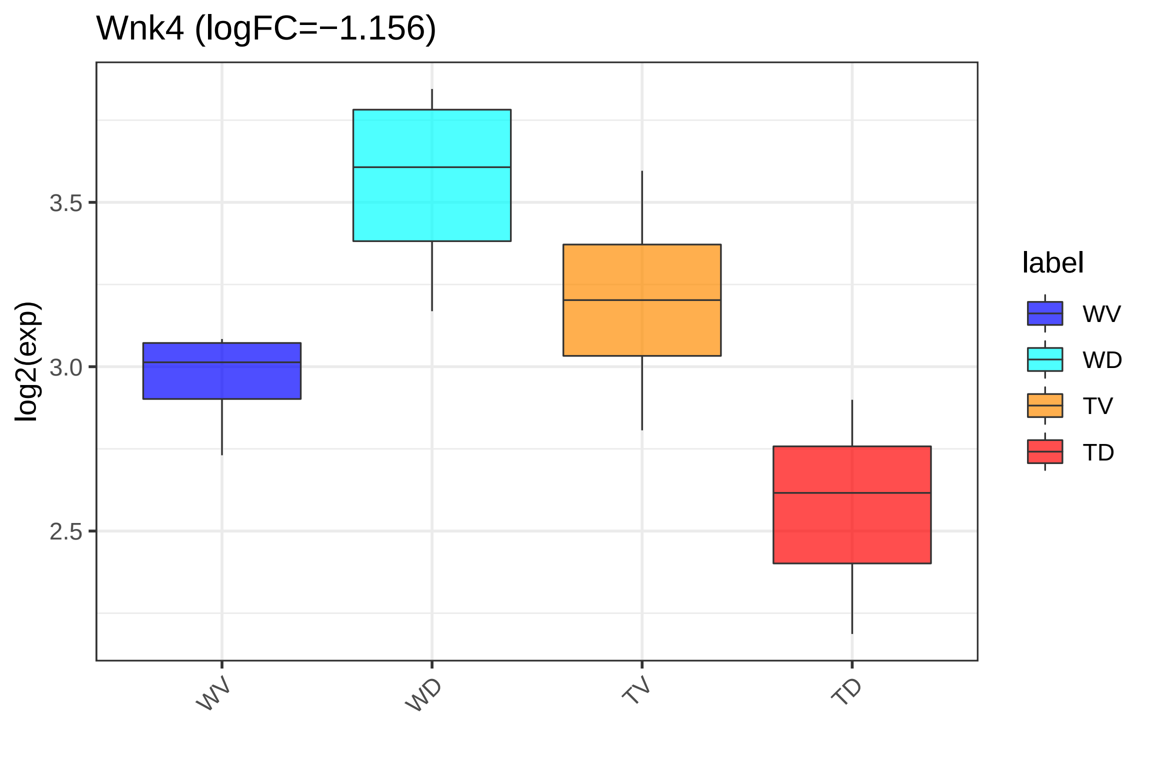
Wnk4 (logFC=−1.156)
log2(exp)
3.5
3.0
2.5
WV
WD
TV
TD
label
WV
WD
TV
TD

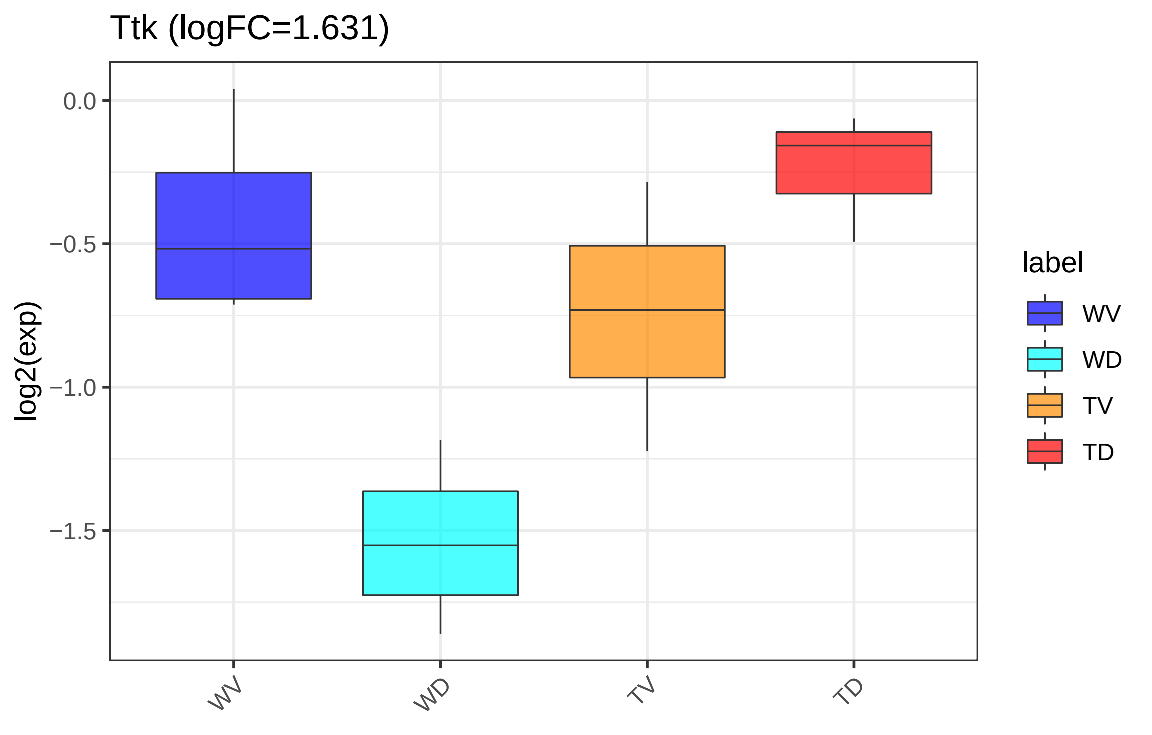

Ttk (logFC=1.631)
log2(exp)
0.0
−0.5
−1.0
−1.5
WV
WD
TV
TD
label
WV
WD
TV
TD

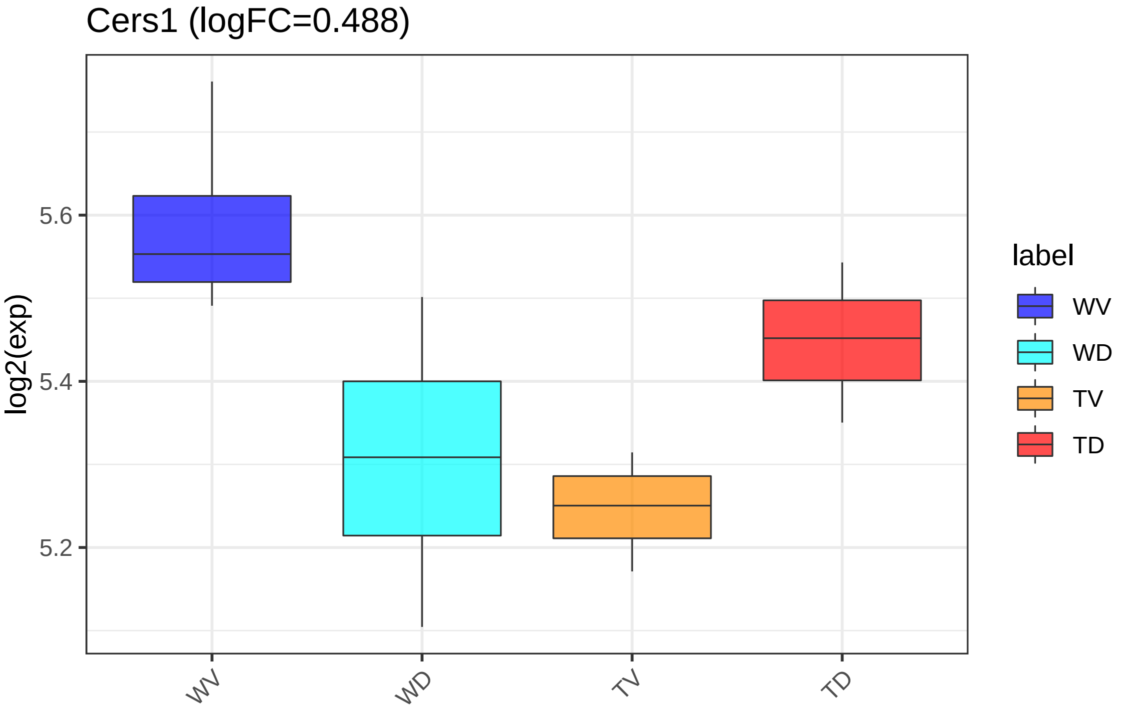

Cers1 (logFC=0.488)
log2(exp)
5.6
5.4
5.2
WV
WD
TV
TD
label
WV
WD
TV
TD

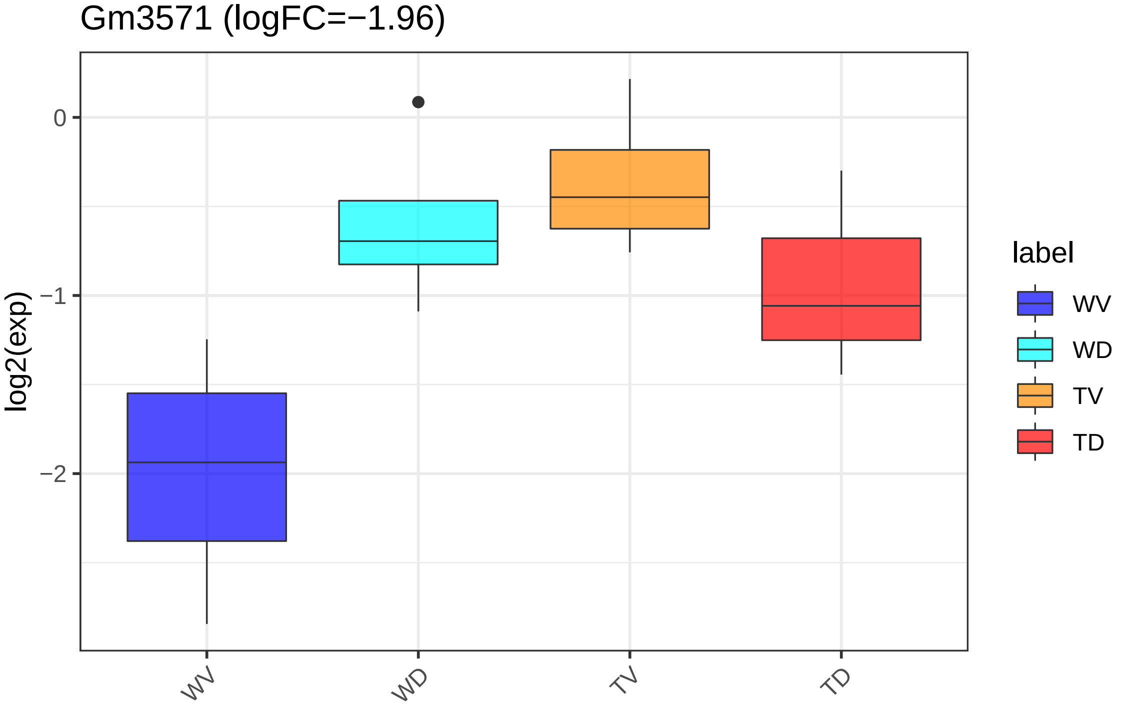
Gm3571 (logFC=−1.96)
log2(exp)
0
−1
−2
WV
WD
TV
TD
label
WV
WD
TV
TD

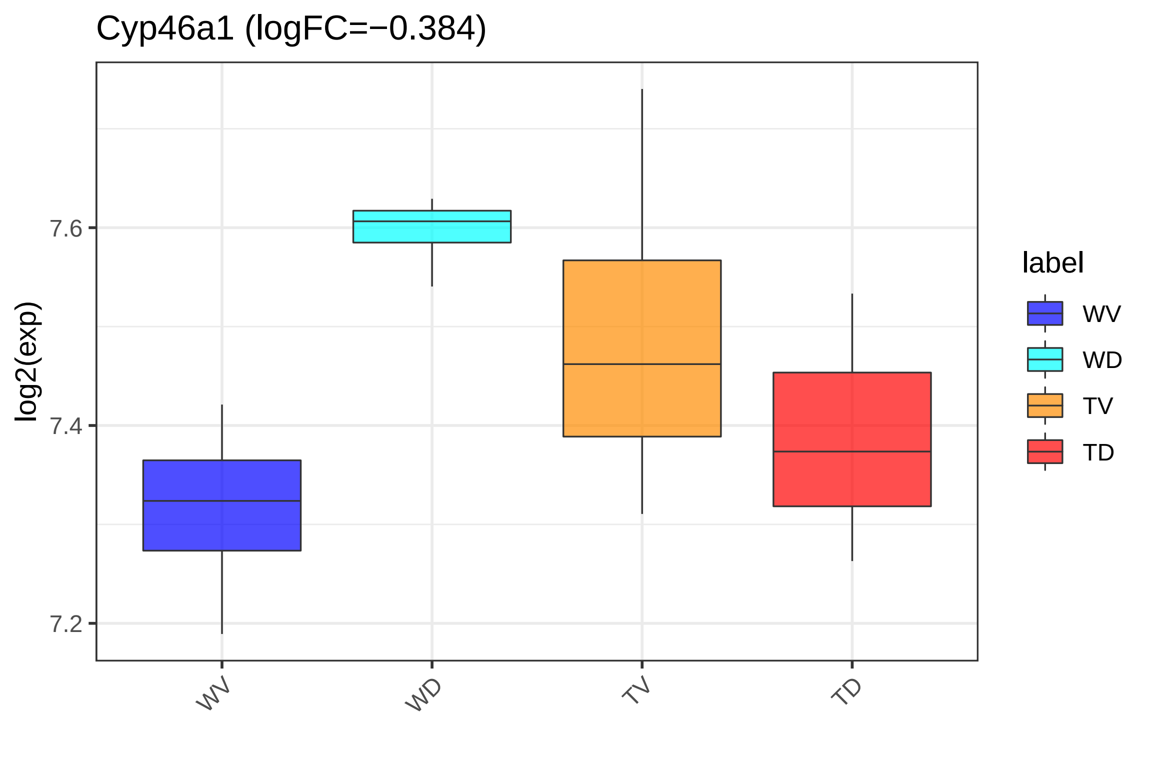

Cyp46a1 (logFC=−0.384)
log2(exp)
7.6
7.4
7.2
WV
WD
TV
TD
label
WV
WD
TV
TD

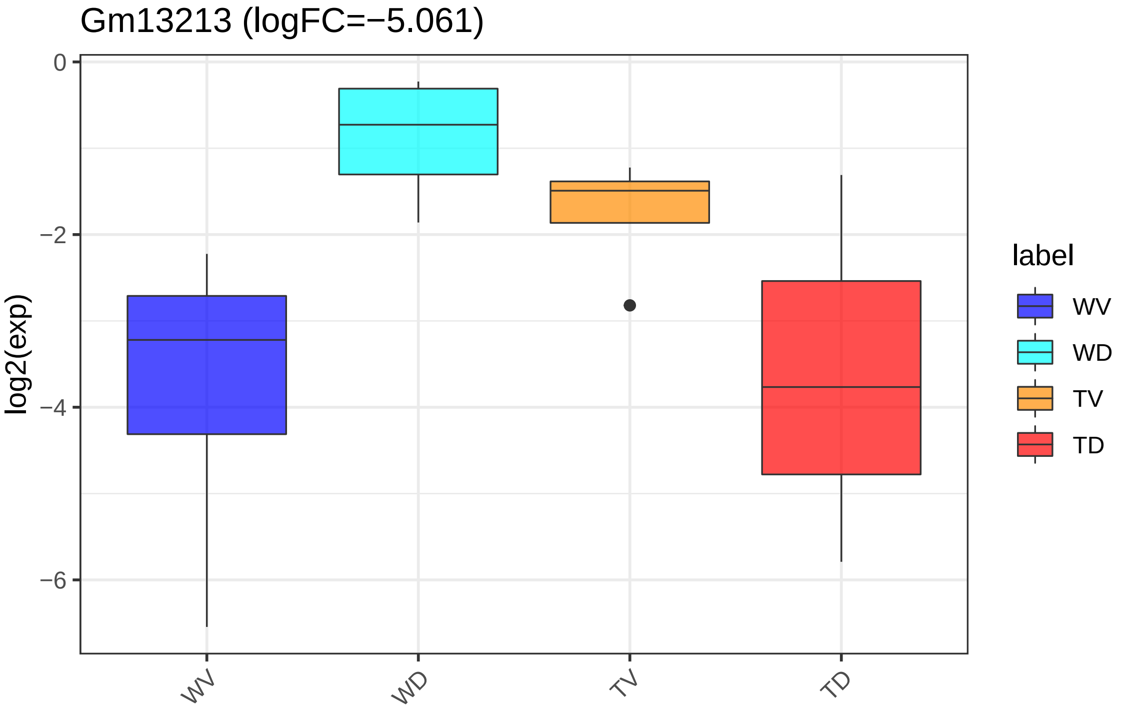

Gm13213 (logFC=−5.061)
log2(exp)
0
−2
−4
−6
WV
WD
TV
TD
label
WV
WD
TV
TD

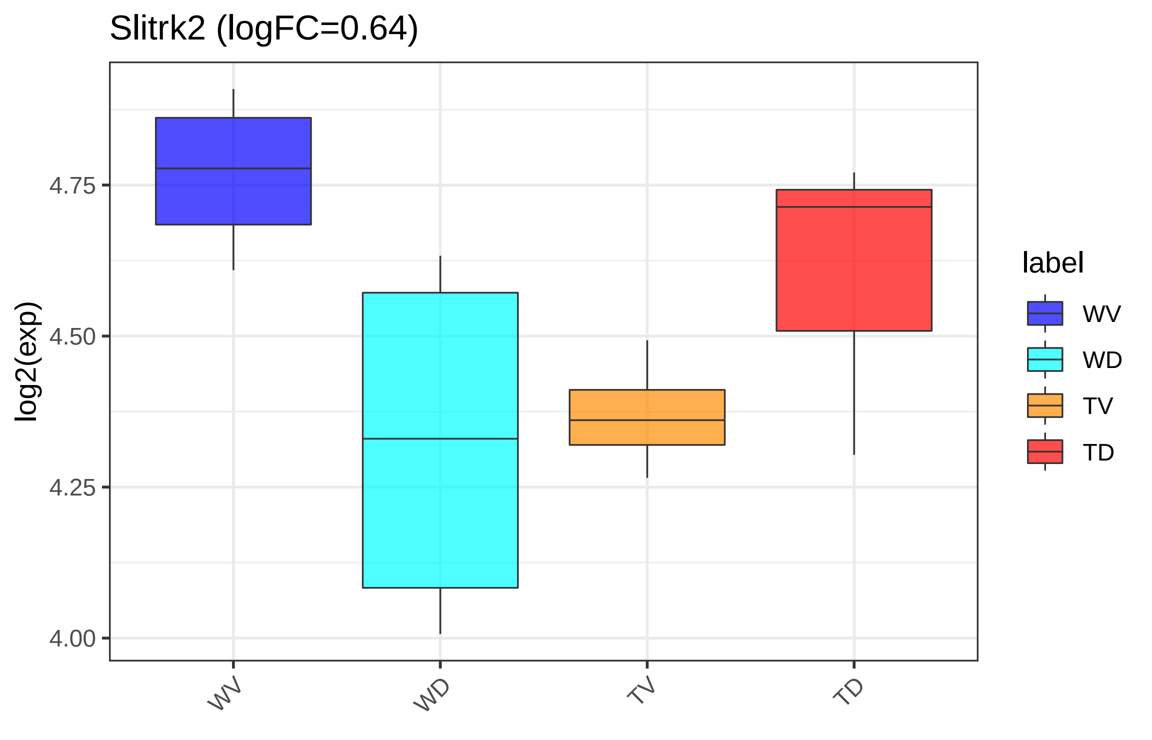

Slitrk2 (logFC=0.64)
log2(exp)
4.75
4.50
4.25
4.00
WV
WD
TV
TD
label
WV
WD
TV
TD

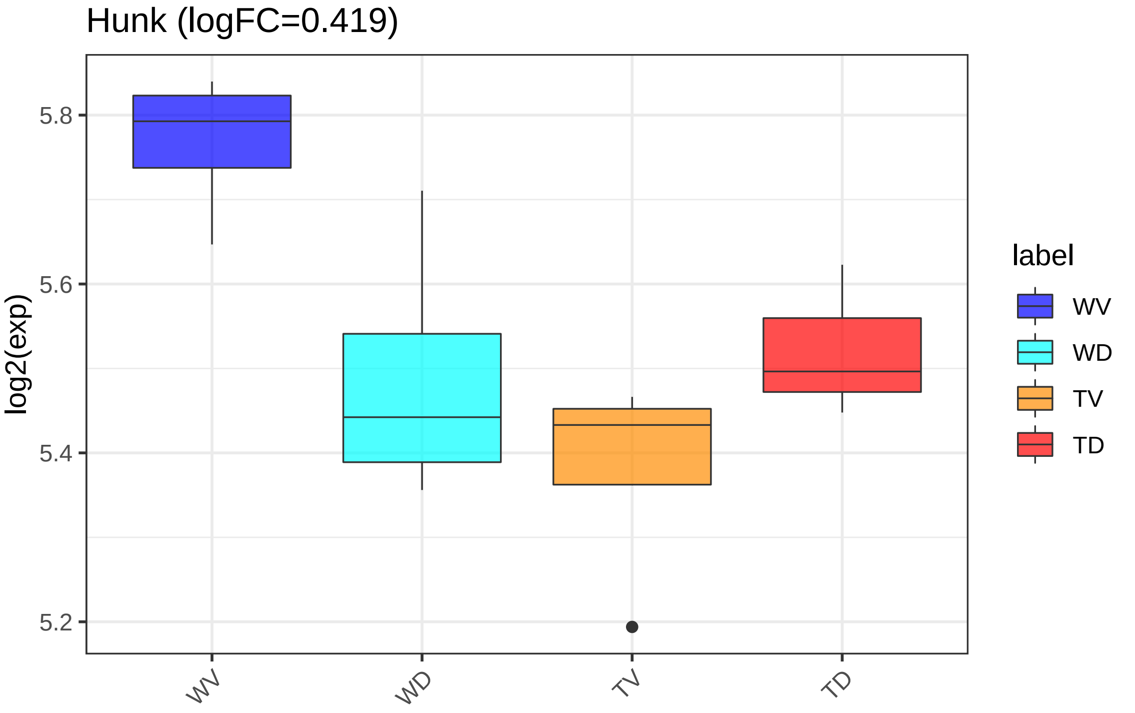
Hunk (logFC=0.419)
log2(exp)
5.8
5.6
5.4
5.2
WV
WD
TV
TD
label
WV
WD
TV
TD

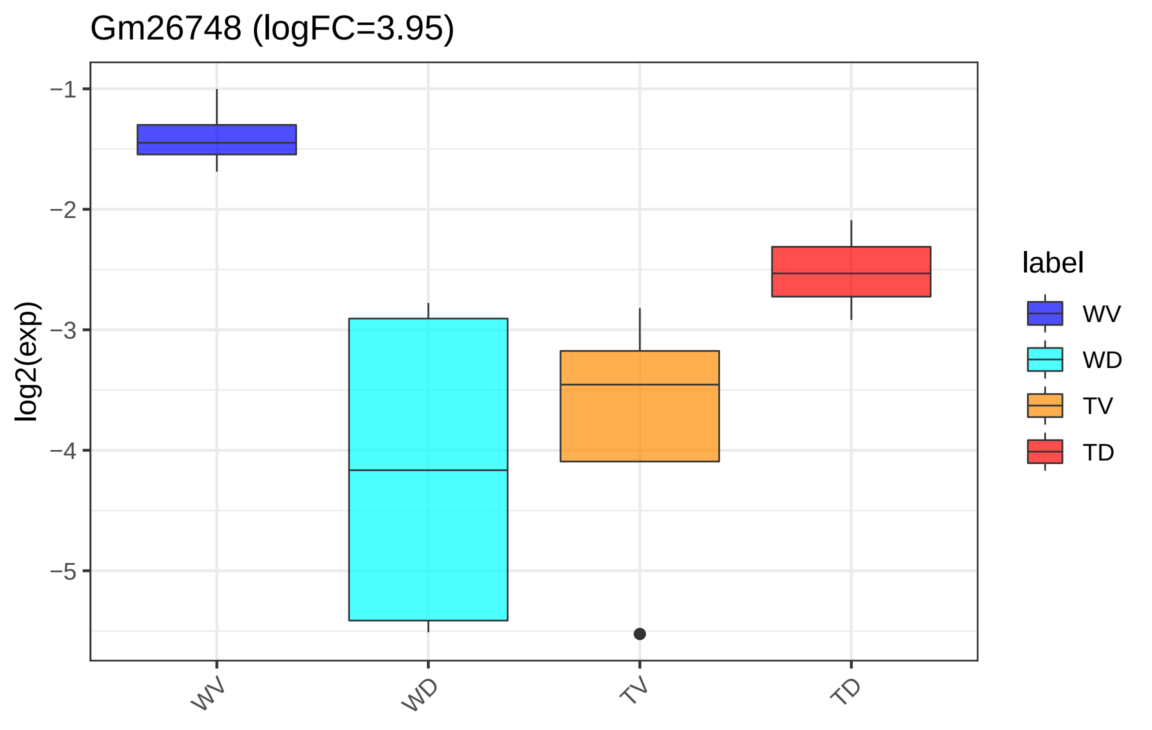

Gm26748 (logFC=3.95)
log2(exp)
−1
−2
−3
−4
−5
WV
WD
TV
TD
label
WV
WD
TV
TD

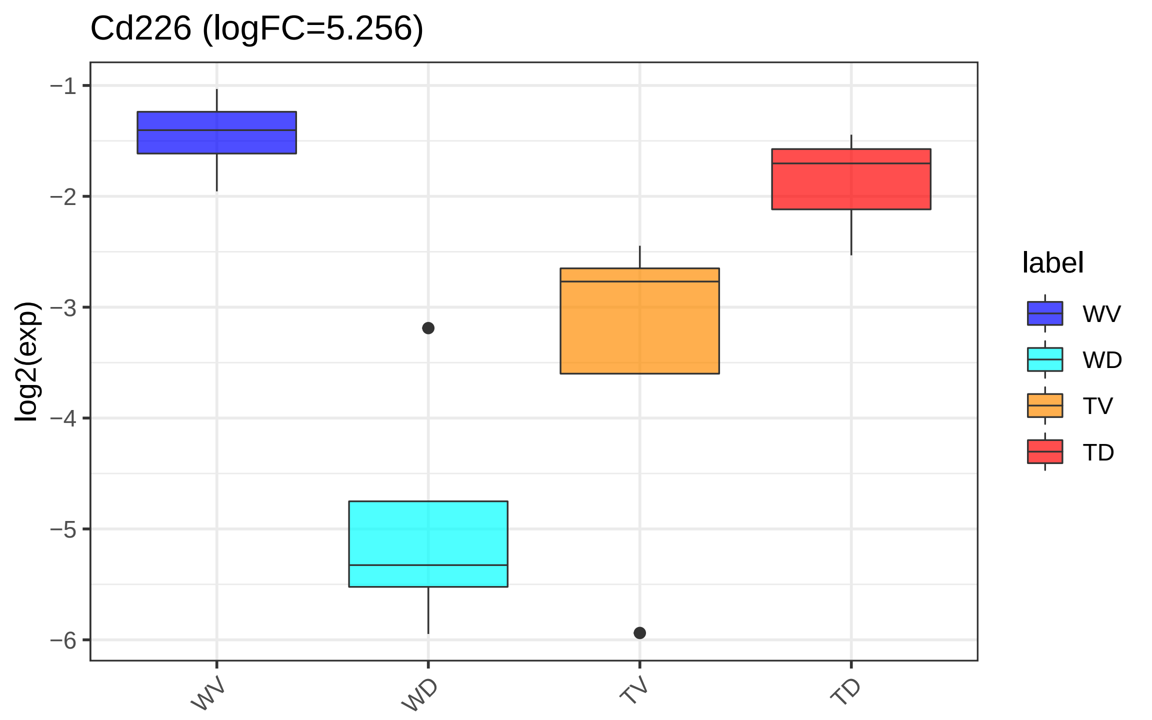
Cd226 (logFC=5.256)
log2(exp)
−1
−2
−3
−4
−5
−6
WV
WD
TV
TD
label
WV
WD
TV
TD

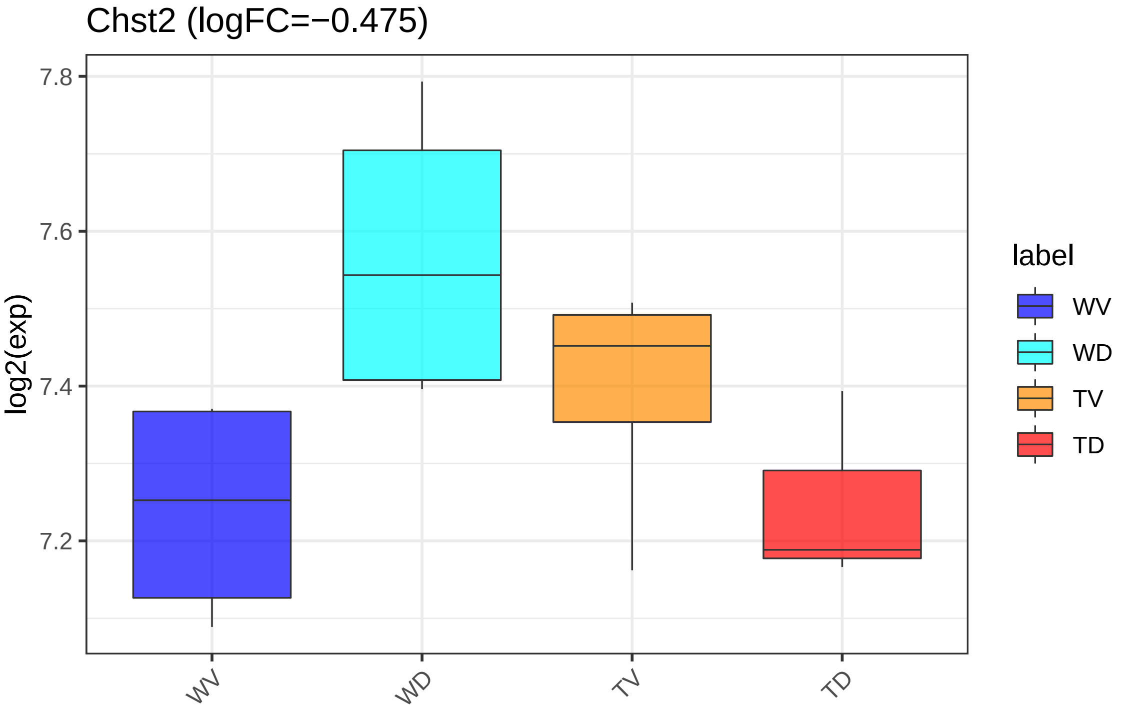

Chst2 (logFC=−0.475)
log2(exp)
7.8
7.6
7.4
7.2
WV
WD
TV
TD
label
WV
WD
TV
TD

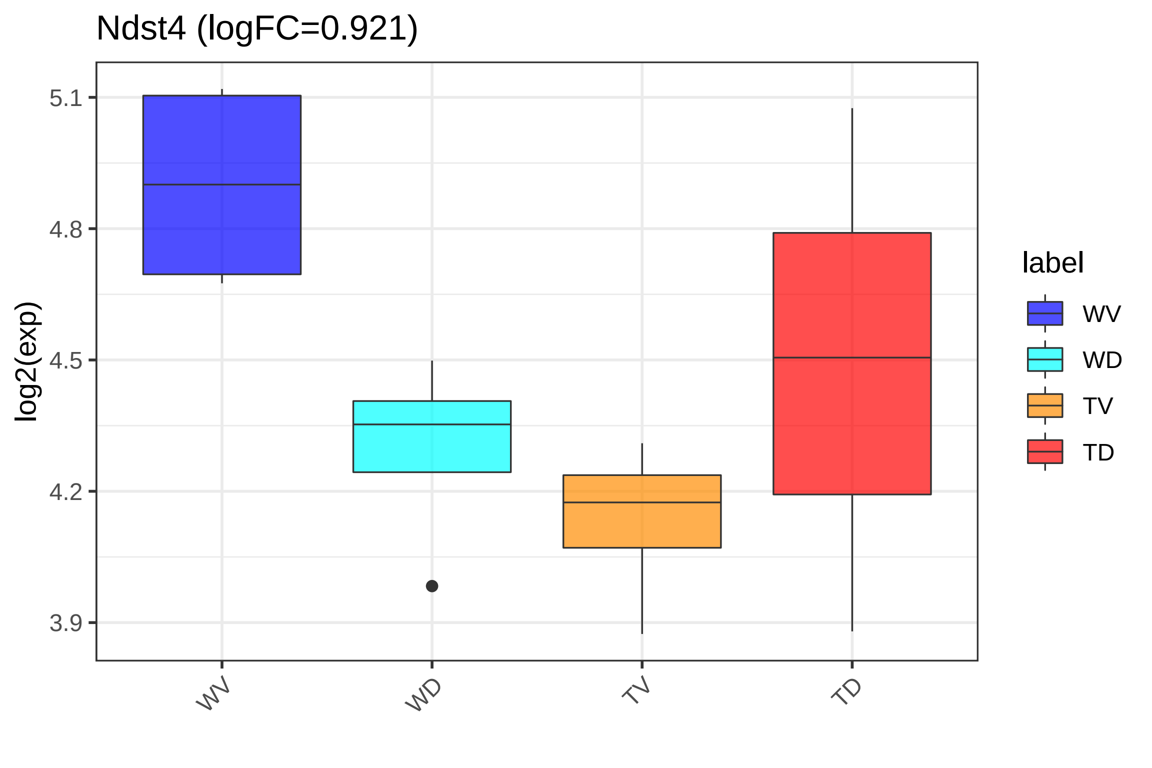

Ndst4 (logFC=0.921)
log2(exp)
5.1
4.8
4.5
4.2
3.9
WV
WD
TV
TD
label
WV
WD
TV
TD

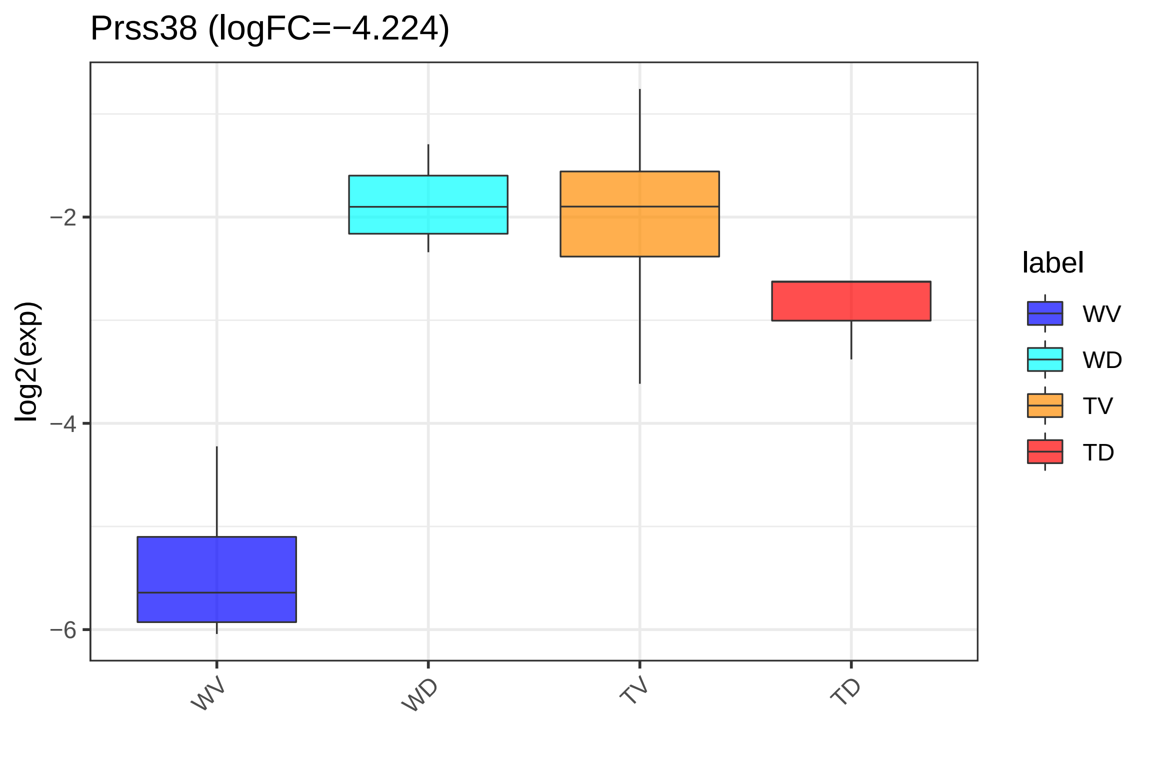

Prrs38 (logFC=−4.224)
log2(exp)
−2
−4
−6
WV
WD
TV
TD
label
WV
WD
TV
TD

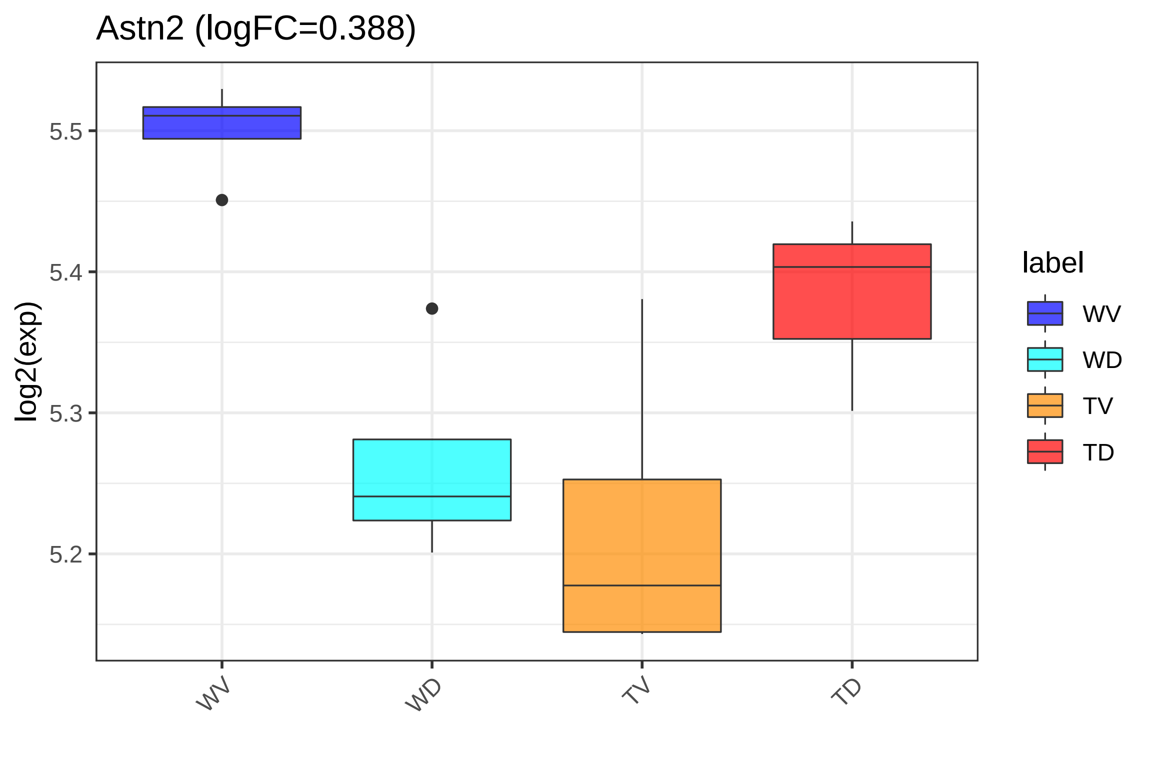

Astn2 (logFC=0.388)
log2(exp)
5.5
5.4
5.3
5.2
WV
WD
TV
TD
label
WV
WD
TV
TD

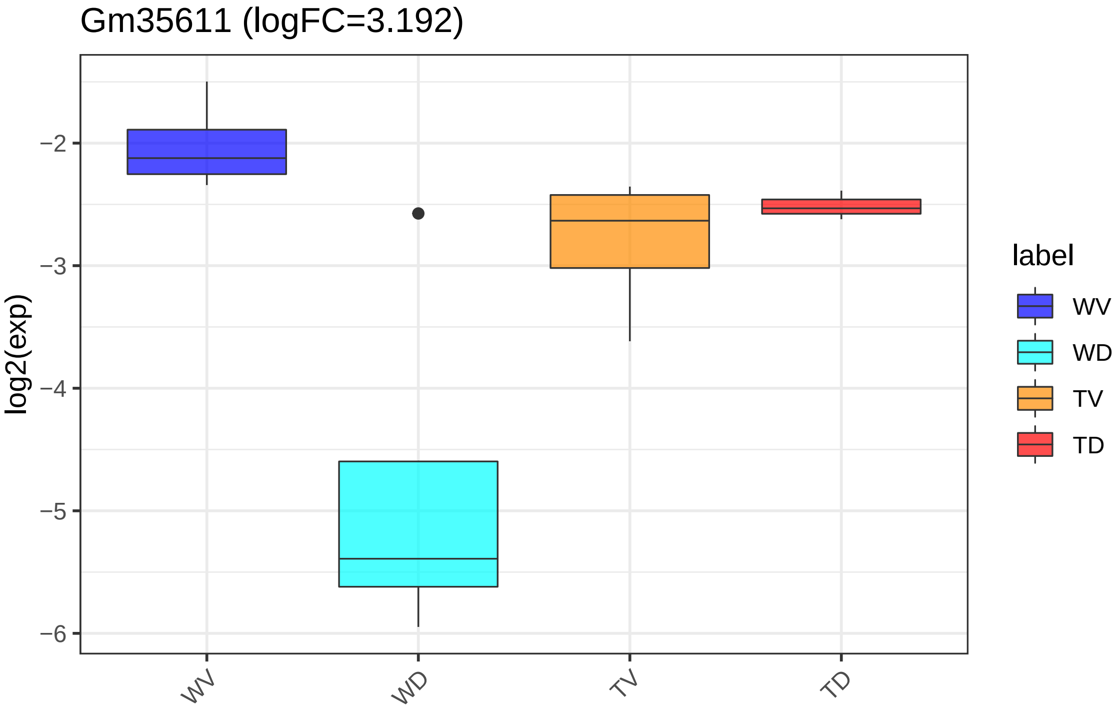

Gm35611 (logFC=3.192)
log2(exp)
−2
−3
−4
−5
−6
WV
WD
TV
TD
label
WV
WD
TV
TD

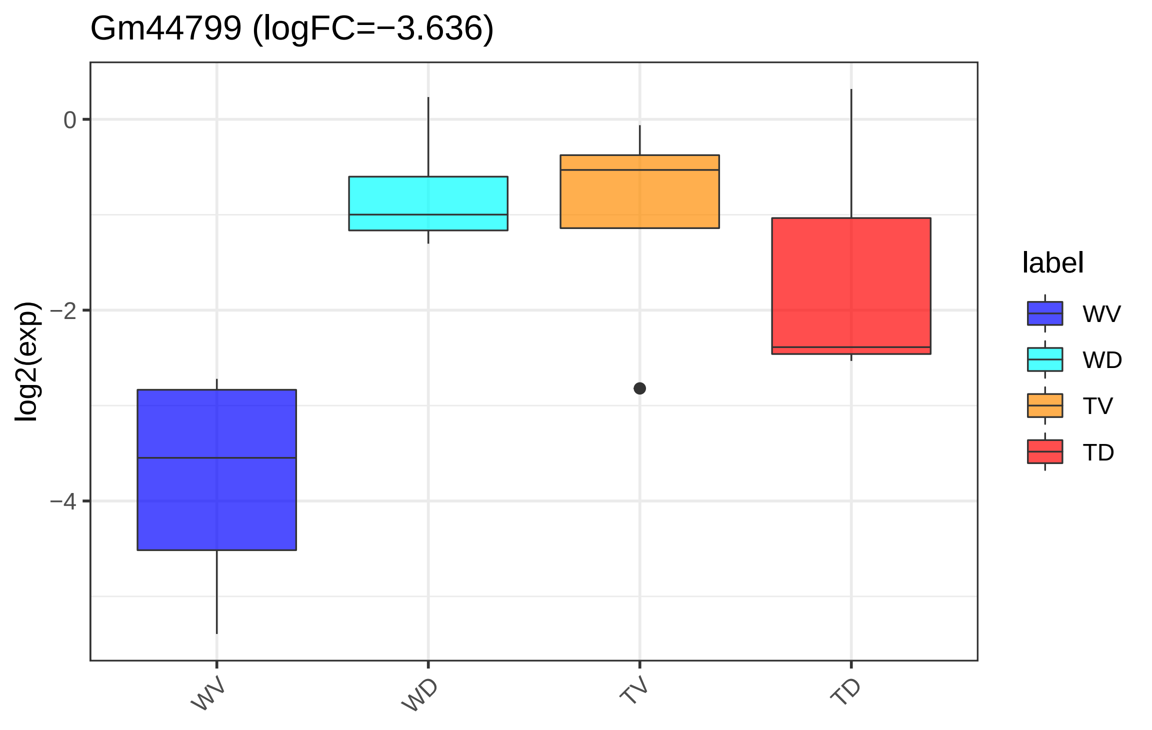

Gm44799 (logFC=−3.636)
log2(exp)
0
−2
−4
WV
WD
TV
TD
label
WV
WD
TV
TD

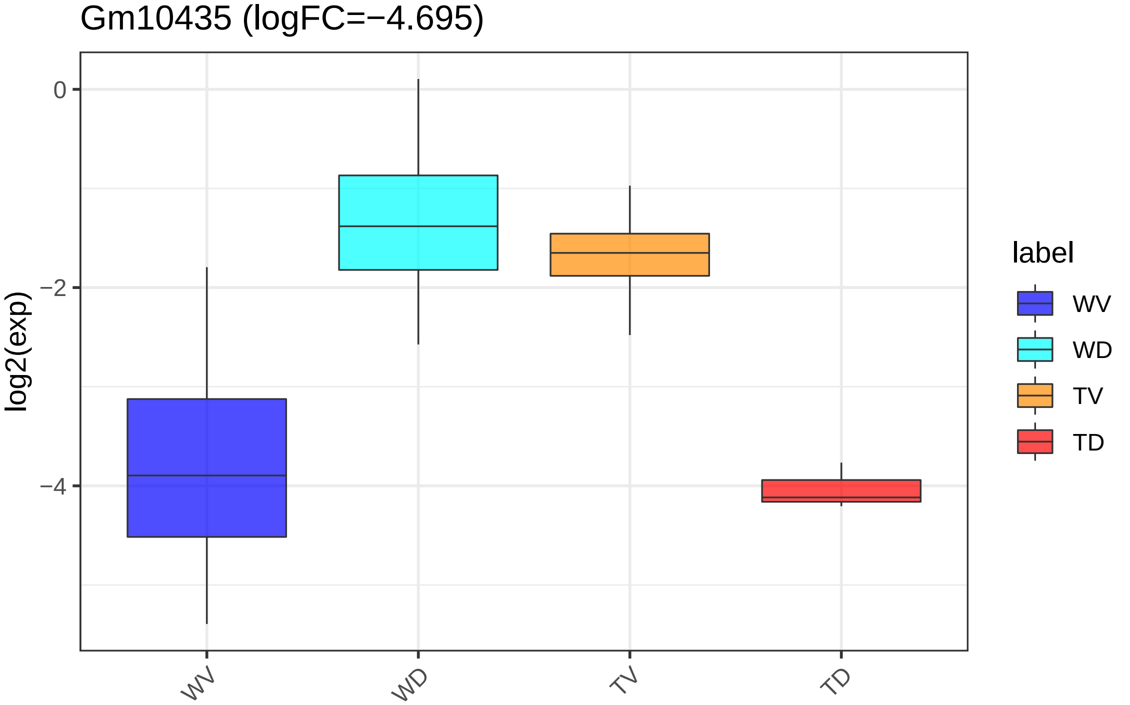
Gm10435 (logFC=−4.695)
log2(exp)
0
−2
−4
WV
WD
TV
TD
label
WV
WD
TV
TD

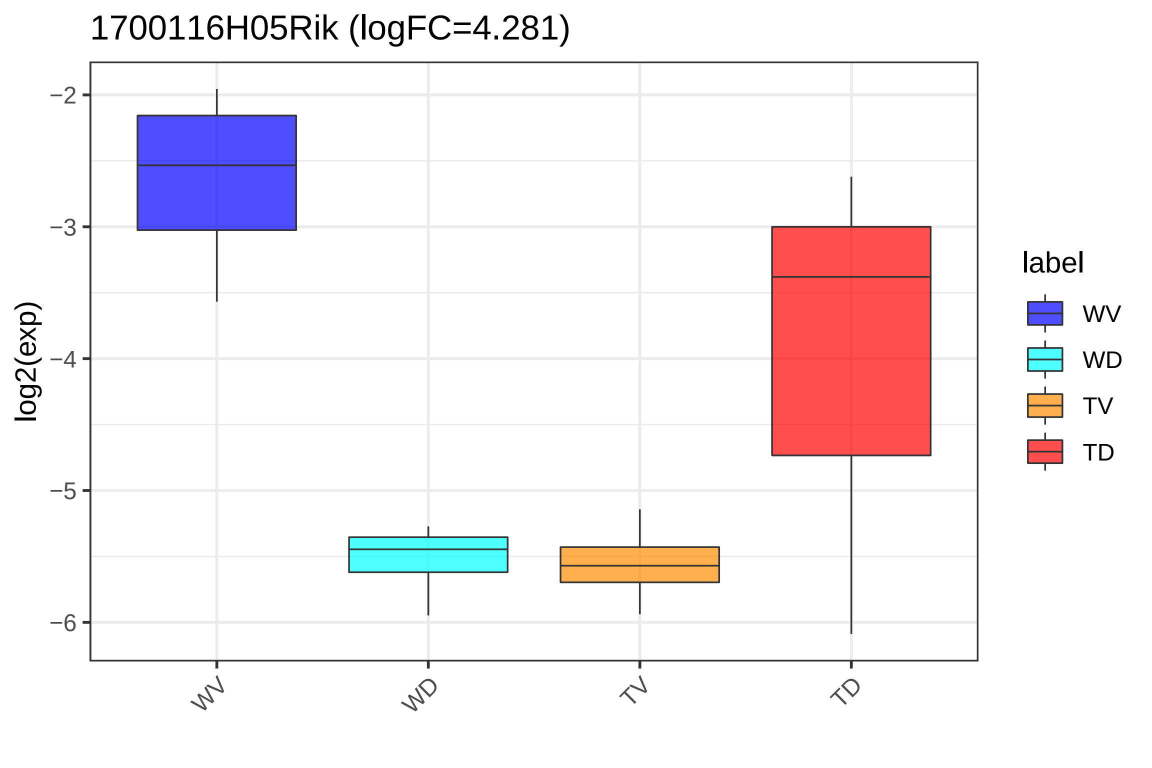
1700116H05Rik (logFC=4.281)
log2(exp)
−2
−3
−4
−5
−6
WV
WD
TV
TD
label
WV
WD
TV
TD

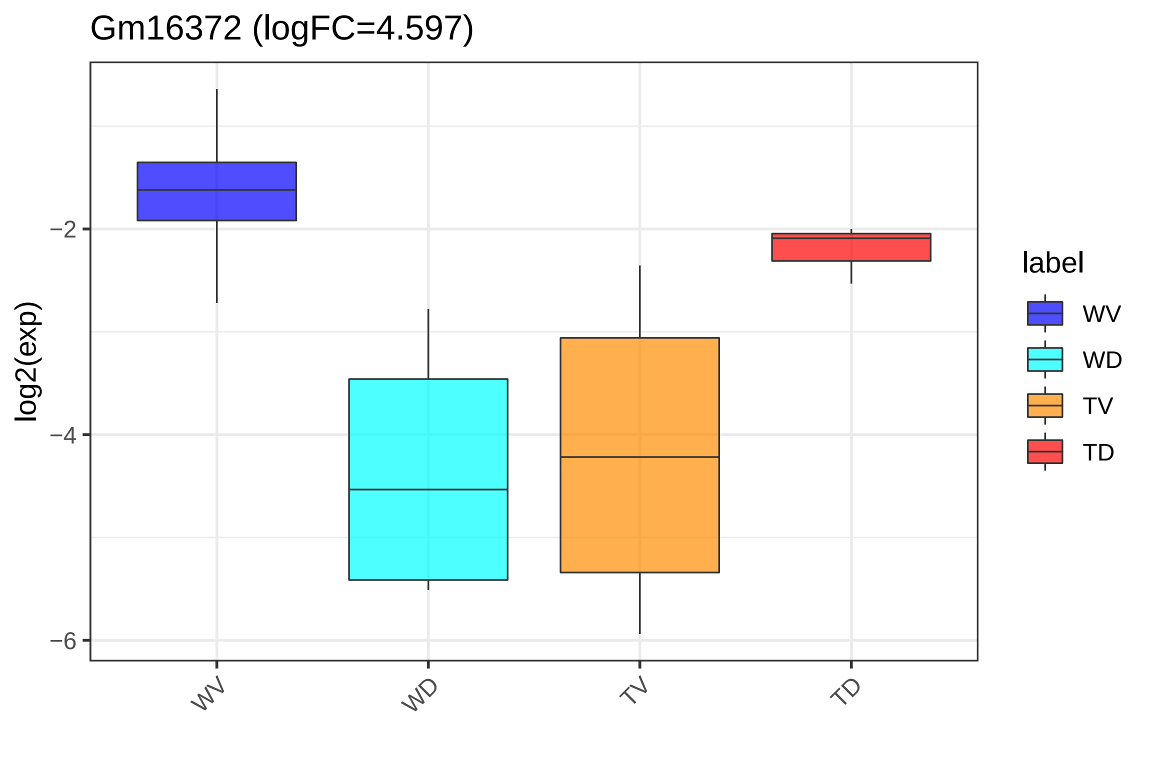
Gm16372 (logFC=4.597)
log2(exp)
−2
−4
−6
WV
WD
TV
TD
label
WV
WD
TV
TD

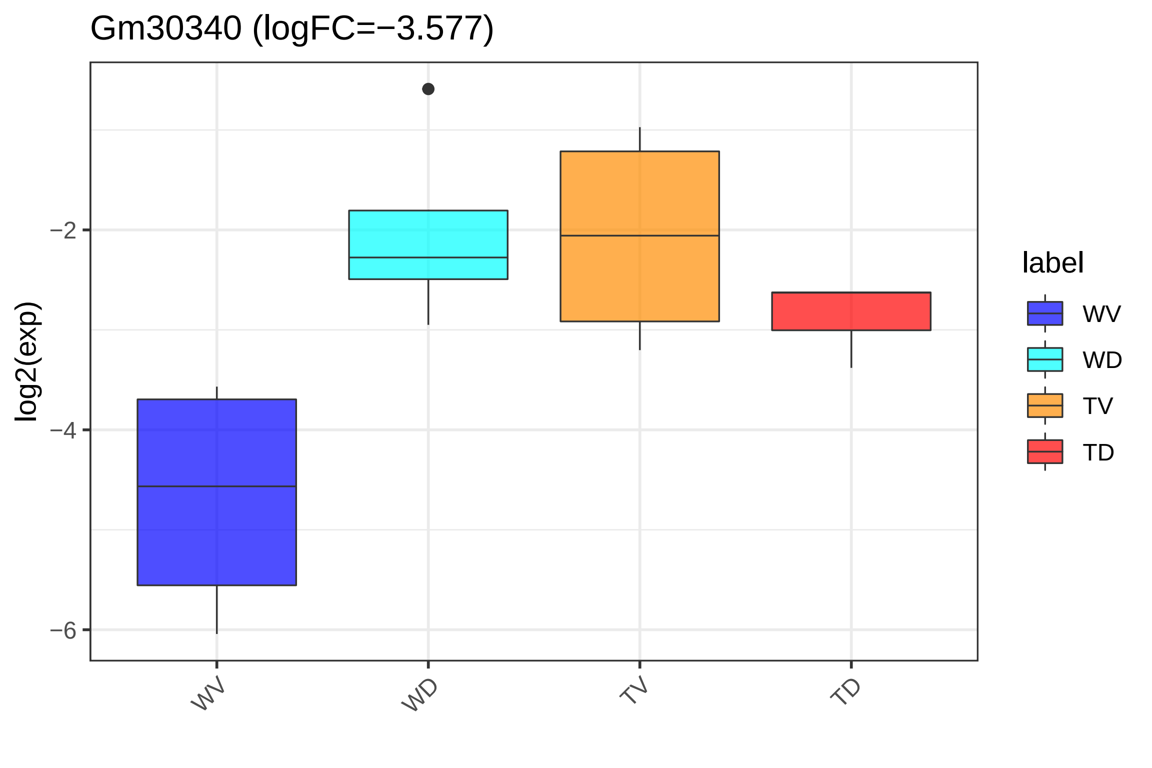

Gm30340 (logFC=−3.577)
log2(exp)
−2
−4
−6
WV
WD
TV
TD
label
WV
WD
TV
TD

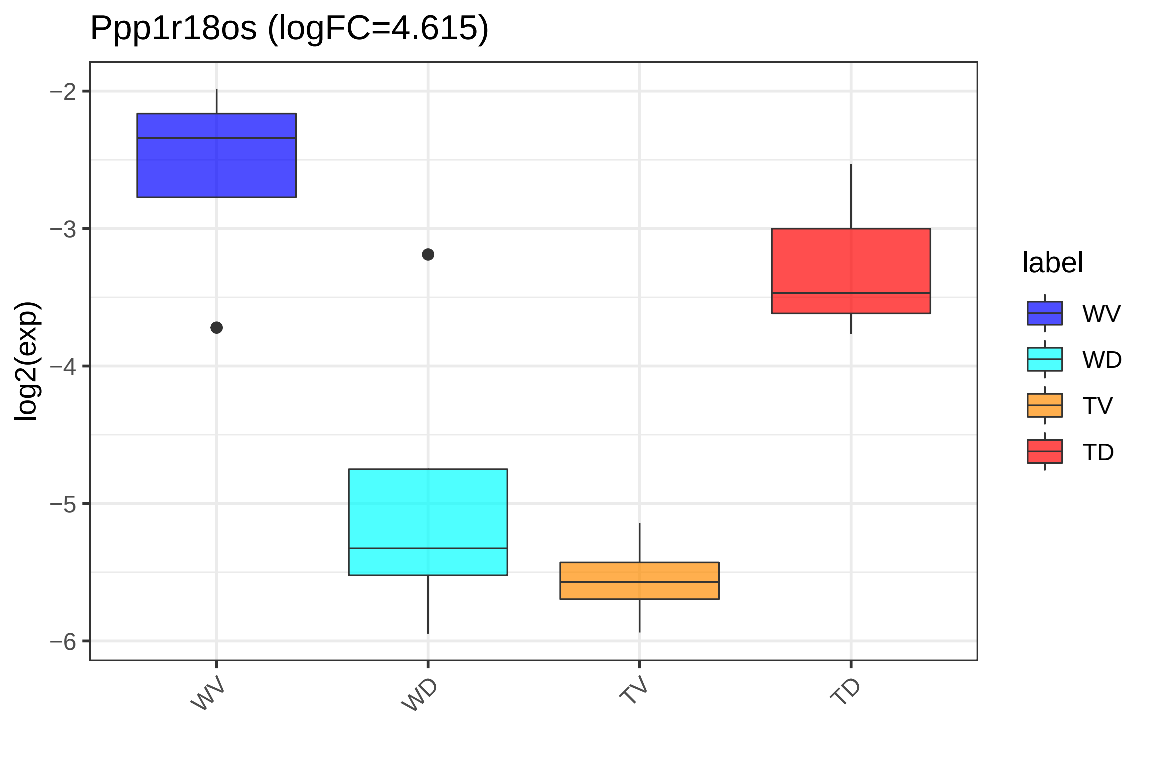
Ppp1r18os (logFC=4.615)
log2(exp)
−2
−3
−4
−5
−6
WV
WD
TV
TD
label
WV
WD
TV
TD
